# C/EBPβ-induced alternative splicing of RCAN1 generates a potent TCR-T target in mesenchymal glioblastoma

**DOI:** 10.1038/s41423-025-01360-0

**Published:** 2025-12-23

**Authors:** Zujian Xiong, Qinglin Kong, Bhuvitha Chagantipati, Amelia Stepniak, Ambika P. Jaswal, Chaim T. Sneiderman, Yuanyuan Han, Sydney A. Jackson, Rebecca A. Raphael, Wei Zhang, Muzi Li, Yapeng Chao, Bin Qin, Zeynep Dulkadir, Lance Schwegman, Yihao Zhang, Chloe Kuminkoski, Megan A. Mahlke, Poulomi Nath, Baoli Hu, Pascal O. Zinn, Megan Mantica, Sameer Agnihotri, Yael Nechemia-Arbely, Ian F. Pollack, Lora H. Rigatti, Thomas G. Forsthuber, Xuejun Li, Itay Raphael, Gary Kohanbash

**Affiliations:** 1 Department of Neurosurgery, Xiangya Hospital, Central South University, Changsha, Hunan China; 2 Department of Neurological Surgery, University of Pittsburgh, Pittsburgh, PA USA; 3 Department of Radiology, Weill Cornell Medical College, New York, NY USA; 4 Department of Nephrology, Xiangya Hospital, Central South University, Changsha, Hunan China; 5 Department of Bioengineering, University of Pittsburgh, Pittsburgh, PA USA; 6 UPMC Hillman Cancer Center, Pittsburgh, PA USA; 7 Institute for Drug Research (IDR), School of Pharmacy, Faculty of Medicine, The Hebrew University of Jerusalem, Jerusalem, Israel; 8 The Second School of Clinical Medicine, Anhui Medical University, Hefei, Anhui China; 9 National Center for International Research in Cell and Gene Therapy, Sino-British Research Center for Molecular Oncology, Academy of Medical Sciences, Zhengzhou University, Zhengzhou, Henan, China; 10 Department of Molecular Microbiology and Immunology, University of Texas at San Antonio, San Antonio, TX USA; 11 Department of Pharmacology & Chemical Biology, University of Pittsburgh School of Medicine, Pittsburgh, PA USA; 12 Department of Neurology, University of Pittsburgh, Pittsburgh, PA USA; 13 Division of Laboratory Animal Resources, University of Pittsburgh School of Medicine, Pittsburgh, PA USA; 14 Hunan International Scientific and Technological Cooperation Base of Brain Tumor Research, Xiangya Hospital, Central South University, Changsha, China; 15 Department of Immunology, University of Pittsburgh, Pittsburgh, PA USA

**Keywords:** GBM, Antigen detection, Alternative splicing, TCR-T, RCAN1, Immunotherapy, CNS cancer, Cancer immunotherapy

## Abstract

Glioblastoma (GBM) is an aggressive brain tumor with limited treatment options and a dismal prognosis. While immunotherapy has shown promise in treating some solid tumors, the treatment of GBM has been mostly unsuccessful because of a lack of targetable tumor antigens and high tumor heterogeneity. Here, we report RCAN1-4 as a novel tumor antigen derived from alternative splicing induced by the transcription factor C/EBPβ. Both C/EBPβ and RCAN1-4 are highly expressed in GBM and glioma stem cells as mesenchymal subtype hallmarks. We report an immunogenic HLA-A24-specific splicing junction epitope within exon 4 and exon 5 that is unique to RCAN1-4. This epitope was validated for its ability to stimulate T cell responses in HLA-A24^+^ donors and GBM patients, leading us to identify RCAN1-4-reactive T cell receptors (TCRs) for the construction of TCR-engineered T cells (TCR-T cells). Functional studies of TCR-Ts demonstrated the in vitro and in vivo killing of RCAN1-4^pos^ GBM tumor cells, highlighting its potential as an immunotherapeutic target in mesenchymal GBM.

## Introduction

Gliomas are common primary brain malignancies and account for approximately 80% of brain tumors in adults [1] and 50% in children [2]. In accordance with the updated World Health Organization 5th classification of tumors of the central nervous system, diffuse gliomas represent a subset of gliomas and are divided into three families: adult-type diffuse gliomas, pediatric-type diffuse low-grade gliomas (LGGs), and pediatric-type diffuse high-grade gliomas (HGGs, including pediatric glioblastoma (GBM)) [3]. Adult-type gliomas are distinguished on the basis of IDH status: IDH-mutated astrocytoma/oligodendroglioma (previously known as LGG) and IDH-wild-type GBM. Recent studies have demonstrated that adult GBM contains three molecular subtypes, namely, the classical, mesenchymal, and proneural subtypes, with the mesenchymal subtype being the most aggressive [4, 5]. Unlike adult GBM, pediatric GBM subtype classification is primarily based on specific genetic alterations [6, 7].

Alternative splicing (AS) is a prevalent posttranscriptional mechanism that combines exons into heterogeneous nuclear RNA (“premature” hnRNA) transcribed from DNA. This generates diverse mature mRNAs, also known as gene isoforms, ultimately resulting in distinct protein products from a single gene [8]. Nearly 95% of multiexon genes undergo AS [9], and tumors generally display up to 30% more splicing events than normal tissues do [10]. Within tumors, these aberrant splicing events shift the relative abundance of gene transcripts, termed isoform switching, and generate distinct protein isoforms [11]. Increased isoform switching is mediated by the dysregulated function of RNA-binding proteins through a variety of mechanisms, such as cis-acting mutations in splicing junctions (SJs) or trans-acting alterations in splicing factors or transcription factors [10, 12, 13]. In GBM, there is a notable shift in the expression of many genes from their canonical expression patterns toward less common protein variants [10, 14, 15]. These aberrant splicing events accumulate further in recurrent GBM [16], thereby promoting GBM progression [17, 18].

Immunotherapy provokes the host immune system by overcoming immunosuppressive signals and/or enhancing tumor-specific cytotoxic T cell responses. Although immunotherapy has shown promising efficacy against various malignancies [19], progress has been limited in GBM [20]. A key challenge in developing effective immunotherapies for GBM is the identification of suitable tumor antigens. Previous studies targeting neoepitopes have shown some promise [21, 22] but have revealed important obstacles, such as loss of antigen expression, as observed with EGFRvIII [23, 24]. Furthermore, targeting neoepitopes, which are unique mutations in tumor cells, presents additional challenges, as only a fraction of mutations are processed and presented by HLA molecules for T cell recognition [25–27]. Moreover, the relatively low tumor mutation burden in GBM compared with other solid tumors creates challenges in identifying targetable neoepitopes [28, 29]. Additionally, the constantly evolving proteome due to tumor evolution in GBM [24], coupled with high tumor heterogeneity [30], results in a variable and dynamic antigen spectrum. Taken together, these issues pose significant obstacles for the development of effective and durable immunotherapies. Therefore, identifying additional targetable tumor antigens is an unmet need for developing novel immunotherapies against GBM.

In the present study, we hypothesized that aberrant SJs derived from isoform switching can generate novel immunogenic antigen epitopes in GBM. We showed that RCAN1-4 is the primary aberrant isoform of RCAN1 in GBM. High RCAN1-4 expression was induced by the binding of the transcription factor C/EBPβ to its transcription start site (TSS) near exon 4 of RCAN1 in malignant glioma, primarily in the mesenchymal GBM subtype. Furthermore, we identified an HLA-A24-restricted epitope, RCAN1-4_22-32_, positioned across the SJ of exon 4 and exon 5 of the RCAN1 gene in RCAN1-4. This epitope is highly immunogenic and generates antitumor T cell responses in both HLA-A24^+^ healthy donors and GBM patients. By cloning the RCAN1-4_22-32_-reactive T cell receptor (TCR) derived from single-cell sequencing, we constructed RCAN1-4_22-32_-specific TCR-engineered T cells (TCR-T cells) and validated their efficacy in killing tumor cells in vitro and reducing the tumor burden in vivo. Overall, we revealed that RCAN1-4 is a novel tumor-associated antigen (TAA) derived from aberrant AS for GBM immunotherapy.

## Results

### Upregulation of the RCAN1-4 isoform in GBM

**Fig. 1:**
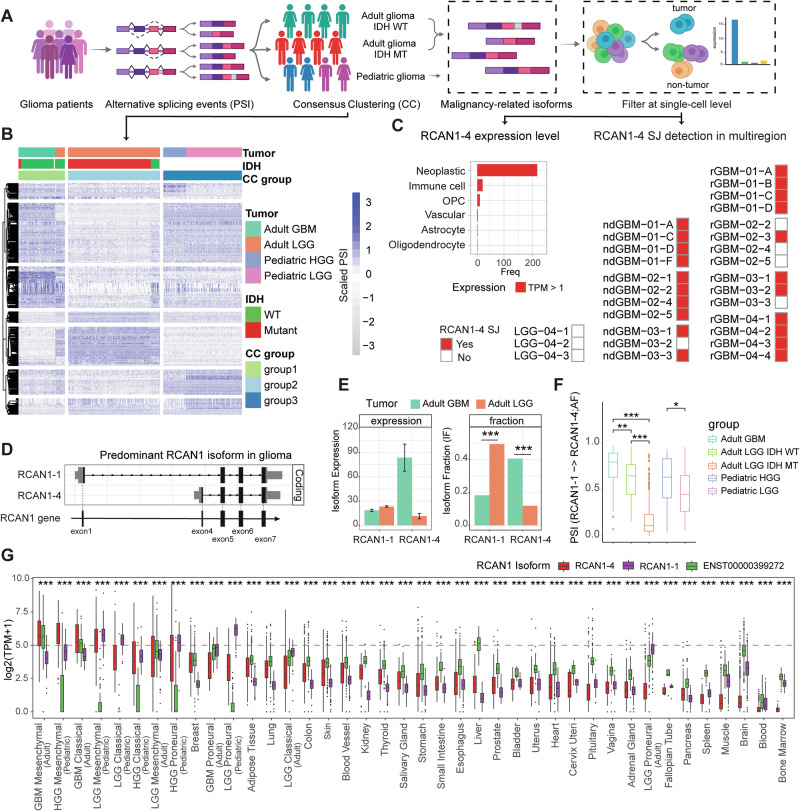
RCAN1-4 is upregulated in malignant glioma. **A** Schematic representation of malignancy-related isoform filtration in glioma patients. **B** Alternative splicing event pattern (row) of three consensus cluster groups (CC group, column) in glioma patients. **C** Left, RCAN1-4 expression in the adult GBM tumor microenvironment at the single-cell level. Cells with RCAN1-4 TPM > 1 were counted. Right, detection of the alternative splicing junction of RCAN1-4 in glioma cells from multiregional samples. Detection was performed by matching the scRNA-seq reads of each cell to the RCAN1-4 splicing junction between exon 4 and exon 5 of the RCAN1 gene. ndGBM newly diagnosed GBM, rGBM recurrent GBM. **D** Exon combinations of RCAN1 isoforms. **E** RCAN1 isoform expression and fraction changes between adult LGG and GBM patients. The fraction was calculated as the ratio of the expression of one isoform relative to gene expression. **F** PSI comparison of RCAN1 local alternative splicing events of the alternative first exon (AF) from exon 1 initiation to exon 4 initiation (RCAN1-1 to RCAN1-4) in different glioma groups. **G** Expression of RCAN1 predominant isoforms in gliomas and normal tissues. TPM > 33 is indicated by the dashed red line. Gliomas were classified according to GBM transcriptomic subtype characteristics. *p < 0.05; **p < 0.01; ***p < 0.001

To identify isoforms associated with the potential for malignancy in glioma, we first estimated AS events across glioma cohorts by the percent spliced-in (PSI) index from tumor-bulk RNA-seq data [31]. PSI results were then integrated with glioma type and patient clinical data to identify isoforms related to malignant gliomas, followed by expression assessment at the single-cell level (Fig. 1A). Following the exclusion of splicing events not detected across the entire glioma patient cohort (Supplementary Fig. 1A), we identified three distinct consensus clustering groups with AS signatures: one for the pediatric group and two for the adult groups, which were segregated by IDH mutation status (Fig. 1B; Supplementary Fig. 1B). Accordingly, pediatric glioma patients present highly conserved splicing patterns regardless of the tumor grade (LGG/HGG). Notably, adult patients diagnosed with IDH-wild-type (WT) LGG in The Cancer Genome Atlas (TCGA) database (currently considered GBM) clustered more closely with GBM patients (Fig. 1B; Supplementary Fig. 1C, D). We next assessed the expression of specific isoforms and their fractions within the entire gene expression range (all isoforms) in HGG/GBM, which presented higher expression than LGG did. We noted that 99 isoforms were expressed across the glioma spectrum, 74 of which were protein-coding isoforms attributed to 39 genes associated with the cell cycle and mitosis (Supplementary Fig. 1E–G). We further refined our results by filtering out isoforms with high expression in normal tissues to retain only those isoforms associated with HGG/GBM [32] (Supplementary Table S1). This finding was further supported by the examination of isoform expression in GBM organoids [33] (Supplementary Table S1), resulting in the selection of eight isoforms derived from seven different genes. Notably, clustering of the patient cohort on the basis of the expression of these eight isoforms mimicked the clusters obtained by whole isoform usage (Supplementary Fig. 1H). Together, these data indicate that these eight isoforms are key features associated with HGG/GBM.

Next, we assessed the expression specificity of these eight isoforms in cells within the glioma tumor microenvironment (TME) via single-cell RNA-seq (scRNA-seq). Notably, RCAN1-4 was the only isoform expressed primarily by GBM cells, with minimal to no expression in other GBM TME-associated cell types (Fig. 1C, Supplementary Fig. 1I–K). ScATAC-seq analysis revealed that, compared with other cells in the TME, GBM cells had high chromatin accessibility across the promoter sites of RCAN1 isoforms, including RCAN1-4 (Supplementary Fig. 1L, M). Analysis of scRNA-seq data for multiregional dissected gliomas [34] revealed that RCAN1-4-specific SJs were detected in tumor cells from multiple regions of both newly diagnosed and recurrent GBMs but not in LGGs (Fig. 1C; see “Methods” for more details).

Notably, in addition to RCAN1-4, the canonical RCAN1 isoform in the brain, RCAN1-1 [35], is also expressed in glioma patients. Both RCAN1-4 and RCAN1-1 share exons 5, 6, and 7 of the RCAN1 gene but differ in their first exons. Whereas RCAN1-1 expresses exon 1 as the first exon, RCAN1-4 instead uses exon 4 (Fig. 1D). In higher-grade gliomas (i.e., GBM, IDH-WT, and pediatric HGG), RCAN1-4 was the predominant RCAN1 isoform, with its expression level increasing with tumor grade (Fig. 1E, F, Supplementary Fig. 2A, B). Additionally, high RCAN1-4 expression was noted in adult gliomas with malignant molecular characteristics (Supplementary Fig. 2C) and was associated with the induction of specific mutations in GBM organoids, such as CDKN2A/B codeletion and EGFRvIII (Supplementary Fig. 2D). The progression association was further reinforced by a significant increase in RCAN1-4 expression at recurrence (Supplementary Fig. 2E).

Finally, we assessed the expression of these distinct RCAN1 isoforms across tumor types and molecular subtypes in adult and pediatric patients and normal tissues. Notably, RCAN1-4 expression was highest in GBM relative to other tumors (Supplementary Fig. 2F) and was further elevated in the mesenchymal subtype (Fig. 1G). The expression of RCAN1-4 was also significantly greater in brain metastases than in normal brain tissues, and RCAN1-4 expression was consistently low (Supplementary Fig. 2G). Furthermore, Western blot analysis of normal human tissue lysates revealed minimal RCAN1-4 expression in normal whole-tissue lysates (Supplementary Fig. 2H), supporting the utility of RCAN1-4 as a putative TAA for immunotherapy. Notably, mesenchymal subtypes usually exhibit more aggressive characteristics than other GBM subtypes do [36]; therefore, we postulated that RCAN1-4 expression may be related to the aggressiveness of GBM, such as the mesenchymal subtypes.

### C/EBPβ regulates RCAN1-4 expression in GBM cells

**Fig. 2:**
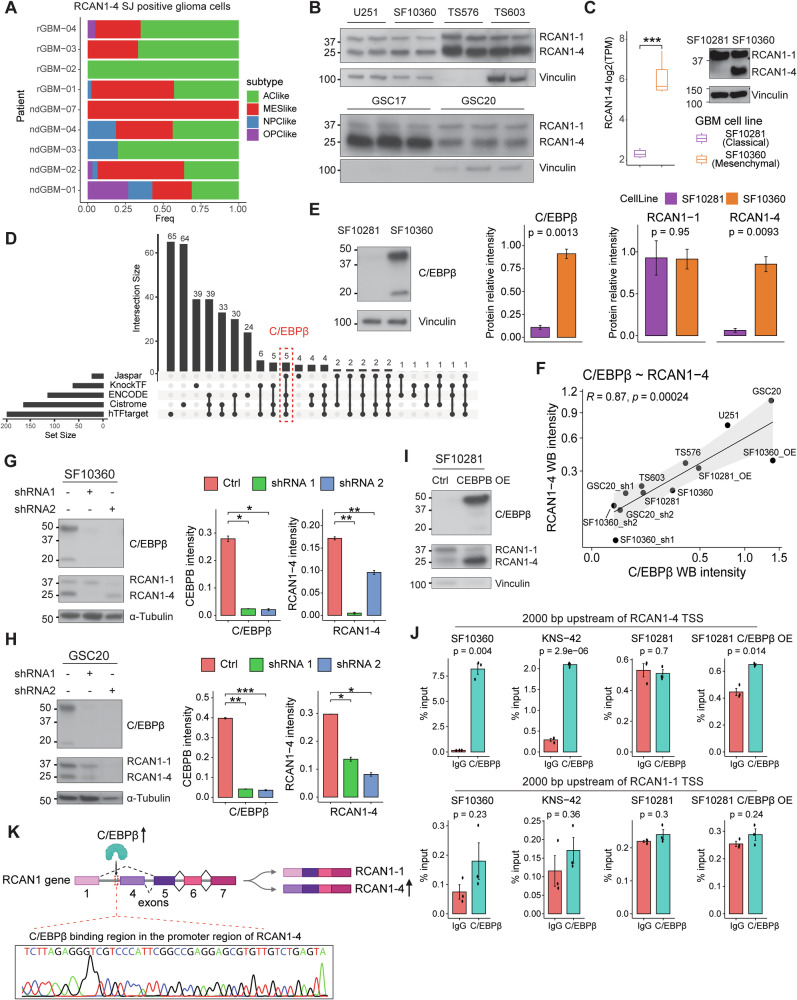
RCAN1-4 expression is regulated by C/EBPβ in mesenchymal glioma. **A** Proportion of RCAN1-4-positive tumor cells in adult GBM patients according to single-cell subtype. ndGBM, newly diagnosed GBM; rGBM, recurrent GBM. **B** RCAN1-4 expression in multiple mesenchymal GBM and GSC cell lines at the protein level. **C** RCAN1-4 expression comparison of classical (SF10281) and mesenchymal (SF10360) patient-derived GBM primary cell lines at the RNA (top left) and protein levels (top right). The statistics of protein expression performed for both isoform proteins (bottom). **D** RCAN1-4 promoter region binding transcription factor (TF) prediction. C/EBPβ was selected by excluding TFs predicted to bind to the RCAN1-1 promoter region. **E** C/EBPβ protein expression in GBM cell lines (classical vs. mesenchymal). **F** Integrated analysis of RCAN1-4 and C/EBPβ protein correlation. RCAN1-4 protein expression changes by knocking out C/EBPβ in mesenchymal GBM (**G**) and GSC (**H**) cell lines. **I** RCAN1-4 protein expression changes upon C/EBPβ overexpression in the classical GBM cell line. **J** ChIP‒PCR results of C/EBPβ binding to RCAN1 isoform promoter sites in different GBM cell lines. Top, C/EBPβ-binding DNA was amplified by RCAN1-4 promoter region primers; bottom, C/EBPβ-binding DNA was amplified via RCAN1-1 promoter region primers. **K** The C/EBPβ-binding region and the corresponding DNA sequence in the RCAN1-4 promoter region. *p < 0.05; **p < 0.01; ***p < 0.001

At the single-cell level, GBM tumor cells can be distinguished into four unique phenotypes: neural progenitor-like (NPC-like), oligodendrocyte-progenitor-like (OPC-like), astrocyte-like (AC-like), and mesenchymal-like (MES-like) GBM cell phenotypes [37]. The mesenchymal GBM subtype is usually more aggressive and is mostly associated with MES-like and AC-like phenotypes at the single-cell level [37]. Thus, to further understand the heterogeneity of RCAN1-4 in GBM, we investigated RCAN1-4 expression in GBM cells classified by molecular phenotype. Consistent with our findings, we found that RCAN1-4 was expressed primarily in GBM cells with AC-like and MES-like signatures (Fig. 2A; Supplementary Fig. 3A), which are the two main cellular components of mesenchymal GBM [37]. Thus, we next assessed the protein expression of the RCAN1 isoforms and detected high RCAN1-4 expression across multiple mesenchymal GBM cell lines and glioma stem cell (GSC) lines (Fig. 2B). Additionally, we compared the expression of RCAN1-4 between GBM cell lines with a classical subtype (SF10281) and those with a mesenchymal subtype (SF10360), both of which were derived from primary untreated GBM patients [38]. We noticed that RCAN1-4 was significantly more highly expressed in the mesenchymal subtype (SF10360) than in the classical subtype (SF10281) at both the RNA and protein levels (Fig. 2C). Similarly, U87 GBM cells, which we classified into the mesenchymal subtype on the basis of their gene expression, also presented high RCAN1-4 expression (Supplementary Fig. 3B).

Considering that these RCAN1 isoforms differ in the first exon, which is selectively transcribed during AS [39], we next performed transcription factor motif prediction on the promoter region of RCAN1-4 via five different databases [40–44]. Notably, by excluding the transcription factor predicted to bind to the promoter region of RCAN1-1, C/EBPβ was the common transcription factor that was predicted to uniquely bind to the promoter region of RCAN1-4 in four of the databases (Fig. 2D). In support of these findings, we noted coexpression of CEBPB mRNA, encoding C/EBPβ, and RCAN1-4 in GBM cells, whereas RCAN1-1 was not associated with CEBPB expression (Fig. 2E; Supplementary Fig. 3C). Likewise, CEBPB was significantly upregulated in malignant gliomas, especially in the mesenchymal subtype (Supplementary Fig. 3D, E). Moreover, we observed a strong correlation between CEBPB (C/EBPβ) and RCAN1-4 expression, both at the RNA level in glioma cohorts (Supplementary Fig. 3F) and at the protein level in the GBM cells we examined (Fig. 2F; Supplementary Fig. 3G). This correlation was more pronounced in GBM than in all other tumor types analyzed (Supplementary Fig. 3H). Accordingly, we observed that RCAN1-4 expression was strikingly reduced when C/EBPβ was knocked down in primary RCAN1-4^pos^ GBM cells and GSCs (Fig. 2G, H, Supplementary Fig. 3I, J). Furthermore, C/EBPβ overexpression in RCAN1-4^neg^ GBM cells (SF10281) strikingly upregulated the protein expression of RCAN1-4 but not RCAN1-1 (Fig. 2I, Supplementary Fig. 3K). C/EBPβ-mediated upregulation of RCAN1-4 was also observed in GBM cells with high baseline RCAN1-4 expression (SF10360) (Supplementary Fig. 3L).

To assess whether C/EBPβ binds to the RCAN1-4 promoter, we performed C/EBPβ ChIP‒PCR in both adult (SF10360 and SF10281) and pediatric (KNS-42) GBM cell lines [45, 46]. The pediatric KNS-42 GBM cell line was chosen because of its intrinsically high C/EBPβ expression (Supplementary Fig. 3M). The results showed that C/EBPβ was indeed present within the RCAN1-4 promoter region of KNS-42 and SF10360 cells (Fig. 2J, Supplementary Fig. 3N, O). Owing to the low expression of C/EBPβ in SF10281 cells, amplification of the RCAN1-4 promoter region in these cells was not possible (Fig. 2I, J; Supplementary Fig. 3P). However, upon overexpression of C/EBPβ in SF10281 cells, the RCAN1-4 promoter region was detected by C/EBPβ ChIP‒PCR (Fig. 2J). We further performed Sanger sequencing of C/EBPβ ChIP‒PCR products and confirmed the presence of C/EBPβ within the RCAN1-4 promoter region (Fig. 2K). Furthermore, we identified a consistent C/EBPβ binding peak at the RCAN1-4 promoter in independent CUT&RUN data from breast cancer cells, indicating that this regulatory mechanism may be conserved in other cancers (Supplementary Fig. 4). To further identify oncogenic regulators of C/EBPβ in GBM, we performed a correlation analysis across three independent cohorts. Four oncogenes, including SPI1, CDKN1A, VAV1, and PML, three cancer-related hallmark genes (FCGR2B, PIM1, and TNFAIP3), and key cancer-related pathways, including the p53 pathway, hypoxia, glycolysis, and RAS signaling, demonstrated consistent positive correlations with C/EBPβ (Supplementary Fig. 5A, B), suggesting their role as potential determinants of C/EBPβ expression heterogeneity in GBM.

Together, these data indicate that C/EBPβ can bind to the promoter region of RCAN1-4, and we identified it as an important regulator of RCAN1-4 expression in GBM cells, primarily associated with the mesenchymal subtype.

### HLA-A24:02-restricted RCAN1-4 splicing junction epitope discovery

**Fig. 3:**
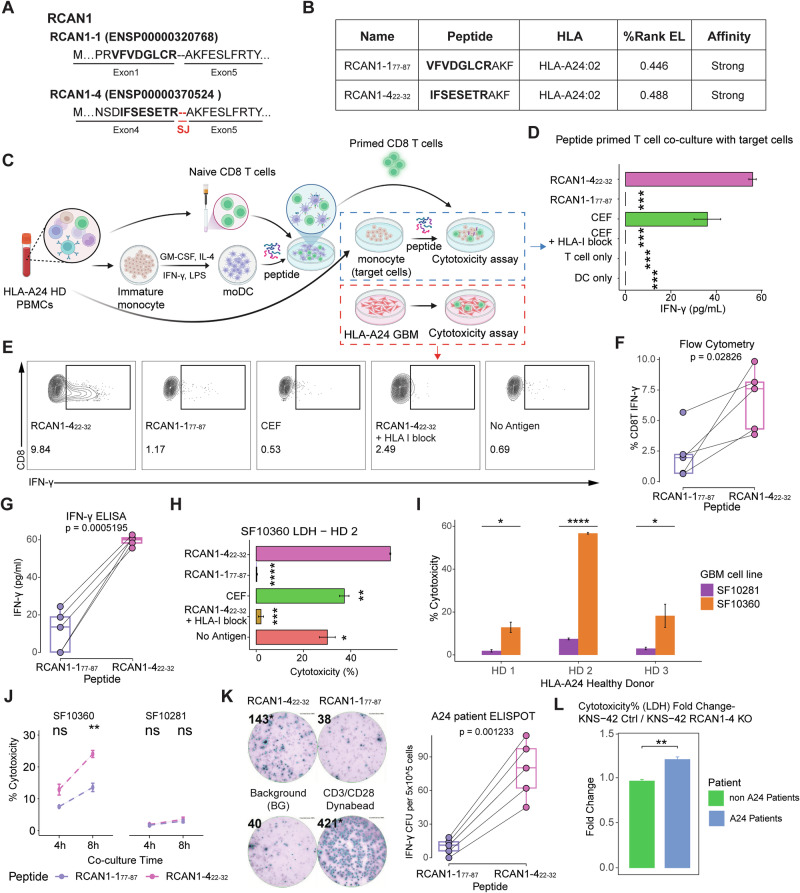
RCAN1-4 splicing junction epitopes induced T cell cytotoxicity in mesenchymal GBM. **A** Specific splicing junction (SJ) of RCAN1-4 in the protein structure. **B** Prediction of antigenic epitopes/peptides from predominant RCAN1 isoforms. **C** Schematic plot of the estimation of the immunogenicity of the selected peptide. **D** Comparison of T cell activation levels when T cells were primed with different peptides. The activation level was estimated on the basis of IFN-γ secretion from peptide-primed T cells cocultured with peptide-loaded autologous target cells. CEF, a combination of viral immunogenic peptides, was used as the positive control. **E** Flow cytometric results of peptide-primed CD8^+^ T cell intracellular IFN-γ after coculture with the SF10360 GBM cell line. These data were collected from HD4. Intracellular IFN-γ (**F**) and IFN-γ secretion (**G**) comparison of CD8^+^ T cells primed with different RCAN1 SJ peptides when cocultured with SF10360. The solid line links samples from the same healthy donor (HD; *n* = 5). The *p* value was estimated via paired *t*-tests. **H** Cytotoxic effects of peptide-primed CD8^+^ T cells on SF10360 cells. The cytotoxic effect was estimated via the LDH assay. These data were collected from HD2. **I** RCAN1-4_22-32_-primed CD8^+^ T cell cytotoxic effects on adult GBM cell lines with different transcriptomic subtypes. SF10281, classical subtype; SF10360, mesenchymal subtype. **J** RCAN1-4_22-32_-primed CD8^+^ T cell cytotoxic effects on adult GBM cell lines with different coculture times. **K** TCR clone numbers with RCAN1 SJ response in GBM patients. The number of clones was estimated via ELISPOT for IFN-γ. A paired *t*-test was conducted on HLA-A24^+^ patients (*n* = 5), with a solid line linking the same patient. The background (BG) was estimated with DMSO; the CD3/CD28 Dynabead group was used as the positive control. The colony-forming units (CFUs) were normalized by subtracting the BG. **L** Fold changes in the cytotoxic effect of expanded T cells from GBM patient PBMCs on the KNS-42 GBM cell line compared with the RCAN1-4^KO^ KNS-42 GBM cell line (*n* = 3–4/group). A24 patients, patients with the HLA-A24 allele; non-A24 patients, patients without the HLA-A24 allele. ns not significant; *p < 0.05; ***p < 0.001

The RCAN1 splice isoforms RCAN1-1 and RCAN1-4 differ in their first exons. As depicted in Figs. 1D and 2K, RCAN1-4 comprises exons 5–7 and exon 4, whereas RCAN1-1 comprises exons 5–7 and exon 1. This creates an SJ that is distinct between RCAN1-4 and RCAN1-1. Notably, unique SJs are found in various malignancies as antigenic targets capable of promoting antitumor immune responses [47, 48], including gliomas [49]. Therefore, we examined whether a unique RCAN1-4 SJ derived from the ligation between exon 4 and exon 5 (hereafter referred to as RCAN1-4 SJ_e4/e5_) could induce T cell responses (Fig. 3A).

A limiting step in the generation of cytotoxic T cell immunity is the presentation of distinct immunogenic epitopes by different MHC-I alleles [50, 51]. Therefore, we investigated the HLA-I landscape of adult and pediatric glioma cohorts to identify putative MHC-I-presenting RCAN1-4 SJ_e4/e5_ epitopes. Notably, 180 HLA-I alleles were expressed in the glioma cohorts (Supplementary Fig. 5C). Bioinformatic analysis revealed that 44.2% of these HLA-I alleles, spanning 92.4% of glioma patients, potentially presented a variety of different peptides encompassing RCAN1-4 SJ_e4/e5_ (Supplementary Fig. 5D). Furthermore, analysis of immunopeptidomics data from the RCAN1-4^pos^ GBM cell line (SF10360; Supplementary Fig. 5E) and clinical samples of GBM patients [52] revealed that RCAN1-4 SJ_e4/e5_ peptides immunoprecipitated with MHC-I molecules (Supplementary Table S2). Thus, the data suggest that peptides generated from RCAN1-4 SJ_e4/e5_ can be generated from MHC-I molecules expressed in GBM patients.

Notably, our analysis revealed that a substantial proportion of the global population possesses HLA-I alleles predicted to present RCAN1-4 SJ_e4/e5_ epitopes (Supplementary Fig. 5F). The most frequent of these was HLA-A24:02, with an estimated prevalence of ~15% in Asian and ~8% in European White populations [53]. For this prominent allele, we identified two 11-amino acid peptides: one derived from RCAN1-4 SJ_e4/e5_ and the other from the corresponding constitutive region of the RCAN1-1 isoform. Both peptides were predicted to bind strongly to the HLA-A24:02 MHC-I molecule. These peptides share a C-terminal 3-amino-acid sequence from exon 5 of the RCAN1 gene; however, they differ in amino acids 1–8. The first peptide originated from a sequence associated with RCAN1-1 (exon 1; RCAN1-1_77-87_), whereas the second peptide is associated with RCAN1-4 (exon 4; RCAN1-4_22-32_) (Fig. 3B). Importantly, searching the nonredundant protein sequence database via protein‒protein BLAST (BlastP) did not identify any region in other human proteins with meaningful sequence similarity (*E* value < 1) with the RCAN1-4_22-32 region_ (Supplementary Fig. 5G), suggesting low cross-reactivity and off-target risk [54]. Taken together, these data support RCAN1-4_22-32_ as an SJ-derived immunogenic target, meriting further investigation.

### RCAN1-4_22-32_ induces HLA-A24-dependent CD8^+^ T cell cytotoxicity against RCAN1-4^+^ GBM cells

Next, we tested the ability of RCAN1-4 SJ_e4/e5_-enriched peptides to induce HLA-A24-restricted CD8^+^ T cell responses (Fig. 3C), as previously described [55]. Notably, upon antigen recall by autologous target cells preloaded with priming peptides, CD8^+^ T cells primed with RCAN1-4_22-32_, but not those primed with RCAN1-1_77-87_, exhibited significant IFN-γ release (Fig. 3D, Supplementary Fig. 5H).

To determine whether RCAN1-4_22-32_ mediates T cell cytotoxicity against GBM, we recalled RCAN1-4_22-32_-primed T cells from different HLA-A24^+^ healthy donors (HDs) with HLA-A24^+^ GBM cells expressing RCAN1-4 at various levels (Fig. 3C; Supplementary Tables S3 and S4). Importantly, CD8^+^ T cells primed with RCAN1-4_22-32_, but not RCAN1-1_77-87_, secreted high levels of IFN-γ when cocultured with HLA-A24^+^ RCAN1-1^pos^ RCAN1-4^pos^ GBM cells (SF10360) (Fig. 3E‒G, Supplementary Fig. 6A‒C). Blocking HLA-I with anti-MHC-I antibodies prevented RCAN1-4_22-32_-primed CD8^+^ T cells from expressing IFN-γ upon rechallenge (Fig. 3E, Supplementary Fig. 6A–C), confirming that the T cell responses were HLA-I restricted. Similar findings were obtained in cocultures with HLA-A24^+^ RCAN1-1^pos^ RCAN1-4^neg^ GBM cells (SF10281), although to a lesser extent (Supplementary Fig. 6D–F), indicating that RCAN1-4_22-32_ can induce an antitumor T cell response even in the context of low RCAN1-4 expression. Correspondingly, we observed significant HLA-I-dependent tumor killing by LDH assay upon coculturing RCAN1-4_22-32_-primed CD8^+^ T cells with SF10360 (RCAN1-4^pos^ GBM cells) (Fig. 3H, Supplementary Fig. 7A, B). In agreement with these findings, RCAN1-4_22-32_-primed CD8+ T cells killed significantly fewer tumor cells when cultured with SF10281 (RCAN1-4^neg^ GBM cells than RCAN1-4^pos^ GBM cells) (Fig. 3I, Supplementary Fig. 7C, D). Furthermore, T cell antitumor cytotoxicity mediated by RCAN1-4_22-32_ increased over time (Fig. 3J).

To determine whether GBM patients exhibit preexisting T cells responsive to RCAN1-4_22-32_, we performed a cytokine ELISPOT assay using peripheral blood mononuclear cells (PBMCs) from HLA-A24^+^ GBM patients to quantify the frequencies of RCAN1-1_77-87-_ and RCAN1-4_22-32_-specific T cells in each sample. Importantly, we noted increased frequencies of IFN-γ^+^ RCAN1-4_22-32_ peptide-reactive T cells, but not RCAN1-1_77-87_-reactive T cells, which were significantly elevated in PBMCs from GBM patients (Fig. 3K). Upon normalizing the frequencies of IFN-γ^+^ RCAN1-4_22-32_ peptide-reactive T cells to the total CD3^+^ T cell population in each sample, we observed a negligible increase in their mean percentage in the PBMCs of GBM patients compared with those of healthy individuals. However, significant and profound enrichment of these cells was detected in tumor-infiltrating T cells (TILs) from the same patients (Supplementary Fig. 7E). These findings suggest a clonal expansion of RCAN1-4-reactive T cells specifically within the GBM TME, which is consistent with previously published reports [56, 57]. Compared with wild-type KNS-42 cells, RCAN1-4 knockout (KO) HLA-A24:02^+^ KNS-42 GBM cells presented greater proliferative and survival capacities (Supplementary Fig. 7F–I). In coculture assays with T cells from patient PBMCs, T cells from HLA-A24^+^ patients exhibited increased tumor cell killing ability when cultured with RCAN1-4^pos^ KNS-42 cells compared with RCAN1-4^KO^ KNS-42 cells (fold change >1) (Fig. 3L). T cells from HLA-A24^−^ patients killed similar numbers of tumor cells when cultured with RCAN1-4^pos^ KNS-42 cells or RCAN1-4^KO^ KNS-42 cells (fold change = 1) (Fig. 3L; Supplementary Fig. 7J).

Taken together, our data show that RCAN1-4_22-32_ is an immunogenic SJ epitope that promotes antigen-specific CD8^+^ T cell immunity against GBM in an HLA-A24-restricted manner.

### Identification and phenotypic characterization of RCAN1-4_22-32_-specific HLA-A24:02-restricted T cell clones

**Fig. 4:**
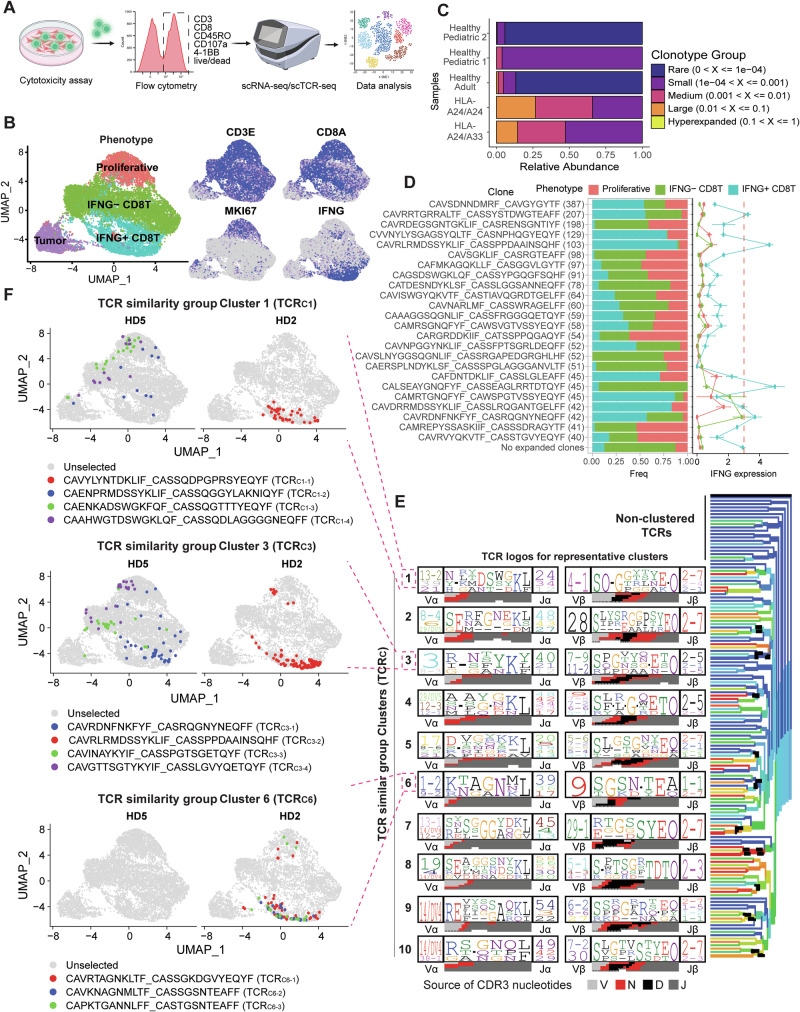
Identification of TCR clones that respond to RCAN1-4_22-32_. **A** Schematic representation of single-cell sequencing of RCAN1-4_22-32_-recognized T cell clones. **B** UMAP of single cells collected via flow cytometry. **C** T cell clonotype expansion in different samples. **D** TCR CDR3 region of T cells with clonal expansion. The expanded T cell number is listed next to each CDR3 sequence. The subtypes of each clonotype are shown in Fig. 4B. The average IFN-γ expression in the three clusters is shown next to each clonotype. **E** TCR clonotype cluster based on the TCR CDR3 structure. **F** UMAP projection of clonotypes of TCR-similarity groups 1, 3, and 6 that are shown in Fig. 4E. HD5, healthy donor with a homozygous HLA-A24 allele; HD2, healthy donor with HLA-A24 and HLA-A33 alleles

Subsequently, RCAN1-4_22-32_-reactive TCRs were identified, cloned, and engineered into TCR-T cells to validate their cytotoxic efficacy (Supplementary Fig. 8A). We primed naive CD8^+^ T cells from two HLA-A24^+^ HDs separately with RCAN1-4_22-32_ in vitro, followed by recall with RCAN1-4^pos^ SF10360 GBM cells (Fig. 4A, Supplementary Fig. 8B). To enrich for tumor-reactive T cells, T cells were sorted by FACS on the basis of the expression of cell surface markers indicating antigen-experienced T cells, which was confirmed by scRNA-seq/scTCR-seq (Fig. 4A, B, Supplementary Fig. 8C, D). We observed striking T cell clonal expansion (>50%) within RCAN1-4_22-32_-primed CD8^+^ T cells, whereas CD8^+^ T cells that did not experience peptide priming presented with a diverse and nonexpanded TCR repertoire (Fig. 4C; Supplementary Fig. 8E, F). Compared with CD8^+^ T cells from other healthy donors, the majority of RCAN1-4_22-32_-primed CD8^+^ T cells exhibited a progenitor exhausted (TPEX) phenotype, and only a very small subset of cells presented a naive-like T cell phenotype (Supplementary Fig. 8G, H). Among the collected TCR clones, a few public TCRs were identified as virus-specific (Supplementary Fig. 8I). Thus, these data suggest that stimulation with the RCAN1-4_22-32_ peptide resulted in the expansion and differentiation of antigen-specific CD8^+^ T cell clones.

IFN-γ production and clonal expansion are useful markers indicative of RCAN1-4_22-32_-mediated antitumor T cell immunity [58, 59]. However, this approach might miss putative RCAN1-4_22-32_-reactive TCRs with different functional properties. Accordingly, we performed scRNA-seq analysis of clonally expanded TCRs. As shown in Fig. 4B, UMAP analysis of RCAN1-4_22-32_-primed CD8^+^ T cells revealed three distinct phenotypes: proliferative CD8^+^ T cells (red; high MKI67), IFNG^neg^ CD8^+^ T cells (green), and IFNG^pos^ CD8^+^ T cells (blue) (Fig. 4B; Supplementary Fig. 8J). The phenotypes of the expanded T cell clones (*n* > 40 cells/clone) were characterized by assessing the proportion of each phenotype and the expression of cytotoxic and activation markers (Fig. 4D; Supplementary Fig. 9). While each clone exhibited a dominant phenotype (IFNG^pos^, IFNG^neg^, or proliferative), all three phenotypes were present within each clone (Fig. 4D). Notably, not all expanded clones were predominantly composed of IFNG^pos^ CD8^+^ T cells or displayed high expression of IFNG or other cytotoxic markers (Fig. 4D; Supplementary Fig. 9). Therefore, we incorporated additional criteria for the selection of putative RCAN1-4_22-32_-reactive TCRs.

Accordingly, we performed TCR similarity group clustering on the basis of CDR3 sequence homology as an indicator of antigen specificity [60, 61]. We identified ten TCR similar group clusters (TCR_C_) with similar V(D)J segment recombination patterns and CDR3 homology (Fig. 4E). By projecting the TCRs within each cluster onto the UMAP-annotated phenotypes, we obtained three TCR_Cs_ containing expanded TCRs with high IFNG expression. These TCR_Cs_ included cluster-1 (TCR_C1_), cluster-3 (TCR_C3_), and cluster-6 (TCR_C6_) (Fig. 4F). Notably, both donors presented TCR_C1_ and TCR_C3_ TCR clonotypes, with individual clones displaying distinct phenotypes. For example, TCR_C3_ consists of four unique TCR clonotypes: clonotype 1 (TCR_C3-1_), clonotype 3 (TCR_C3-3_), and clonotype 4 (TCR_C3-4_), which originate from one donor (HD5), whereas clonotype 2 (TCR_C3-2_) is derived from the second donor (HD2). Despite sharing similar TCR CDR3 sequences, the clones presented different functional characteristics. Clones 1 (TCR_C3-1_) and 2 (TCR_C3-2_) were associated with high IFNG expression. In contrast, clone 3 (TCR_C3-3_) showed an IFNG-negative phenotype, and clone 4 (TCR_C3-4_) was associated primarily with a proliferative CD8^+^ T cell phenotype. Similar observations were made in the TCR_C1_ group. These findings highlight that even within a TCR similarity group with presumed shared antigen specificity, subtle variations in the TCR CDR3 sequence can significantly impact T cell function, as previously reported [62]. In contrast to TCR_C1_ and TCR_C3_, TCR_C6_ was composed of three distinct expanded clones derived from the same donor and exhibited an IFNG^+^ phenotype.

Taken together, these results suggest that TCR_C1_, TCR_C3_, and TCR_C6_ likely encompass RCAN1-4_22-32_-specific TCRs capable of inducing an antitumor immune response.

### TCR-transduced RCAN1-4_22-32_-reactive T cells efficiently kill GBM cells in vitro

**Fig. 5:**
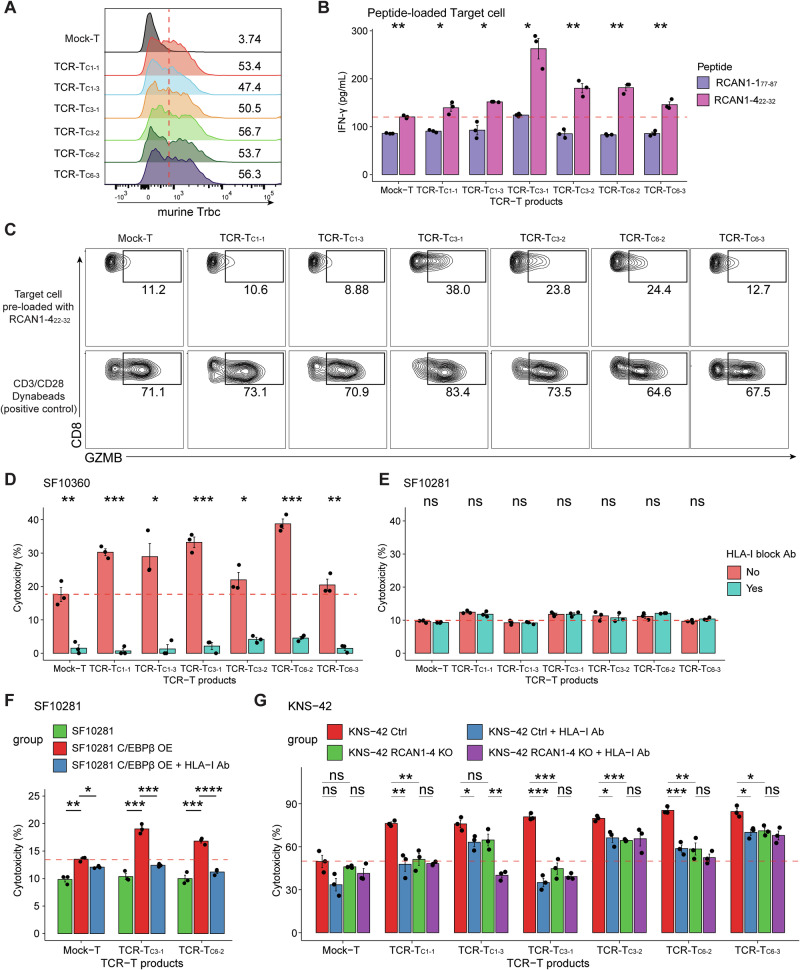
In vitro functional estimation of RCAN1-4_22-32_-targeted TCR-T cells. **A** Transduction efficiency of TCR-T cells. The efficiency of the method was evaluated by flow cytometry, which targeted the murine TCR β chain constant region designed for the transduced TCR. **B** TCR-T IFN-γ secretion levels in cells cocultured with RCAN1 peptide-loaded autologous target cells. **C** TCR-T intracellular GZMB expression in cells cocultured with RCAN1-4_22-32_-loaded autologous target cells. **D**, **E** TCR-T cell cytotoxicity effects on different HLA-A24:02^+^ patient-derived primary GBM cell lines, SF10360 (mesenchymal subtype, Fig. 5D), and SF10281 (classical subtype, Fig. 5E). Statistical significance was estimated between groups with/without anti-human MHC-I antibody in each TCR-T. **F** TCR-T cytotoxicity effects on C/EBPβ-overexpressing SF10281 cells. **G** TCR-T cell cytotoxicity effects on the HLA-A24:02^+^ pediatric GBM (KNS-42) and RCAN1-4^KO^ KNS-42 cell lines. HLA-I Ab, anti-human MHC-I antibody. Mock-T, T cells transduced with empty virus. ns not significant; *p < 0.05; **p < 0.01; ***p < 0.001; ****p < 0.0001

On the basis of our observations, we selected two TCRs from each cluster for TCR-T cell construction and further analysis: TCR_C1-1_, TCR_C1-3_, TCR_C3-1_, TCR_C3-2_, TCR_C6-2_, and TCR_C6-3_. To construct TCR-T cells with selected TCR sequences, we isolated CD8+ T cells from the PBMCs of HLA-A24^+^ HDs (HD5; Supplementary Fig. 10A) and performed lentiviral transduction [63]. We designed a hybrid TCR lentiviral plasmid vector by combining selected paired human TCR V(D)J sequences with the murine TCR constant region of the alpha and beta chains [63]. The TCR-T cell transduction efficiency was similar across TCR-T cell types (Fig. 5A; Supplementary Fig. 10B). To assess the cytotoxic potential of TCR-T cells, they were cultured with RCAN1-4_22-32_- or RCAN1-1_77-87_-loaded autologous target cells. Notably, all the TCR-T products exhibited significantly greater cytotoxicity against autologous targets preloaded with RCAN1-4_22-32_ than against those loaded with RCAN1-1_77-87_ (Fig. 5B; Supplementary Fig. 10C). The level of activation varied among the TCR-T cells, with TCR_C3-1_ cells presenting the highest IFN-γ and granzyme B (GZMB) expression, followed by the TCR_C6-2_ and TCR_C3-2_ TCR-T cells (Fig. 5B, C). Similar to their ability to produce cytokines upon antigen stimulation, TCR-T cells exhibited significant HLA-dependent killing of RCAN1-4^pos^ GBM (SF10360) but not RCAN1-4^neg^ GBM (SF10281) cells (Fig. 5D, E and Supplementary Fig. 10D). Remarkably, TCR-T cells exhibited sustained cytotoxicity against GBM cells over a 7-day coculture period (Supplementary Fig. 10E), and prolonged exposure to TCR-T cells did not downregulate RCAN1-4 expression on the target tumor cells, suggesting that the antigen is not susceptible to loss under this therapeutic pressure in vitro (Supplementary Fig. 10F).

Since C/EBPβ regulates RCAN1-4 expression in GBM cells (Fig. 2J), we tested whether the overexpression of C/EBPβ in RCAN1-4^neg^ GBM cells (SF10281) induces RCAN1-4_22-32_-reactive TCR-T cytotoxicity. Strikingly, the HLA-dependent cytotoxicity of TCR-T cells was significantly increased in the SF10281 GBM line with induced C/EBPβ overexpression (SF10281^C/EBPβ/RCAN1-4 pos^) (Fig. 5F). Adding an anti-human MHC-I antibody inhibited killing and resulted in a similar tumor cytotoxicity ratio between SF10281 and SF10281^C/EBPβ/RCAN1-4 pos^. Thus, these data suggested that C/EBPβ upregulation in GBM cells could invoke RCAN1-4-dependent antitumor T cell immunity. Notably, silencing RCAN1-4 expression in RCAN1-4^pos^ GBM cells significantly impeded tumor killing by TCR-T cells, which was comparable to the inhibition observed after antibody-mediated blockade of MHC-I (Fig. 5G; Supplementary Fig. 10G, H). The results were confirmed with CD8^+^ T cells from another HLA-A24^+^ HD (HD6), yielding similar results (Supplementary Fig. 11A–G).

We performed comparative transcriptomics analysis between cells with strong reactivity to the RCAN1-4 peptide (high IFN-γ expression) and T cells with low/no RCAN1-4 reactivity (low/no IFN-γ expression). We evaluated the relative expression of genes associated with the phenotype of cancer-reactive T cells [64–72] in these two groups. We noted that T cells with high reactivity toward RCAN1-4 presented significantly greater expression of many tumor-reactive T cell-related genes, most notably CCL20 [65], SDC4 [71], and SPRY1 [64], with greater than twofold greater expression than T cells with low/no response to RCAN1-4 (Supplementary Fig. 11H).

To model the potential immunosuppressive microenvironment of GBM encountered by transferred TCR-T cells, we cultured TCR-T cells with the immunosuppressive cytokines IL-10 and TGF-β in vitro. This assay tested whether TCR-T cells could maintain persistence and cytotoxic function under immunosuppressive conditions. In this cytokine milieu, TCR-T cell viability remained unaffected (Supplementary Fig. 12A), and the expression of the exhaustion markers PD-1 and TIM-3 was not significantly increased compared with that in mock-T cells or TCR-T cells cultured without immunosuppressive cytokines (Supplementary Fig. 12B, C). To further investigate potential functional impairment by tumor-secreted factors, we cultured TCR-T cells in conditioned tumor media. Similarly, we observed no significant increase in exhaustion markers compared with those in mock-T cells or TCR-T cells cultured in normal media (Supplementary Fig. 12B, C). These results demonstrate the functional resilience of TCR-T cells in both cytokine-induced and tumor-derived immunosuppressive environments.

### Efficacy of TCR-transduced RCAN1-4_22-32_-reactive T cells in vivo

**Fig. 6:**
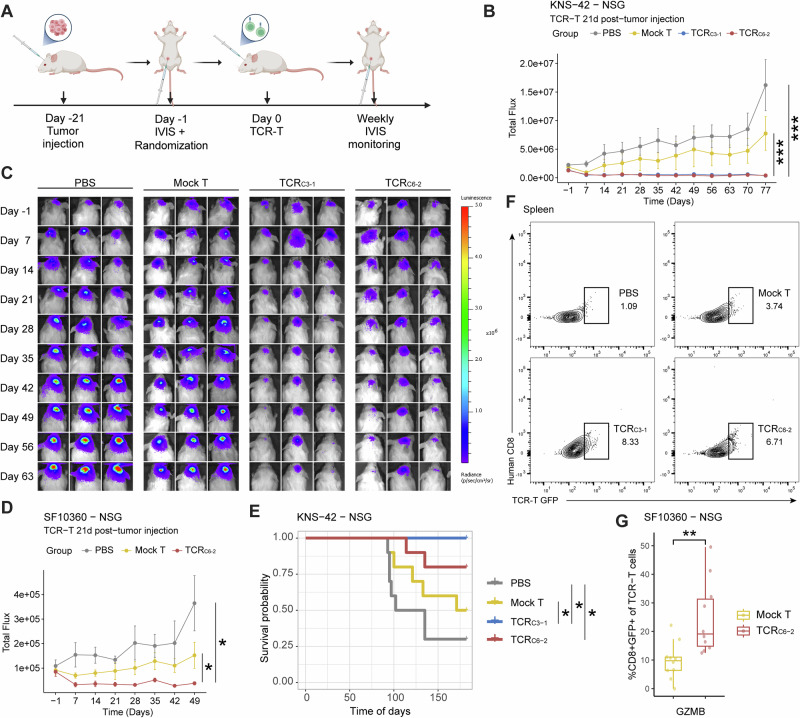
In vivo functional estimation of RCAN1-4_22-32_-targeted TCR-T cells. **A** Schematic of the in vivo study design. The mice were randomized on the basis of their bioluminescence signal (IVIS) one day before TCR-T cell transfer. **B**, **C** Weekly alteration in the bioluminescence imaging signal in a KNS-42 (pediatric GBM) xenograft murine model after TCR-T cell transfer. **D** Weekly alteration of the bioluminescence imaging signal in the SF10360 (adult GBM) xenograft murine model after TCR-T cell transfer. *p < 0.05; **p < 0.01; ***p < 0.001. **E** Survival data of the KNS-42 xenograft murine model; *p < 0.05. **F** Flow cytometry results of TCR-T cells in the mouse spleen 120 days after TCR-T transduction. For TCR-T-GFP, GFP was encoded in the plasmid and transduced with lentivirus as a reporter marker during TCR-T cell construction. **G** Assessment of granzyme B expression in TCR-T cells isolated from the spleens of SF10360 xenograft mice 150 days after TCR-T cell transfer. T cells were stimulated with PMA-ionomycin prior to analysis; **p < 0.01

Having demonstrated specific recognition of the RCAN1-4_22-32_ epitope and tumor cell lysis of RCAN1-4^pos^ human GBM cells by TCR_C3-1_- and TCR_C6-2_-engineered human T cells (TCR-T_C3-1_ and TCR-T_C6-2_, respectively), we next aimed to assess their therapeutic potential in vivo. We established orthotopic xenograft models of adult and pediatric RCAN1-4^pos^ GBM cells modified to express luciferase. Immunodeficient NSG mice were intracranially injected with luciferase-expressing SF10360 (adult) or KNS-42 (pediatric) RCAN1-4^pos^ GBM cells. Prior to treatment (day -1), the tumor burdens of the mice were evaluated, and those with similar signals were randomized into treatment groups (*n* = 10/group; two independent experiments) to ensure uniformity. The mice were treated with PBS or mock-T, TCR-T_C3-1_, or TCR-T_C6-2 cells_ via intracranial tumor injection starting on day 21 after tumor inoculation, and the tumor burden was monitored weekly via bioluminescent imaging. (Fig. 6A).

As expected, mice implanted with RCAN1-4^pos^ GBM cells exhibited continued tumor growth when receiving control treatment (mock-T cells or PBS). In contrast, injection of either TCR-T_C3-1_ or TCR-T_C6-2_ cells resulted in a marked reduction in tumor burden or stable disease, with a minimal increase in tumor size (Fig. 6B–D; Supplementary Fig. 13A). Consistent with our in vitro findings, the transfer of mock-treated T cells resulted in a moderately smaller tumor burden than did PBS treatment, suggesting that RCAN1-4-independent antitumor T cell immunity is involved, which is in line with the published literature [73, 74]. Furthermore, we observed prolonged survival in KNS-42 GBM-bearing mice treated with RCAN1-4_22-32_-reactive TCR-T cells compared with mock-treated or PBS-treated mice (Fig. 6E).

To assess the in vivo degranulation and function of RCAN1-4-specific TCR-T cells, we analyzed tumor-infiltrating lymphocytes by flow cytometry on days 3 and 15 after transfer. Compared with mock-T cells, RCAN1-4-specific TCR-T cells exhibited stable viability over time (Supplementary Fig. 13B) and significantly elevated CD107a expression (Supplementary Fig. 13C), indicating sustained survival and cytotoxic function within the TME. Compared with mock-T cells, TCR-T_C3-1_ and TCR-T_C6-2_ cells (GFP⁺) remained detectable at increased frequencies in the spleen 120 days after transfer (Fig. 6F), demonstrating durable engraftment. Moreover, upon ex vivo PMA-ionomycin rechallenge, these persistent TCR-T cells produced significantly higher levels of granzyme B (GZMB) than mock-T cells did (Fig. 6G), confirming their long-term functional competence under chronic immunosuppression. Together, these findings establish that TCR-T cells achieve sustained antitumor persistence and functionality in vivo. To evaluate systemic toxicity following adoptive transfer of RCAN1-4_22-32_-targeted TCR-T cells, we harvested major organs from four mice per treatment group (PBS, mock-T, TCR-T_C3-1_, and TCR-T_C6-2_) at the study endpoint. Comprehensive histopathological analysis revealed no evidence of on-target, off-tumor toxicity in any of the examined tissues (Supplementary Table 5).

Taken together, these data show that computationally predicted RCAN1-4_22-32_-reactive TCR-T cells from similar TCR groups can generate RCAN1-4_22-32_-MHC-dependent cytotoxicity in vitro and in vivo and reveal RCAN1-4 SJ_e4/e5_ as a novel immunogenic target for immunotherapy in GBM.

## Discussion

In gliomas, aberrant splicing events accumulate during malignancy progression, resulting in a potential pool of antigenic targets for immunotherapy [16, 75]. Mesenchymal GBM is the most aggressive molecular subtype of GBM [36]. Interestingly, the expression of C/EBPβ is a hallmark of mesenchymal GBM [4], where it drives the mesenchymal transformation of GBM and enhances tumor invasiveness [76]. While RCAN1 is a marker for MES-like GBM cells [37], its functional role in tumor progression remains conflicting, with reported effects ranging from tumor suppression [77] to the promotion of proliferation and invasion [78]. Here, we demonstrated that C/EBPβ regulates the expression of alternative splice isoforms within RCAN1, explaining the concordance between C/EBPβ and RCAN1 in mesenchymal GBM. We showed that RCAN1-4 was the predominant isoform associated with glioma aggressiveness and the most abundant RCAN1 transcript in GBM. The expression of the canonical RCAN1-1 isoform was low in both GBM and LGG, suggesting that the function of RCAN1 may be mostly attributed to RCAN1-4. The transcription factor C/EBPβ promotes the expression of RCAN1-4 in mesenchymal GBM and stem cells (GSCs) by binding to a TSS near exon 4. The overexpression of C/EBPβ increases RCAN1-4 levels, whereas its silencing decreases them. Mechanistically, C/EBPβ activates RCAN1-4 through direct promoter binding and can operate via both calcineurin/NFAT-dependent and calcineurin-independent pathways [79]. Additionally, C/EBPβ can recruit epigenetic coactivators such as p300 to facilitate chromatin remodeling, which supports transcription [80]. This model is supported by studies showing increased promoter occupancy in disease models and conserved epigenetic mechanisms [80]. Therefore, the precise role of C/EBPβ-mediated regulation in RCAN1-4 splicing is likely multifaceted and should be further elucidated in future work. In light of our findings, we postulate that future work should directly assess the cellular interplay between C/EBPβ and RCAN1-4 and their effects on the cellular functions of tumor cells.

An unresolved question concerns the function of RCAN1-4 in GBM. RCAN1-4 functions as a tumor suppressor in several malignancies [81–86]. However, our results indicated that RCAN1-4 expression was most strikingly increased in aggressive GBM and tended to increase with tumor grade. Moreover, we found that adult and pediatric GBM, which are distinct tumor types, both expressed RCAN1-4. Thus, although RCAN1-4 may have a tumor suppressor function, its role in tumor progression remains incompletely understood, and it may contribute to GBM progression.

Immunotherapy aimed at simultaneously targeting multiple tumor antigens has shown favorable results for various malignancies [87, 88], including GBM [21, 89]. We propose that targeting RCAN1-4 SJ peptides could provide immunogenic antigens to improve the efficacy of immunotherapy when combined with other known antigens. Importantly, a hallmark of therapy resistance in recurrent GBM is a shift toward mesenchymal GBM characteristics [90–92]. Additionally, GSCs remain quiescent during treatment and respond poorly to chemoradiotherapy [16, 93]. Thus, RCAN1-4 could serve as a targetable tumor antigen for therapy-resistant GBM and GSCs. Furthermore, the number of tumor antigens currently targeted for immunotherapy in GBM is limited, and these antigens are designed primarily for HLA-A2^+^ patients [94], restricting their applicability in non-HLA-A2^+^ patients. Our previous findings revealed that HLA-A24:02 is prevalent both in the general population and among patients with gliomas [53], which is consistent with prior epidemiological studies [95]. Here, we identified RCAN1-4_22-32_ as a novel immunogenic target, specifically for HLA-A24^+^ mesenchymal GBM patients. This discovery provides an additional immunotherapeutic approach for GBM patients expressing this common HLA allele [53].

An immunogenic antigen requires an adequate expression level in cells [96]. Furthermore, the selective expression of an antigen by tumor cells helps to avoid off-target effects of immunotherapy. Thus, our observation of low to absent expression of RCAN1-4 in normal tissues supports its utility, which is also supported by the reported low expression of RCAN1-4 by myeloid cells during inflammation [97] and our findings of its expression by activated myeloid/microglia and MDSCs in the GBM TME. Thus, RCAN1-4 is a TAA target, similar to other overexpressed TAAs. Given that RCAN1-4 expression is above the speculated expression threshold of immunogenic antigens [98] in GBM but not in normal tissues, we posit that targeting RCAN1-4 is less likely to induce autoimmune self-reactive T cell responses in the healthy brain. Notably, several TAAs currently evaluated in clinical trials, such as EphA2 [99] and Survivin [100] for GBM and MUC1 for colorectal adenoma [101], are highly expressed in some normal tissues at the mRNA level without evidence for the induction of autoimmune responses. Nonetheless, we suggest that targeting the RCAN1-4 antigen via adoptive cell therapy approaches could be improved by incorporating genetic safety switches [102] or additional modifications to engineered T cell products, such as the synNotch system in CAR-T cells [103].

A key finding of our in vivo study was the dissociation between terminal bioluminescence imaging (BLI) measurements and long-term survival outcomes in mice treated with the two RCAN1-4_22-32_-reactive TCR-T products. Despite comparable tumor bioluminescence and survival at the final imaging timepoint (day 77), a subsequent divergence in survival, marked by late deaths in the TCR-T_C6-2_ group, suggests that BLI failed to capture a critical difference in the minimal residual disease burden between groups. This discrepancy aligns with well-established limitations of BLI, including its inadequate sensitivity for detecting infiltrative micrometastases and its susceptibility to signal attenuation in deep tissues, which precludes accurate absolute quantification of the tumor burden [104]. Therefore, while BLI is useful for tracking gross tumor dynamics, our data reinforce that overall survival remains the definitive and most reliable endpoint for evaluating the durable efficacy of T cell therapies in preclinical models.

Finally, the intratumoral delivery of TCR-T cells may enhance therapeutic efficacy by circumventing the blood‒brain barrier [105]. Preclinical studies have reported improved antitumor efficacy of adoptive cell therapy against brain tumors via intracranial tumor injection versus intravenous administration [106, 107]. This strategy is currently being tested in clinical trials for the treatment of GBM, and encouraging results have been reported [108, 109]. Similarly, in the current work, we tested the intracranial tumor injection of RCAN1-4-targeted TCR-T cells and demonstrated its efficacy. We postulated that this approach may minimize the off-target effects of RCAN1-4-targeted TCR-T cells while maximizing tumor penetration of the TCR-T cells.

In this study, we identified RCAN1-4 as a promising target in mesenchymal GBM. However, a direct in vivo comparison of TCR-T efficacy between mesenchymal (e.g., SF10360, KNS-42) and classical (SF10281) subtypes is needed to confirm its subtype-specific therapeutic potential. Our in vitro data revealed a clear correlation between TCR-T cell cytotoxicity and RCAN1-4 expression. TCR-T cells had minimal activity against low-expressing classical SF10281 cells, but their efficacy was restored when C/EBPβ overexpression upregulated RCAN1-4, suggesting that the efficacy is mediated by the C/EBPβ–RCAN1-4 axis. Future studies with orthotopic models containing both subtypes should focus on directly comparing TCR-T efficacy and tumor control prior to clinical translation.

Our findings are subject to two important constraints. MHC restriction (HLA-A24) currently precludes obtaining HLA-matched normal human cells for coculture studies, leaving potential on-target, off-tumor toxicity incompletely characterized. Moreover, our functional analyses were conducted in animal models, which may not fully recapitulate the protracted biological pressures and evolutionary timeline of human GBM, potentially failing to reveal antigen loss or other immune escape mechanisms that could emerge during long-term clinical treatment. Consequently, clinical advancements in this therapy necessitate phased dose-escalation trials with proactive monitoring for antigen loss and other resistance variants.

In summary, we showed that mesenchymal GBM/HGG cells express high levels of RCAN1-4 in a C/EBPβ-dependent manner and contain an immunogenic epitope for the HLA-A24:02 allele. We developed RCAN1-4-targeting TCR-T clones that effectively eradicated or stabilized GBM tumors in vitro and in vivo. Overall, we revealed that RCAN1-4 is a novel TAA for malignant glioma immunotherapy in adult and pediatric patients. Our data provide new insights into the antigenic landscape of GBM and a feasible approach to improve immunotherapy via the use of RCAN1-4-targeted TCR-Ts in patients with GBM.

## Methods

### Data download and preprocessing

Bulk RNA-seq data and metadata of 156 adult GBM samples (144 primary and 12 recurrent) and 431 adult LGG samples (417 primary and 14 recurrent) were downloaded from TCGA database (https://www.cancer.gov/tcga/). Pediatric HGG (73 initial, 33 progressive, and 12 recurrent) and LGG (191 initial, 34 progressive, and 11 recurrent) bulk RNA-seq data and metadata were acquired from the Children’s Brain Tumor Tissue Consortium (CBTTC) database (https://cbtn.org/). The tumor types and diagnoses followed the records in the databases. Bulk RNA-seq data from paired primary and recurrent GBM samples were obtained from the European Genome-phenome Archive (EGA; https://www.ebi.ac.uk/ega/home) under accession number EGAS00001004524 [16]. An independent validation cohort comprising 131 glioma samples (42 LGG and 89 GBM) obtained from Xiangya Hospital, Central South University, was established. All diagnoses were histopathologically confirmed by a neuropathologist prior to bulk RNA sequencing. Bulk RNA-seq data from brain metastasis samples were acquired from public bioprojects of the National Library of Medicine database. A cohort with annotated primary origins included lung cancer (LUCA, *n* = 16), breast cancer (BRCA, *n* = 23), and kidney renal clear cell carcinoma (KIRC, *n* = 7) samples, curated from PRJNA594359 [110], PRJNA681304 [111], PRJNA790637 [112], and PRJNA454372 [113], and an additional unannotated cohort (PRJNA689295 [114]) without specified primary origins was also included. In reference to the human GRCh38 reference (release 100), we used Salmon [115] to calculate the isoform/transcript count matrix and normalized it by transcripts per million (TPM). The bulk RNA-seq isoform expression data and metadata of the brain cortex (*n* = 105) and other normal tissues were acquired from the GTEx database via UCSC Xena (https://xenabrowser.net/) [116]. Specifically, pancancer expression data for RCAN1-4 were acquired from the TCGA database via the same platform. Gene expression data were obtained by aggregating the expression of isoforms/transcripts from the same genes, and the PSI index was estimated via SUPPA2 [117] with reference to the GRCh38 GTF file. PSI features without any expression in glioma cohorts were removed for further analysis. Pediatric HGG single-cell ATAC-seq (scATAC-seq) data (GSE163655) [118] and adult GBM scATAC-seq data with paired single-nuclei RNA-seq (snRNA-seq) data (GSE174554) [119] were downloaded from the GEO database (https://www.ncbi.nlm.nih.gov/geo/). GBM single-cell Smart-seq2 data and metadata (SRP079058) [120], 10X glioma multiregional scRNA-seq data (2 LGG, 11 newly diagnosed GBM, and 5 recurrent GBM) (SRP332536) [34], GBM organoid bulk RNA-seq data and metadata (SRP132872) [33], and healthy pediatric PBMC 10X single-cell data (GEX + TCR) (SRP337596) [121] were downloaded from the Sequence Read Archive (https://www.ncbi.nlm.nih.gov/sra/). Immunopeptidomic data from glioma patients were acquired from previous studies [52, 122]. The isoform-corresponding protein sequence was downloaded from the Ensembl database (https://useast.ensembl.org/) [123]. PBMC 10X single-cell data (GEX and TCR) of healthy adults were downloaded from the 10X Genomics dataset (Demultiplexing 5′ Immune Profiling Libraries Pooled with Hashtags—10x Genomics). Peptide homologs were searched via BLASTP [124]. HLA-I allele frequency data across four major populations—European descent (EUR), Asian/Pacific Islander (API), African American (AFA), and Hispanic/Latino (HIS) populations—were sourced from the National Marrow Donor Program (NMDP) database (*n* = 1,242,890 individuals) [125].

### PSI consensus clustering

The comparison to obtain switched/shifted isoforms in glioma was conducted via the IsoformSwitchAnalyzeR R package [126]. Isoforms with different isoform fractions > 0.1 and a Benjamin–Hochberg adjusted false discovery rate (FDR) < 0.05 were considered switched isoforms. The coding potential of the isoform was predicted via CPAT [127]. We then excluded AS events (from the SUPPA2 reference) unrelated to the switched isoforms and used the PSI of the selected AS events as the input for consensus clustering via the ConsensusClusteringPlus R package (*k* = 2–10) [128].

### Malignancy-related isoform filtration

The switched isoforms were further filtered by pancancer analysis via GEPIA2 [32] and by their expression in GBM organoids compared with that in normal controls via the Wilcoxon rank-sum test. The isoforms that were highly expressed in tumors, including GBM and GBM organoids, were identified as malignancy-related isoforms for further analysis.

### Survival analysis, purity estimation, and enrichment analysis

Overall survival comparisons were conducted and visualized via the survival and survminer R packages. Tumor purity was estimated via the estimate R package [129]. Gene Ontology enrichment analysis was performed via the clusterProfiler R package [130] with reference to the MsigDB H and C5 collection gene list [131, 132]. The gene lists of established oncogenes and cancer hallmark genes were obtained from the Gene Ontology Consortium [133] and the COSMIC database [134], respectively.

### Bulk RNA-seq, scRNA-seq, and single-cell TCR (scTCR-seq) analysis

Bulk RNA-seq FASTQ files were aligned via Salmon and normalized to TPM for further analysis. The GBM single-cell Smart-seq2 data were aligned as isoforms/transcripts via Salmon. The 10X scRNA-seq FASTQ files were first aligned by cellranger (v7), counting according to refdata-gex-GRCh38 2020-A with default parameters. Both the Smart-seq2 and 10X scRNA-seq data were loaded into the Seurat v4 R package [135] with the parameters min.cell = 3 and min.features = 200. The 10X scRNA-seq data were normalized via the LogNormalize method with default parameters. The cell type annotation was based on original papers. The downstream analysis followed the Seurat pipeline [135]. The scTCR-seq FASTQ files were aligned via cellranger (v7) vdj, referring to refdata-cellranger-vdj-GRCh38-alts-ensembl-7.0.0, with default parameters. The scTCR-seq data summary was conducted via the scRepertoire R package [136], and TCRs were matched back to the scRNA-seq data via the singleCellTK R package [137]. Single-cell paired TCR complementarity-determining region 3 (CDR3) sequences were further clustered by TCRdist [61]. The viral TCR CDR3 sequences were identified by matching them with the VDJdb [138].

### Glioma cohort and cell subtype/phenotype classification

The glioma Verhaak molecular subtype [4, 5] for the bulk RNA-seq data and the neoplastic cell phenotype [37] for the scRNA-seq data were identified via the ssgsea.GBM.classification R package [5]. We first constructed 5000 virtual random samples on the basis of the gene expression matrix by randomly selecting expression values from the same genes in real samples. The ssGSEA score for each subtype category was then calculated for both real and virtual samples, and the subtype was identified as the one that had the least random sample match. CD8^+^ T subclones were identified via the ProjecTILs R package [139] referring to CD8T_human_ref_v1.

### HLA-I genotype and antigenic epitope prediction

The HLA-I genotype was identified on the basis of a bulk RNA-seq FASTA file through OptiType [140]. Peptide-MHC (pMHC) binding affinity was predicted via NetMHCpan-4.1 [141], and the intracellularly presented epitope was predicted via NetCTLpan [142]. We collected epitopes with strong/weak MHC affinity (strong: rank < 0.5% and weak: 0.5% < rank < 2%) from isoforms/transcripts of the same gene and retained the epitopes detected only in our target isoforms. The threshold set for stratifying potential immunogenic peptides at the RNA level was TPM > 33, as previously reported [98].

### Tumor cell identification and DNA open field assessment

Single-nuclei (sn)RNA-seq data were loaded into the Seurat v4 R package [135] with the parameters min.cell = 3 and min.features = 200, normalized by the LogNormalize method, and then we used the Harmony R package [143] to remove batch effects. Neoplastic cells were identified on the basis of the predicted DNA copy number via the CopyKAT R package [144]. Normal cell types were identified by canonical markers (CD14 for myeloid cells, MOG and GJA1 for glial cells, and CD3E for T cells). To integrate the scATAC-seq data, we combined the peaks in the different samples into common peaks via the GenomicRanges R package [145]. The scATAC-seq data with common peaks as features were loaded into the Signac R package [146]. After TF-IDF normalization and singular value decomposition, batch effects were removed via the Harmony R package. The predicted gene activity value from scATAC-seq data was generated via the CreateGeneActivityMatrix function in Seurat v3 [147]. We mapped the scATAC-seq data to the snRNA-seq data to annotate the cell types in scATAC-seq cells via anchors and label transfer. The peaks were annotated via the TxDb.Hsapiens.UCSC.hg38.knownGene R package.

### Alternative splicing junction identification via 10X scRNA-seq

STAR was used to detect and quantify the number of SJs [148]. After the features and cells between the cellranger count output and the STAR SJ matrix output were matched, the data were further loaded into MARVEL to detect the target SJ in the 10X scRNA-seq data [149].

### Bulk RNA extraction and library construction

Bulk RNA was extracted with a Quick-DNA/RNA MiniPrep Kit (Zymo Research, Cat. #D7001) following the manufacturer’s protocol. The cells were pelleted via centrifugation at 1500 rpm for 5 min at room temperature. The cell pellets were resuspended in 350 µL of Tri Reagent (Zymo Research, Cat. #R2050-1-200) and vortexed to mix the samples. A total of 350 µL of molecular grade ethanol (Decon Laboratories, Cat. #CAS# 64-17-5) was added to the TRIzol mixture and placed on a Zymo Spin IIICG Column (Zymo Research, Cat. #C1006-50-G) was placed in a collection tube (Zymo Research, Cat. #C1001-50) and spun at 16,000 × *g* for 30 s. Next, 400 µL of Zymo Wash Buffer (Zymo Research, Cat. #R1003-3-48) was added to the columns and centrifuged at 16,000 × *g* for 30 s. Next, 5 µL of DNase I (6 U/µL) (Zymo Research, Cat. #E1011-A) and 75 µL of DNA Digestion Buffer were mixed and placed onto the columns (Zymo Research, Cat. #E1010-1-16) and incubated at room temperature for 15 min. Next, 400 µL of Direct-Zol RNA Prewash (Zymo Research, Cat. #R2050-2-160) was added to the column and spun at 16,000 × *g* for 30 s. This step was then, 700 µL of RNA wash buffer was added to the column and centrifuged for 1 min at 16,000 × *g*. Finally, the RNA was eluted from the columns with 50 µL of DNase/RNase-free water (Zymo Research, Cat. #W1001-30) and spun at 16,000 × *g* for 30 s. After the RNA was isolated, 5 µL of RNA was used to ensure quality control, which was run on a 4200 Agilent Tape Station (Agilent, Cat. #G2991BA) using high-sensitivity RNA screen tapes (Agilent, Cat. #5067-5579). The RNA was then subjected to mRNA library preparation (poly A enrichment) and sequenced on an Illumina NovaSeq X Plus Series (PE150).

### Upstream transcription factor exploration and validation

The transcription start site (TSSs) (or promoter regions) of RCAN1-1 and RCAN1-4 (2000 bp long) were obtained from the UCSC Genome Browser [150] and used to predict potential transcription factor binding. Potential transcription factor binding was predicted via various databases, including JASPAR [40], KonckTF [41], ENCODE [42], Cistrome [43], and hTFtarget [44]. C/EBPβ overexpression (OE) and shRNA lentiviruses were acquired from Dr. Baoli Hu’s laboratory at the University of Pittsburgh and transduced into GBM tumor cells. Changes in RCAN1-4 expression were evaluated by western blotting (WB) 72 h after lentiviral transduction.

### Chromatin immunoprecipitation (ChIP)-PCR

A ChIP‒PCR assay was performed to confirm the interaction between C/EBPβ and the RCAN1-4 promoter region. C/EBPβ-binding DNA was extracted via a Pierce Magnetic ChIP Kit (Thermo Fisher, Cat. #26157) according to the manufacturer’s protocol. A total of 2–4 × 10^6^ cells per GBM tumor cell line were trypsinized and washed twice with PBS. The cells were then resuspended in 1% (v/v) formaldehyde for cross-linking at room temperature for 10 min with shaking. After quenching with 0.125 M glycine, the cells were lysed, and the extracted nuclei (chromatin) were further fragmented with micrococcal nuclease (MNase, Thermo Fisher, Cat. #88216) and sonicated on ice (40% amplitude). The fragmented chromatin was incubated with 5 µg of C/EBPβ antibody (Santa Cruz Biotechnology, Cat. #sc-7962, Clone: H-7) or normal rabbit IgG overnight at 4 °C with rotation. C/EBPβ-cross-linked DNA was collected using Protein A/G Magnetic Beads. After DNA purification, RCAN1-1 and RCAN1-4 TSS DNA was quantified via qPCR via PerfeCTa FastMix II Low ROX (Quantabio, Cat. #95120-250) and normalized to total chromatin (input). Each reaction was set up according to the manufacturer’s recommendations. qPCR was performed on an Applied Biosystems QuantStudio 3 Real-Time PCR System (Thermo Fisher Scientific) under the following standard cycling conditions: initial denaturation at 95 °C for 2 min, 40 cycles at 95 °C for 15 s, and a final extension at 60 °C for 60 s. Melt curve analysis was conducted to confirm amplification specificity. Data analysis was performed via the ∆∆Ct method. The primers used for RCAN1-1 and RCAN1-4 were as follows:

RCAN1-1 primer 1: forward 5′-CCTCTTGGGTTTAGCTCCCTG-3′; reverse 5′- GCCATGTGCACGGTTCTATG-3′;

RCAN1-1 primer 2: forward 5′-AGGCATTGTTATCATGGTCTAGTT-3′; reverse 5′- CCCTGGCAAGTGGTTAGATG-3′;

RCAN1-1 primer 3: forward 5′-TGGTTGGCTCTAAGCAACTTC-3′; reverse 5′- GGAACCGTCATACTGTGAAAGG-3′;

RCAN1-4 primer 1: forward 5′- TGCCACAGACCTTCATCCTT-3′; reverse 5′- ACTCAGACAACACGCTCCTC-3′;

RCAN1-4 primer 2: forward 5′- GCCACAGACCTTCATCCTTT-3′; reverse 5′- CCATTCAGCTACTCAGACAACA-3′;

RCAN1-4 primer 3: forward, 5′- TCACCCGGACTTTGGTTTC-3′; reverse, 5′- GGATGAAGGTCTGTGGCAATA-3′.

Sanger sequencing (Genewiz, NJ, USA) was performed on the PCR products to verify the promoter site.

### CUT&RUN data analysis

CUT&RUN datasets (PRJEB39793 [151]) for C/EBPβ (ERX4391629, ERX4391631, ERX4391630) and IgG control (ERX4391632) from untreated MDA-MB-231 breast cancer cells were trimmed to remove adapters with CutAdapt (v2.10) and aligned to the hg38p14 reference assembly (GCF_000001405.40) via Bowtie2 (v2.4.5) with parameters --end-to-end --very-sensitive --no-mixed --no-discordant -I 10 -X 700 --dovetail and filtered to remove secondary, supplemental, and unmapped reads. The samples were normalized via *S. cerevisiae* S288C spike-in alignment to (GCF_000146045.2). Significantly enriched CEBPB peaks were identified via SEACR (v1.3) with IgG control in relaxed mode.

### Sample preparation for MS analysis

Purified immunopeptide samples were prepared for LC‒MS/MS analysis at Bioinformatics Solutions, Inc. (Ontario, Canada). Briefly, the cell pellets were lysed to extract whole proteins. HLA‒peptide complexes were enriched via the Neo DiscoveryTM immunopeptidome enrichment kit (Bioinformatics Solution Inc.). The extracted proteins were incubated overnight at 4 °C with the anti-HLA-I antibody cross-linked beads provided in the kit. After washing and elution, the HLA complexes with bound peptides were separated and desalted via C18 columns (GL Science). The separated peptides were then dried with a speed‒vacuum instrument and stored at −20 °C until analysis. A small portion of the sample from each step was subjected to QC assessment via Western blot.

### Mass spectrometry data acquisition

Samples were resuspended in 0.1% formic acid prior to MS analysis. For each run, the resuspended sample was separated by nanoflow liquid chromatography via an Ultimate 3000 chromatography system (Thermo Fisher) and then injected into a Thermo Orbitrap Fusion Lumos (Thermo Fisher). Liquid chromatography was performed using a constant flow of 0.30 µL/min and a 15 cm reversed-phased column with a 75 µm inner diameter filled with Reprosil C18. Mobile phase A was 0.1% formic acid, and mobile phase B was 99.9% acetonitrile and 0.1% formic acid. The separation was carried out over 90 min as follows: 5% (v/v) B was linearly increased to 100% (v/v) B over 83 min and held constant for 3 min to clean the column. The B percentage was then set back to 2% (v/v) in the final 3 min.

MS/MS data acquired on a Thermo Orbitrap Fusion Lumos instrument for each sample were carried out in data-dependent acquisition mode with a cycle time of 2 s. In the first round, MS1 scan data were obtained at 120,000 resolution (at 400 *m*/*z*) with a mass range of 350–900 *m*/*z*. The automatic gain control (AGC) was set to 400,000, with an auto maximum ion injection time. The radio frequency lens was set to 30%. The charge state filter was set to 2–3, and the dynamic exclusion was set to 10 s. Isolation for MS2 scans was performed in the quadrupole, with an isolation window of 0.6 Da. MS2 scan data were acquired at a resolution of 30,000 *m*/*z* in the Orbitrap, with a 100,000 AGC target and 150 ms ion injection time. The scan range of MS2 was also set to auto. Higher energy collisional dissociation (fixed normalized collision energy: 35%) was used for generating MS2 spectra, with the number of microscans set to 1.

### Mass spectrometry data analysis

All the raw data files collected via mass spectrometry were analyzed via the PEAKS Studio 12 (Bioinformatics Solutions Inc., Waterloo, Ontario, Canada) DeepNovo Peptidome workflow [152]. The precursor and fragment ion mass error tolerances were set to 10 ppm and 0.02 Da, respectively. The oxidation of methionine was set as a variable modification. All the data were searched against the Human reviewed UniProt database, with the peptide FDR set to 1%. For identification of noncanonical peptides, de novo sequencing was performed via the DeepNovo algorithm [153].

### Functional validation of antigen peptides

To functionally validate the ability of our predicted peptide antigens to prime and activate cytotoxic CD8^+^ T cells, we followed a protocol for assessing antigen-specific activation and expansion of naive human CD8^+^ T cells by Wolf and Greenberg [55]. Briefly, healthy donor-derived PBMCs were isolated from HLA-specific LRS chambers (Stanford Blood Center) to generate peptide-pulsed antigen-presenting cells (APCs) via IL-4, GM-CSF, IFN-γ, and LPS, as described previously [55]. Naive CD8^+^ T cells from the same donor were isolated via magnetic beads (MojoSort, Cat. #480046) and then cultured with peptide-pulsed APCs (T cell priming) for a total of 12 days. Antigen-experienced CD8^+^ T cells were then cultured with autologous target cells loaded with corresponding peptides (antigen recall) or primary GBM cell lines, SF10281 and SF10360, to determine functionality and peptide immunogenicity. To determine antigen-specific activation, an HLA class I-reactive CEF (CMV, EBV, and influenza virus) peptide pool, no-peptide/low-affinity peptides, and an anti-human MHC class I mAb (BioXcell, Cat. #BE0079) were used as positive and negative controls, respectively. T cell function and phenotypes were assessed by IFN-γ release, cytotoxicity, and flow cytometry.

### ELISA and LDH-based cytotoxicity assay (ELISA-based)

The medium was collected and stored at −20 °C for up to 7 days or at 4 °C for up to 2 days until use. Human IFN-γ ELISA (BioLegend, Cat. #430104) and the CyQUANT LDH Cytotoxicity Assay (Invitrogen, Cat. #C20300) were used according to the manufacturers’ protocols. The plates were analyzed on a Biotek Synergy2 microplate reader at a wavelength of 450 nm and a background wavelength of 570 nm. The percentage of cytotoxicity was calculated as [(experimental effector spontaneous − target spontaneous)/(target maximum − target spontaneous)] × 100.

### Enzyme-linked immunosorbent spot (ELISPOT) assay analysis

A human IFN-γ single-color ELISPOT assay (Cellular Technology Limited; CTL, Cat. #hIFNgp-1 M/2) was used to measure preexisting T cells specific for testing peptides in PBMCs from healthy donors and patients with GBM. Frozen tumor-infiltrating T cells (TILs) and PBMCs from adult GBM patients and pediatric GBM patients were obtained from UPMC Hillman Cancer Center and the CBTTC biobank, respectively. HLA-A24^+^ patients were identified by flow cytometry using an anti-human HLA-A24 monoclonal antibody (LS Bio, Cat. #LS-C179733-100). Owing to the limited number of frozen cells, TILs were expanded according to the rapid expansion protocol (REP) [154, 155]. TILs were expanded in CTS™ OpTmizer™ T-Cell Expansion Media (Thermo Fisher, Cat. #A1048501) and then incubated for 5 days in AIM V™ media (cytokine-free, Gibco™, Cat. #12055091) supplemented with 1% human AB serum (GeminiBio, Cat. #100-512-100) prior to the assay. A total of 2.5–5 × 10^5^ cells were incubated with antigen peptides (10 µg/mL) in CTL-Test™ media (serum-free, CTL Cat. #CTLT-005) overnight, and the number of IFN-γ-producing T cell units was measured. Dynabeads™ Human T-Activator CD3/CD28 for T cell expansion and activation (Gibco™, Cat. #11131D) served as a positive control for determining the total number of IFN-γ-secreting T cells, and DMSO served as a blank (background). IFN-γ-forming spots were visualized via a BCIP/NBT phosphatase substrate. ELISPOT assay plates were imaged via a CTL Series 6 Universal-V ImmunoSpot analyzer, and spot counts were analyzed with ImmunoSpot software (version 2.6). The results were normalized to the blank to remove nonspecific background and calculated as IFN-γ cytokine-forming units (CFUs) per 5 × 10^5^ T cells. Each sample was assayed 2–4 times by two independent investigators (one blinded to the results of this study), with technical triplicates per sample in each experiment.

### Flow cytometry

Single-cell suspensions were stained for cell surface markers with fluorescently labeled anti-human antibodies and Fc receptor blocking solution (Human TruStain FcX; BioLegend, Cat. #422302) to reduce nonspecific binding in FACS buffer (phosphate-buffered saline (PBS) with 1% BSA, Fisher Scientific, Cat. #BP1600-1) for 30 min at 4 °C per the manufacturer’s guidelines. After cell surface staining, the cells were washed with FACS buffer and fixed with BD Cytofix/Cytoperm™ Fixation and Permeabilization Solution (BD Biosciences, Cat. #554722). For intracellular staining, the cells were washed with eBioscience™ permeabilization buffer (Invitrogen™, Cat. #2831094) and stained with anti-human antibodies against intracellular markers in permeabilization buffer for 45–60 min at 4 °C. The cells were stained for CD3 (BioLegend, Cat. #300308), CD14 (Thermo Fisher, Cat. #363-0149-42), CD8 (BioLegend, Cat. #300912), and CD45RO (BioLegend, Cat. #304212) (W/O cell viability) and intracellularly stained for active caspase-3 (BD Biosciences, Cat. #560627) and IFN-γ (BioLegend, Cat. #502538). The cells were washed with FACS buffer, resuspended in PBS, and analyzed by flow cytometry. All the samples were analyzed via a BD LSRFortessa (BD Biosciences). The data were analyzed via FlowJo V10 data analysis software (FlowJo LLC).

### Tumor cell culture

The tumor cells were cultured according to the recommended guidelines. The cells were maintained in a liquid nitrogen tank prior to use and were tested for mycoplasma regularly via MycoAlert (Lonza). The cells were cultured (5% CO2, 37 °C) at a density of 70% in T-75 in DMEM supplemented with 10% heat-inactivated fetal bovine serum (GeminiBio, Cat. #100-500). The RCAN1.4 knockout (KO) KNS-42 cell line was constructed by transfecting the RCAN1 human CRISPR/Cas9 KO plasmid (Santa Cruz Biotechnology, Cat. #sc-402694) and subsequently sorting the cells with GFP after 72 h.

### Transfection and Western blot (WB)

The cells were seeded in 6-well plates overnight before transfection. The plasmids or siRNAs were incubated in Opti-MEM (Gibco™, Cat. #31985088) for 20 min and then transfected for 48–72 h following the Lipofectamine 3000 protocol (Thermo Fisher, Cat. #L3000150). We then used 1% SDS buffer (10% SDS, 0.5 M Tris-HCl, Milli-Q-H2O, and 100% glycerol, pH 6.7) supplemented with protease and phosphatase inhibitors (Thermo Fisher, Cat. #78440) to lyse the transfected cells. A BCA assay (Thermo Fisher, Cat. #23225) was used to quantify the protein concentrations. Equal amounts of protein were loaded into 4–15% Mini-PROTEAN® TGX™ Precast Protein Gels (Bio-Rad, Cat. #4561084) for separation and then transferred to methanol-preactivated PVDF membranes (Sigma‒Aldrich, Cat. #3010040001, semidry protocol). The membranes were blocked in PBS containing 0.1% Tween-20 (PBST) and 5% milk for 1 h. After incubation in 5% BSA overnight with the primary antibody (RCAN1, Santa Cruz Biotechnology, cat. #sc-377507; C/EBPβ, Santa Cruz Biotechnology, cat. #sc-7962; Vinculin, Santa Cruz Biotechnology, cat. #sc-25336; α-tubulin, active motif, cat. #39527), the membranes were washed 3 times before being incubated with the secondary antibody in PBST with 5% milk for 1 h. The membranes were visualized via an enhanced chemiluminescence protocol (Thermo Fisher, cat. # 32106) in a dark room, and the protein band intensity was quantified via ImageJ software and normalized to that of the internal reference protein. Commercial normal human whole-tissue lysates from Novus Biologicals were used to assess endogenous RCAN1-4 protein expression. The following organ lysates were analyzed: brain (Cat. #NB820-59177), liver (Cat. #NB820-59234), lung (Cat. #NB820-59239), skeletal muscle (Cat. #NB820-59253), spleen (Cat. #NB820-59259), small intestine (Cat. #NB820-59255), and heart (Cat. #NB820-59217).

### Single-cell RNA extraction and library construction

Peptide-primed human CD8^+^ T cells from healthy donors were cocultured with SF10360 tumor cells overnight as described previously [55]. The cells were harvested and resuspended in 100 µL of FACS buffer. The Fc receptor was blocked using Human TruStain FcX (BioLegend, Cat. #422302) and incubated in the dark for 10 min at 4 °C. The cells were subsequently stained with CD45RO FITC (BioLegend, Cat. #304212, Clone: UCHL1), CD8a APC (BioLegend, Cat. #300912, Clone: HIT8a), CD3 PE (BioLegend, Cat. #300308, Clone: HIT3a), CD137 APC/Fire 750 (BioLegend, Cat. #309834, Clone: 4B4-1), Ghost Dye Violet 510 Viability Dye (Tonbo Biosciences, Cat. #13-0870-T100), and CD107a BV421 (BioLegend, Cat. #328626, Clone: H4A3) for 30 min in the dark at 4 °C. The cells were then washed and sorted on a BD FACSAria™ III Cell Sorter, and the cells positive for CD3, CD8, CD45RO, CD107a, and CD137 and negative for live/dead staining were sorted.

After sorting, the cells were resuspended at a concentration of 1600 cells/µL in buffer, loaded onto a Chromium Next Gem Chip K (10x Genomics, Cat. #PN-1000287), and placed within a Chromium Controller (10x Genomics, Cat. #PN-120223;120246). The product was then removed from the chromium controller and subjected to cDNA amplification following the manufacturer’s protocol. The quality of the amplified cDNA was tested by running the cDNA on a 4200 Agilent Tape Station (Agilent, G2991BA) via high-sensitivity DNA screen tapes (Agilent, 5067-5592). The amplified cDNA was then subjected to library construction, where the cDNA was fragmented, and then end-repair and A-tailing, adapter ligation, and sample index PCR were performed. cDNA libraries were sequenced on a Nova Seq x Plus Series PE150 (Illumina) using the following run cycle: 151 + 10 + 10 + 151. Raw scRNA-seq GEX reads were barcoded-deduplicated and aligned to the human reference GRCh38 2020-A via cellranger count v7.1.0. Raw scRNA-Seq TCR reads were barcoded-deduplicated and aligned to the human reference GRCh38 v7.0.0 via cellranger V(D)J v7.10. In the downstream analyses, potential tumor cells were identified and excluded as previously described [156].

### Lentiviral transduction and T cell expansion

Luciferase-positive tumor cells were generated by incubating tumor cells with CMV-luciferase (firefly)-2A-RFP (Puro)/concentrated lentivirus (AMSBIO, Cat. #LVP324, 1 × 10^7^ IFU/mL) with 8 µg/mL polybrene (Sigma-Aldrich, Cat. #TR-1003) for 24 h following the manufacturer’s protocol. Luciferase-positive RFP+ tumor cells were collected via flow sorting. The target TCR sequences were identified via scTCR-seq and inserted into lentiviral vectors. The vectors were designed as murine–human hybrid TCRs [63] with the MSCV promoter [157] to improve TCR pairing and transduction efficiency. The lentivirus was synthesized by VectorBuilder (pLV[Exp]-EGFP-MSCV-TRBV(human)-TRBC1(murine)-P2A-TRAV(human)-TRAC(murine)) with a titer >10^8^ TU/mL. CD8^+^ T cells were isolated from HLA-A24^+^ healthy donor-derived PBMCs (Stanford Blood Center) via a CD8^+^ T cell isolation kit (Miltenyi Biotec, Cat. #130-096-495). Isolated CD8^+^ T cells were activated with Dynabeads™ Human T-Activator CD3/CD28 for T cell expansion and activation following the manufacturer’s protocol 48–72 h prior to lentiviral transduction. The lentivirus was added to the media at an MOI of 50:1 with 8 µg/mL polybrene and 100 IU/mL IL-2 (BioLegend, Cat. #589106) and centrifuged at 1000 × *g* for 90 min at 32 °C. Lentiviral transduction was performed twice on consecutive days. Transduced CD8 TCR-T cells were detected by flow cytometry with an anti-murine TCR constant region antibody (BioLegend, Cat. #159708, Clone: QA18A18) 3 days after transduction. Transduced T cells were expanded following the REP with the appropriate IL-2 concentration as previously described [154, 155].

### Characterization of TCR-T cell-mediated anti-peptide activity and antitumor reactivity

TCR-T cells under REPs were washed with PBS and cultured in AIM V media supplemented with 10 ng/mL IL-7 (Thermo Fisher, Cat. #200–07–100UG) or IL-15 (Thermo Fisher, Cat. #200–15–100UG) but without IL-2 for 3–4 days before being cocultured with autologous peptide-loaded target cells or tumor cells. A total of 1 × 10^5^ autologous target cells or tumor cells were plated per well in a 96-well plate in a total volume of 100 µL with peptides. The plate was washed twice with PBS and cocultured for 30 min with blocking anti-MHC-I antibody (50 μg/well; BioXCell, Cat. #BE0079, Clone W6/32) before the addition of 2 × 10^5^ TCR-T cells (E:T ratio = 2:1) to reach a final volume of 200 μL. The media were collected after 16 h for ELISA and LDH-based cytotoxicity assays. To estimate the reactivity of TCR-T cells to the target peptide, TCR-T cells were cocultured with peptide-loaded target cells and 1X protein transport inhibitor (BD Bioscience, Cat. #555029) for 16 h. Dynabeads™ Human T-Activator CD3/CD28 was used as a positive control. Then, the TCR-T cells were intracellularly stained with PE-conjugated anti-human granzyme B (GZMB; Biolegend, Cat. #396406; Clone: QA18A28) and analyzed via flow cytometry.

### Mouse orthotopic xenograft

All experimental procedures were approved by the Institutional Animal Care and Use Committee (IACUC protocol ID# 22101912) of the University of Pittsburgh. Female NOD. Cg-Prkdc^scid^ Il2rg^tm1WjI^/SzJ (NSG) mice (4–6 weeks), obtained from the Jackson Laboratory, were used for the xenograft experiments. The mice were injected with 2–4 × 10^5^ tumor cells (KNS-42: 2 × 10^5^ cells/µL, 2 µL; SF10360: 1 × 10^5^ cells/µL, 2 µL), engineered to express the firefly luciferase (ffLuc) reporter gene, into the cortex at a coordinate from the skull position of bregma of +2.50 mm medial/lateral and −3.00 mm dorsal/ventral using a micropump injector (World Precision Instruments). Intracranial tumor inoculation was performed under anesthesia (isoflurane 2.5 ml/L and oxygen 5 L/min mixture) via a stereotaxic frame for fixation (Kopf Instruments). Twenty-one days after tumor injection, the mice were randomized into 3–4 groups (*n* = 10/group) and then treated with a single intracranial tumor injection of PBS, mock-transduced T cells, or transduced TCR-T cells as previously described [106, 107]. T cells (2 × 10^6^) were washed twice with PBS and resuspended in 5 µL of PBS for each injection. Tumor growth was monitored via BLI via an IVIS Spectrum In Vivo Imaging System (PerkinElmer Inc.) and quantified via Living Image v.4.0 software (Revvity).

### Mouse spleen tissue processing

Mouse spleens were excised and passed through a 40 µm cell strainer to generate a single-cell suspension in PBS. Erythrocytes were lysed using ACK lysis buffer (Gibco, Cat. #A1049201) for 5 min. The cells were collected by centrifugation and resuspended in FACS buffer for flow cytometry.

### Assessment of in vivo toxicity

Systemic toxicity was evaluated by histopathological examination of major organs. At the study endpoint, the mice were euthanized, and the liver, kidney, heart, spleen, pancreas, intestine, bone, and brain were harvested. Organs were fixed in 10% neutral buffered formalin, paraffin-embedded, sectioned, and stained with hematoxylin and eosin. Histological evaluation was conducted in a blinded manner by a board-certified veterinary pathologist at University of Pittsburgh DLAR vet pathology services.

### Statistics and visualization

All the statistical analyses were performed via R software, version 4.1.0 (http://www.rproject.org/). Variance normality was assessed via the Shapiro test, and continuous variables that passed the normality test between groups were compared via Student’s *t*-test or one-way analysis of variance with post hoc pairwise Bonferroni correction, or were correlated via Pearson correlation. The Wilcoxon rank-sum test was used for nonparametric hypothesis testing. The frequency data between groups were compared via the chi-square test. Peptide-induced T cell cytotoxicity between peptides from the same donors/patients was estimated via paired *t*-tests. The plots were created via the ggplot2, ggpubr, ggridges, ggrepel, RColorBrewer, and pheatmap packages.

## Supplementary information

Supplementary Figures + legends

unprocessed original images of Western blots

Supplementary Tables

## Data Availability

The data can be made available upon reasonable request from the corresponding authors. Publicly available datasets are listed in the methods.
