# Supplementary material for: C/EBPβ-induced alternative splicing of RCAN1 generates a potent TCR-T target in mesenchymal glioblastoma
Source: Cell Mol Immunol. 2025 Dec 23;23(1):94–113. doi: 10.1038/s41423-025-01360-0 (PMC12753782; doi:10.1038/s41423-025-01360-0)
Supplement: Supplementary file 1 — Supplementary Figures + legends [file 41423_2025_1360_MOESM1_ESM.docx]

**Supplementary Figures**

**Supplementary Figure 1**

**
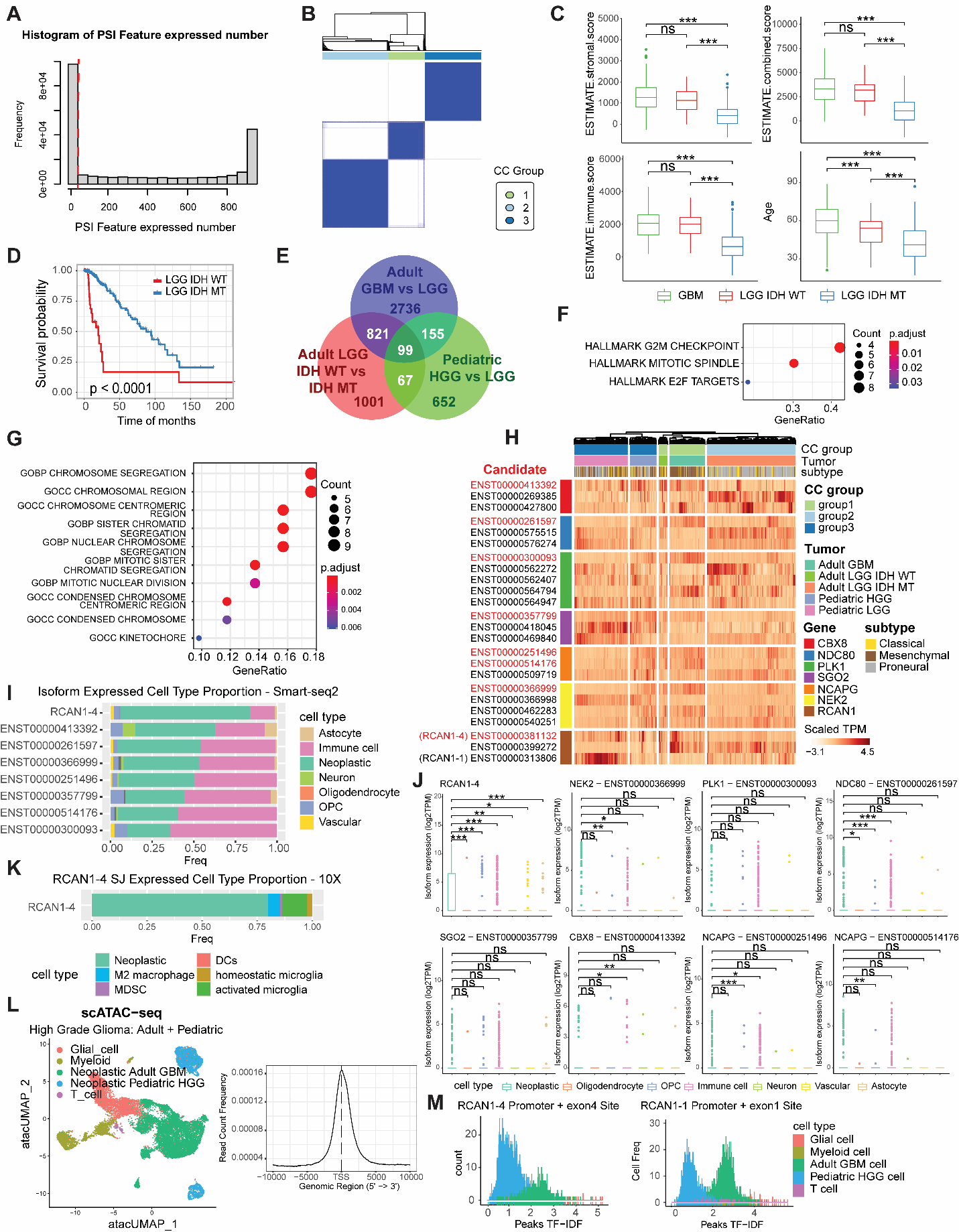
**

**Supplementary Figure 1. Identification of malignancy-related alternative splicing isoform in GBM.**

A, Histogram of the PSI index of alternative splicing events. Splicing events with PSI=0 were removed from subsequent consensus clustering. B, Consensus clustering of glioma samples based on the PSI index of splicing events. C, Immune infiltration score and age comparison of the adult glioma groups. D, Survival difference between adult IDH wildtype (WT) and IDH mutant (MT) LGG. E, Common malignancy-related isoforms from three comparisons. F-G, Gene ontology (GO) enrichment analysis of common malignancy-related isoforms on Cancer-related pathways based on H and C5 categories in MsigDB. H, Heatmap of the expression of tumor-associated isoforms and their counterparts from the same gene; the samples (column) were clustered by hierarchical clustering. I-K, Malignancy-related isoform expression and expressed proportion in GBM microenvironment at single-cell level. L, Left, UMAP of GBM sc-ATACseq data annotation; right, quality control of sc-ATACseq data. M, Chromatin accessibility of the RCAN1 isoform promoters and their first exon (exon4 of the RCAN1 gene for RCAN1-4; exon1 of the RCAN1 gene for RCAN1-1) at the single-cell level. ns, no significance; *, p <0.05; **, p <0.01; ***, p <0.001.

**Supplementary Figure 2**

**
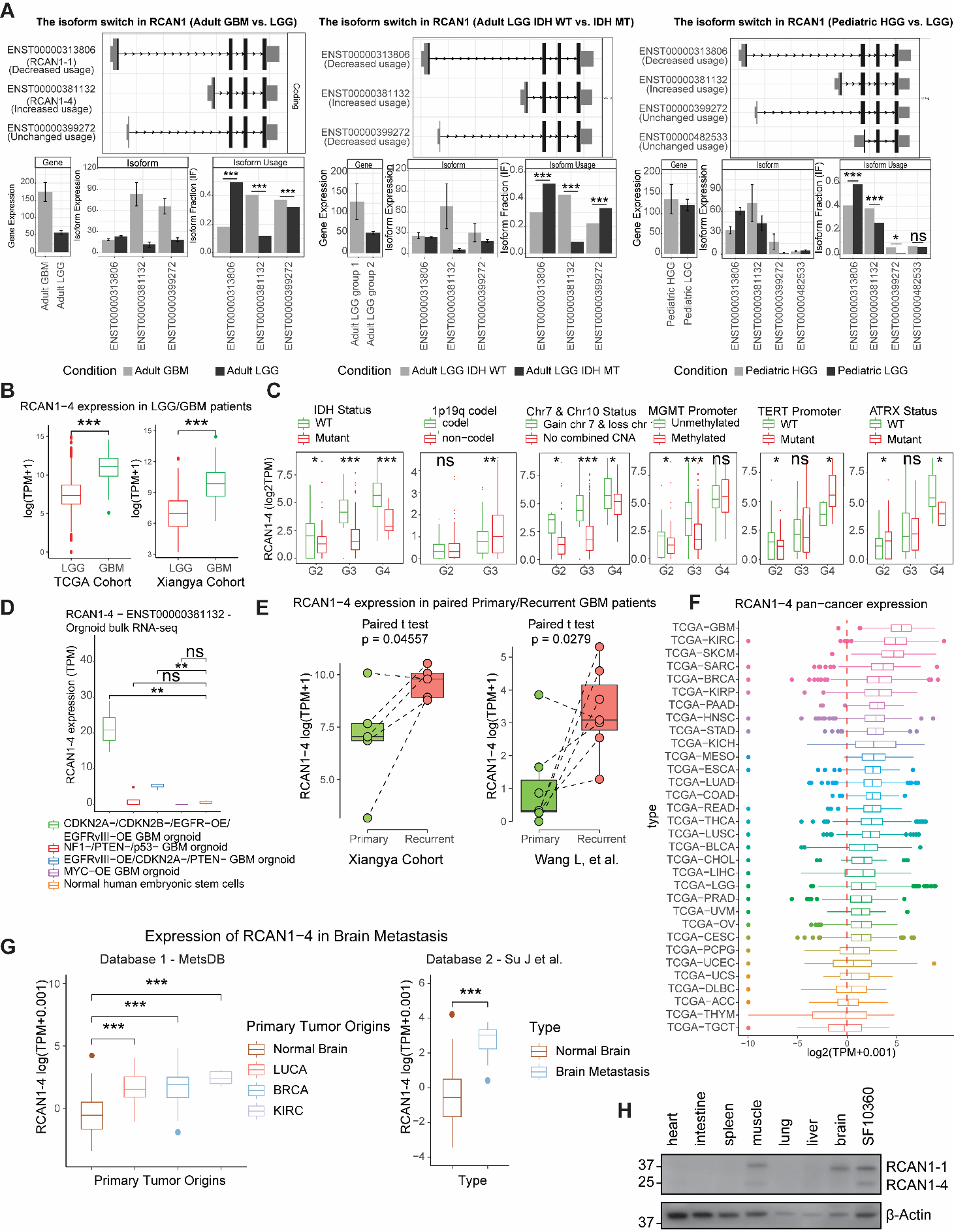
**

**Supplementary Figure 2. RCAN1-4 expression is associated with multiple malignant molecular characteristics in GBM.**

A, RCAN1 isoform switching in adult and pediatric gliomas. The comparisons were performed at gene expression, isoform expression, and isoform fraction (IF) levels. IF was defined as the percentage of isoform expression of the gene. Differential IF (dIF) higher than 0.1 was defined as significant usage alteration. B, Comparative analysis of RCAN1-4 expression between LGG and GBM patients in TCGA (left) and Xiangya (right) cohorts. C, RCAN1-4 RNA expression in adult gliomas with different molecular features. D, RCAN1-4 RNA expression in bulk RNA-seq data of glioma organoids induced by different mutations. E, Temporal analysis of RCAN1-4 expression in paired primary/recurrent GBM patients. Data is shown for two independent cohorts: the Xiangya cohort (left, n=5 pairs) and a cohort from Wang et al. (right, n=7 pairs). F, Pan-cancer analysis of RCAN1-4 expression across TCGA malignancies. G, RCAN1-4 expression in brain metastasis. Brain metastasis cohorts were sourced from two independent databases: Database 1 included brain metastasis samples from lung cancer (LUCA, n=16), breast cancer (BRCA, n=23), and kidney renal clear cell carcinoma (KIRC, n=7); Database 2 comprised a pooled cohort of brain metastases (n=187) without primary site annotation. Normal brain tissue RNA-seq data was obtained from the GTEx database (n=105). H, Protein expression of RCAN1-4 in human normal whole tissue lysates. ns, no significance; *, p <0.05; **, p <0.01; ***, p <0.001.

**Supplementary Figure 3**

**
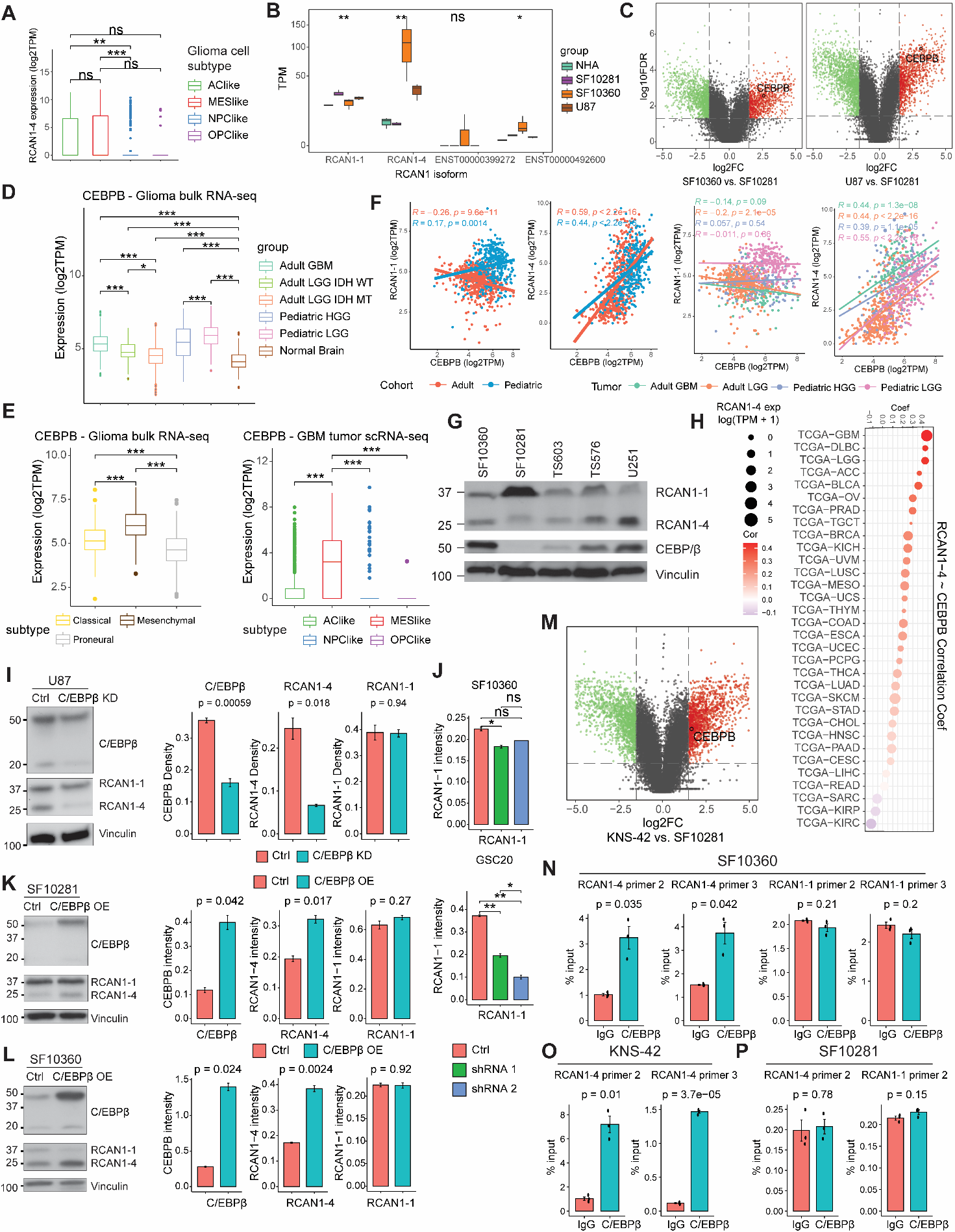
**

**Supplementary Figure 3. C/EBPβ regulates RCAN1-4 expression in GBM cells.**

A, RCAN1-4 RNA expression in glioma cells grouped by transcriptomic characteristics. B, RNA expression of RCAN1 isoforms in GBM cell lines. NHA, normal human astrocyte. C, Volcano plots of differential genes between RCAN1-4^pos^ (mesenchymal) and RCAN1-4^neg^ (classical) GBM cell lines. D, CEBPB RNA expression in glioma bulk RNA-seq data. E, Comparison of CEBPB RNA expression between gliomas with different transcriptomic characteristics at bulk tissue (left) and single-cell levels (right). F. Expression correlation of CEBPB and RCAN1 isoforms, RCAN1-4 and RCAN1-1, in glioma. G, Endogenous protein expression of RCAN1-4 and C/EBPβ across a panel of GBM cell lines. H, Pan-cancer correlation of C/EBPβ and RCAN1-4 expression in TCGA tumors. Correlation of C/EBPβ-RCAN1-4 expression across TCGA malignancies. Circles represent tumor types colored by Pearson's r (red: positive; blue: negative), sized by median RCAN1-4 expression (log(TPM+1)). I, RCAN1-4 protein alteration in U87 C/EBPβ knockdown (KD) cell lines. J, RCAN1-1 protein expression changes by knocking out C/EBPβ in mesenchymal GBM (top) and GSC lines (bottom). K, RCAN1-4 protein expression changes upon C/EBPβ overexpression in RCAN1-4^neg^ (classical) GBM cell line. L, RCAN1-4 protein expression changes by overexpressing C/EBPβ in RCAN1-4^pos^ (mesenchymal) GBM cell line. M, Volcano plots of differentially expressed genes between KNS-42 (RCAN1-4^pos^) and SF10281 (RCAN1-4^neg^) GBM cell lines. N-P, ChIP-PCR results of C/EBPβ binding to RCAN1 isoform promoter sites amplifyied by multiple PCR primers in different GBM cell lines. ns, no significance; *, p <0.05; **, p <0.01; ***, p <0.001.

**Supplementary Figure 4**

**
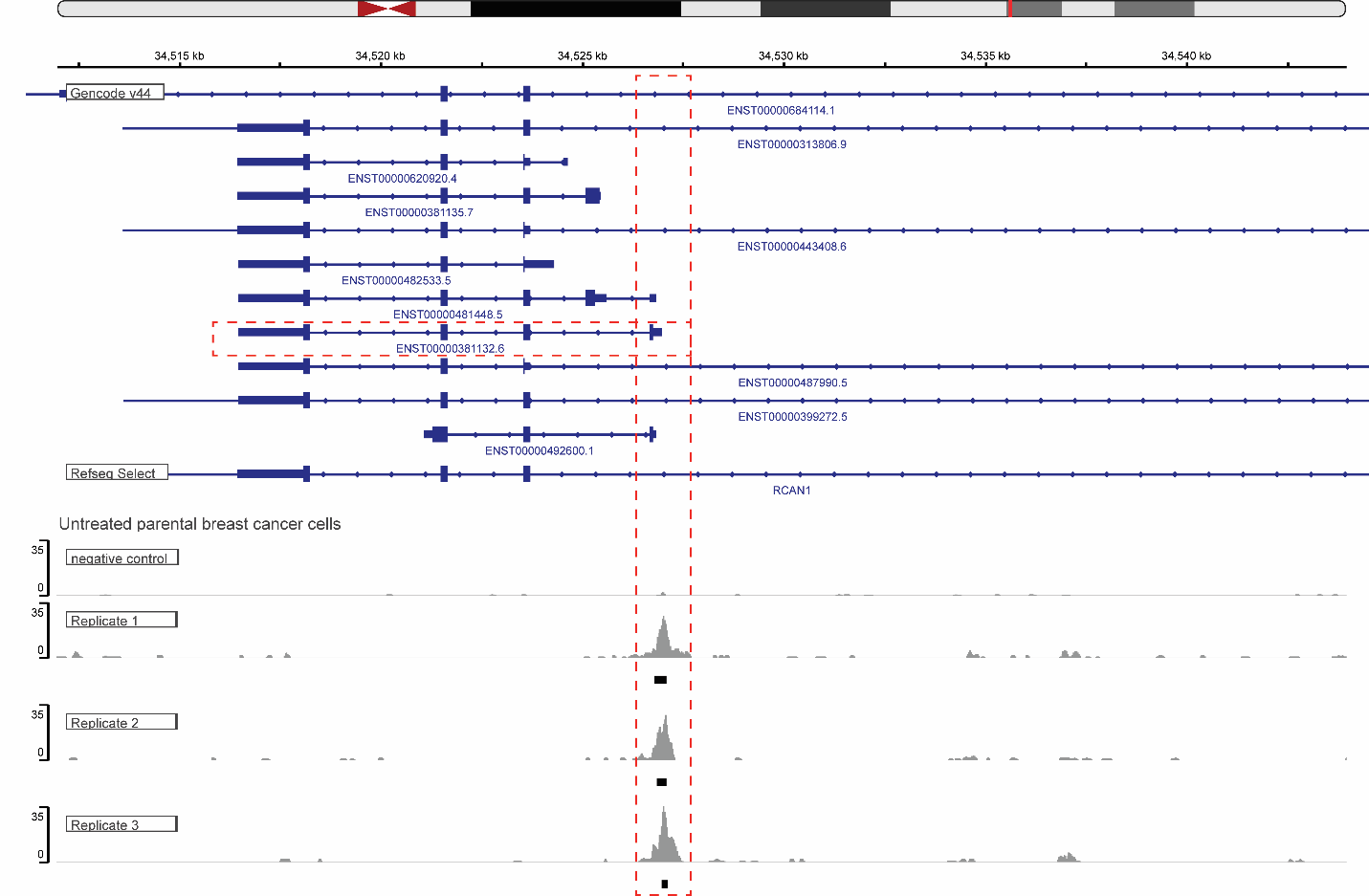
**

**Supplementary Figure 4. C/EBPβ CUT&RUN in MDA-MB-231 breast cancer cells.**

Significant enrichment at RCAN1-4 promoter region in parental, untreated cells (three biological replicates) vs. matched negative control. RCAN1-4 transcript and promoter peak highlighted (red dashed box).

**Supplementary Figure 5**

**
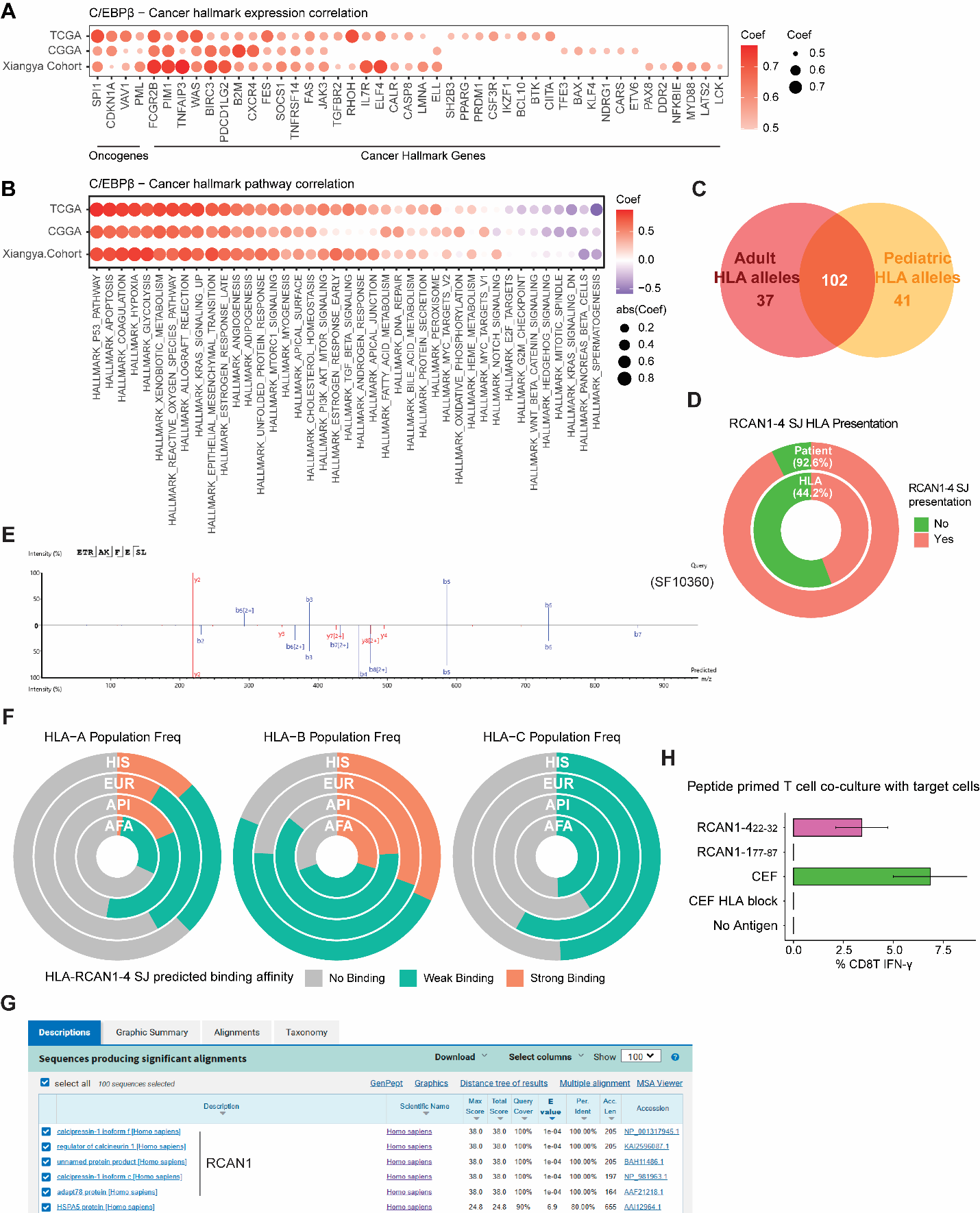
**

**Supplementary Figure 5. Identification of RCAN1-4 epitope encompassing the specific splicing junction**.

A, Correlation between C/EBPβ expression and cancer hallmark genes in GBM cohorts. B, Correlation between C/EBPβ expression and cancer hallmark gene pathways in GBM. C, HLA-I allele overlap between adult and pediatric glioma cohorts. D, Predicted proportion of RCAN1-4 SJ presentation by HLA-I alleles and glioma patients. E, Immunopeptidomic analysis of membrane-enriched MHC-I complexes from SF10360 GBM cells identifies ETRAKFSL, a peptide spanning the RCAN1-4-specific splicing junction. F, Demographic distribution of RCAN1-4 splicing junction-presenting HLA-I alleles. The population includes European descent (EUR), Asian/Pacific Islander (API), African American (AFA), and Hispanic/Latino (HIS). HLA binding affinity for the RCAN1-4 SJ epitope was predicted using NetMHCpan 4.1, with affinity level (no binding/weak binding/strong binding) defined using the tool's recommended threshold. G, Homolog detection of RCAN1-4_22-32_ using BLAST. H, Comparison of T-cell activation levels by flow cytometry when primed with different peptides. The activation level was estimated by intracellular IFN-γ expression in peptide-primed T cells co-cultured with peptide-loaded autologous target cells.

**Supplementary Figure 6**

**
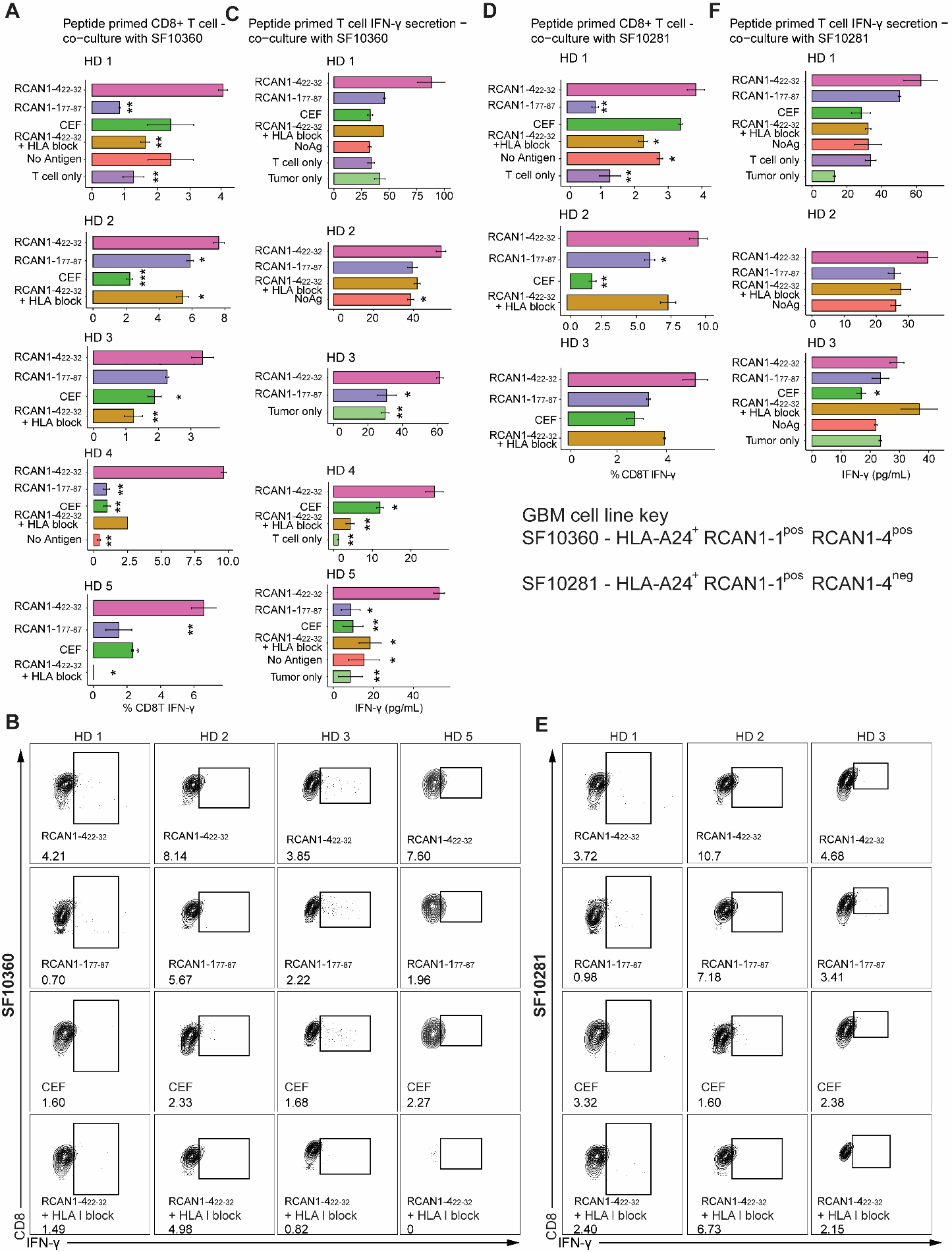
**

**Supplementary Figure 6. RCAN1-4 splicing epitope pre-primed CD8^+^ T cell reacts to RCAN1-4^pos^ GBM cells**.

A-B, Intracellular IFN-γ expression in peptide-primed CD8^+^ T cells co-cultured with SF10360. C, IFN-γ secretion by peptide-primed CD8^+^ T cells after co-culture with SF10360. D-E, Intracellular IFN-γ expression in peptide-primed CD8^+^ T cells after co-culture with SF10281. F, IFN-γ secretion by peptide-primed CD8^+^ T cells after co-culture with SF10281. These data included detailed results from all healthy donors (HDs). *, p <0.05; **, p <0.01.

**Supplementary Figure 7**

**
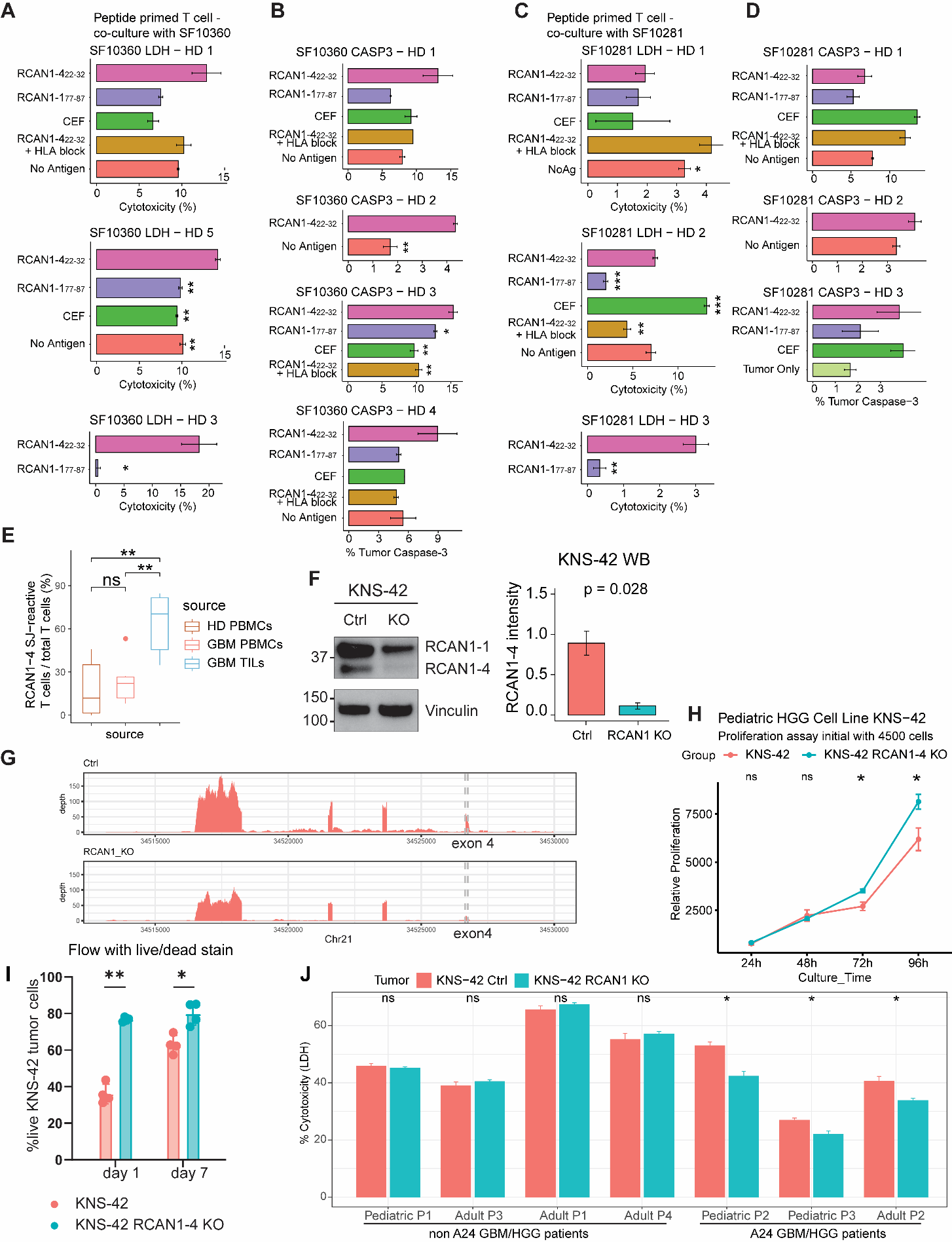
**

**Supplementary Figure 7. RCAN1-4 splicing epitope pre-primed CD8^+^ T cell tumor-killing effects on RCAN1-4^pos^ GBM cells.**

A-B, Cytotoxicity assay results of peptide-primed CD8^+^ T cell co-culture with SF10360. C-D, Cytotoxicity assay results of peptide-primed CD8^+^ T cell co-culture with SF10281. Cytotoxicity was estimated by LDH assay (A, C) and tumor cell intracellular caspase-3 expression (B, D). These data included detailed results from all healthy donors (HDs). E, Proportion of RCAN1-4 SJ-reactive T cells in total T cells (%) in PBMCs from HLA-A24⁺ healthy donors (HD, n=10), HLA-A24⁺ GBM patient PBMCs (n=6), and GBM patient tumor-infiltrating lymphocytes (TILs, n=6). Values represent (RCAN1-4-specific CFU / CD3/CD28-stimulated CFU) × 100. F, RCAN1-4 protein expression in KNS-42 RCAN1-4 KO cells. G, RNA coverage depth of the transcribed regions of RCAN1-4 isoforms in KNS-42 and KNS-42 RCAN1-4 KO cell lines. The exon4 region is indicated by the dashed line. H, Proliferation effect of RCAN1-4 knockout in KNS-42 GBM cells evaluated by Presto Blue. I, Longitudinal flow cytometric analysis of cell viability in cultured KNS-42 wild-type and RCAN1-4 knockout cells using a live/dead stain. J, Cytotoxic effect of GBM/HGG patient-expanded T cells on KNS-42 and KNS-42 RCAN1-4 KO cell lines. Patients were divided into two groups based on HLA-A24 expression levels. ns, no significance; *, p <0.05; **, p <0.01; ***, p <0.001.

**Supplementary Figure 8**

**
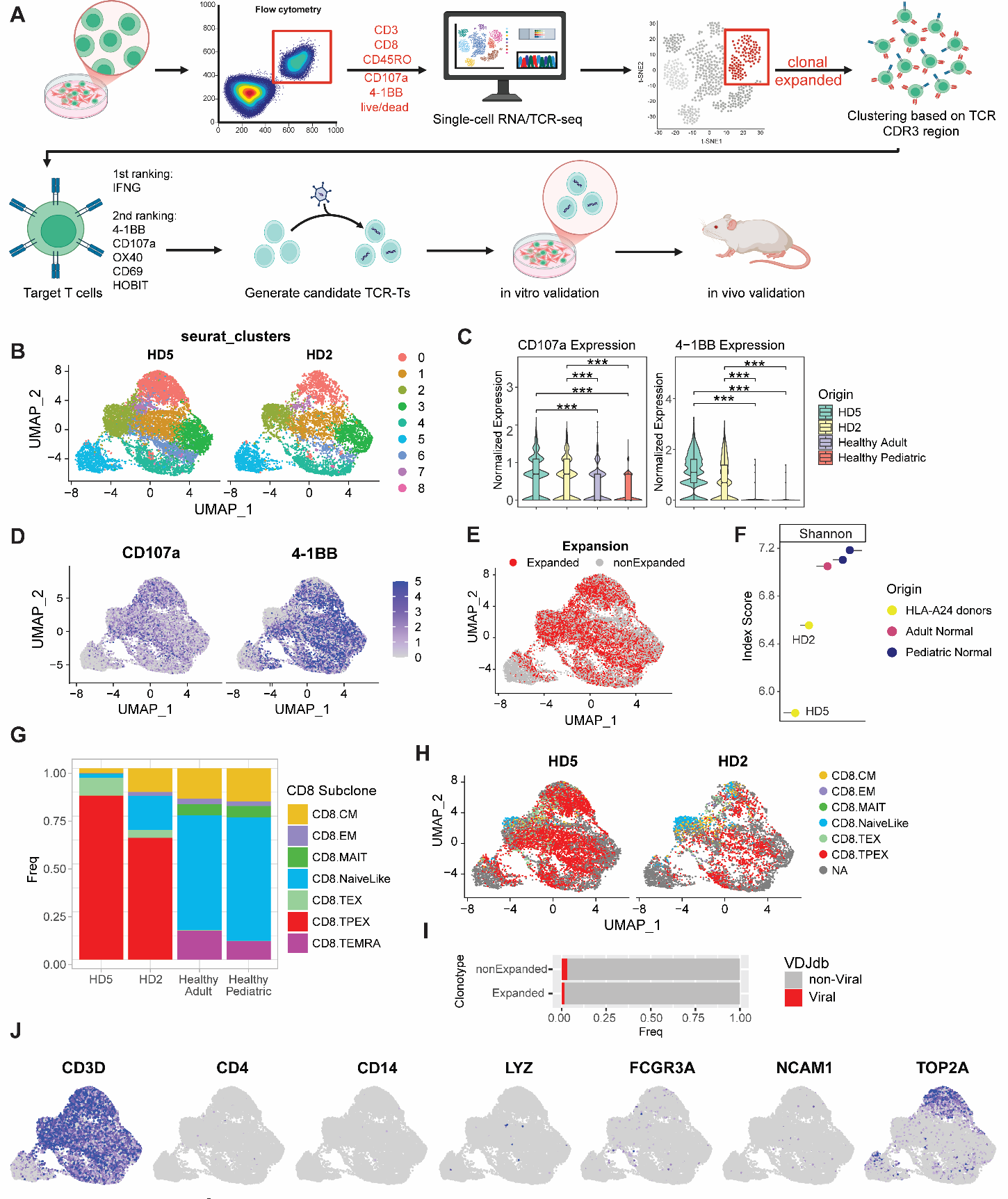
**

**Supplementary Figure 8. RCAN1-4 splicing epitope induces CD8^+^ T cell activation and clonal expansion.**

A, TCR selection and validation pipeline. B, UMAP of single cells split by healthy donors (HDs). C, Expression comparison of T-cell activation markers between RCAN1-4_22-32_-primed CD8^+^ T cells and normal CD8^+^ T cells from healthy donors. D, UMAP of T cell activation marker expression. E, UMAP of clonal expanded T cells. F, Shannon index of CD8^+^ T cells from RCAN1-4_22-32_ primed samples and normal healthy PBMCs. G, T cell subclone proportions of the different samples. H, UMAP of T-cell subclones from different samples. I, Viral TCR clonotype matching of expanded/non-expanded T cells from RCAN1-4_22-32_-primed T cell experiments. The virus-reacted TCR CDR3 region was downloaded from VDJdb database. J, UMAP of canonical marker expression. ***, p <0.001.

**Supplementary Figure 9**

**
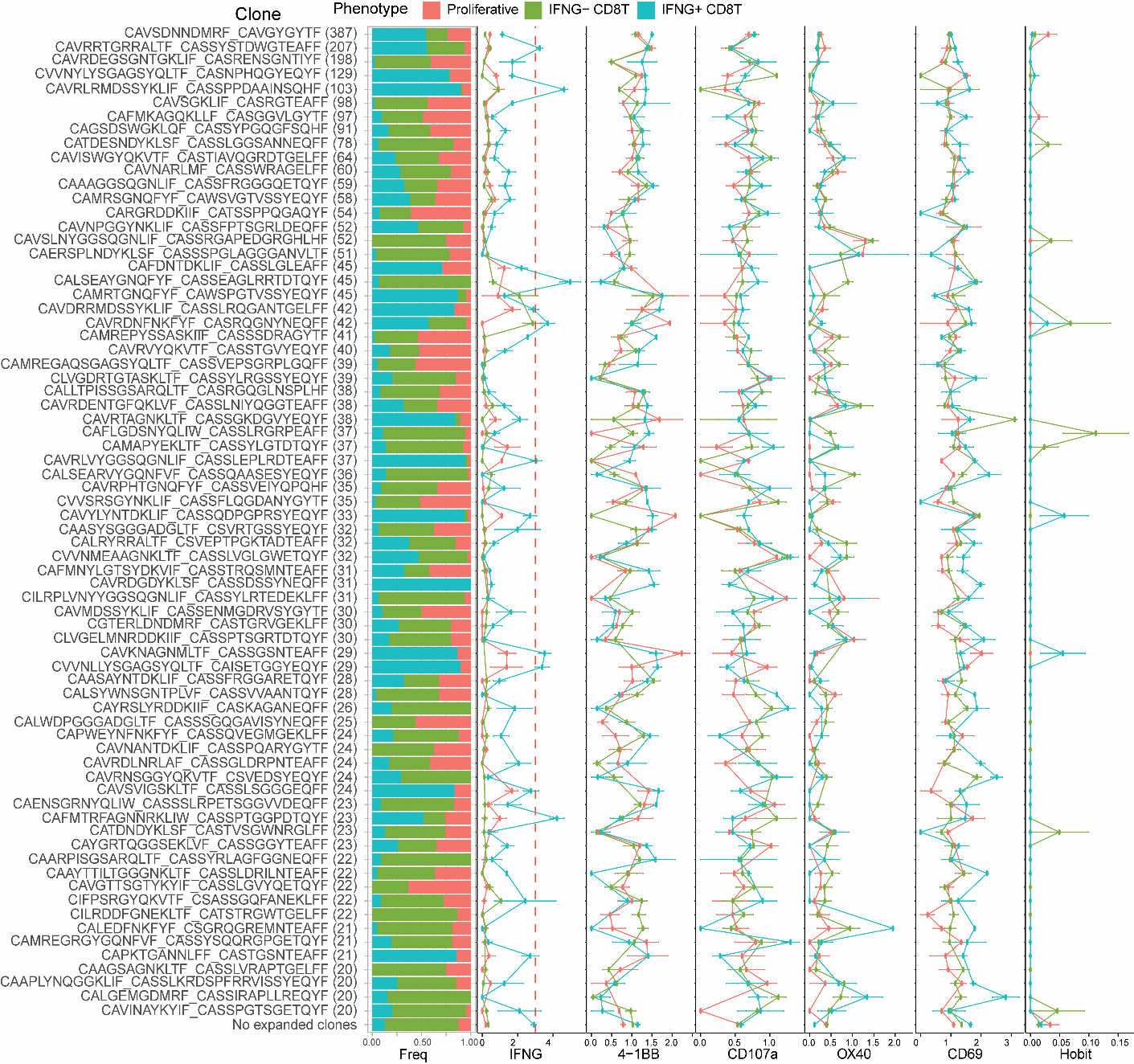
**

**Supplementary Figure 9. Clonal expanded T cell clones.**

TCR CDR3 region of T cells with clonal expansion. The expanded T cell number was next to each CDR3 sequence. The subtypes of each clonotype is shown in Figure 4B. The average expression of multiple T-cell activation genes in the three clusters is shown next to each clonotype.

**Supplementary Figure 10**

**
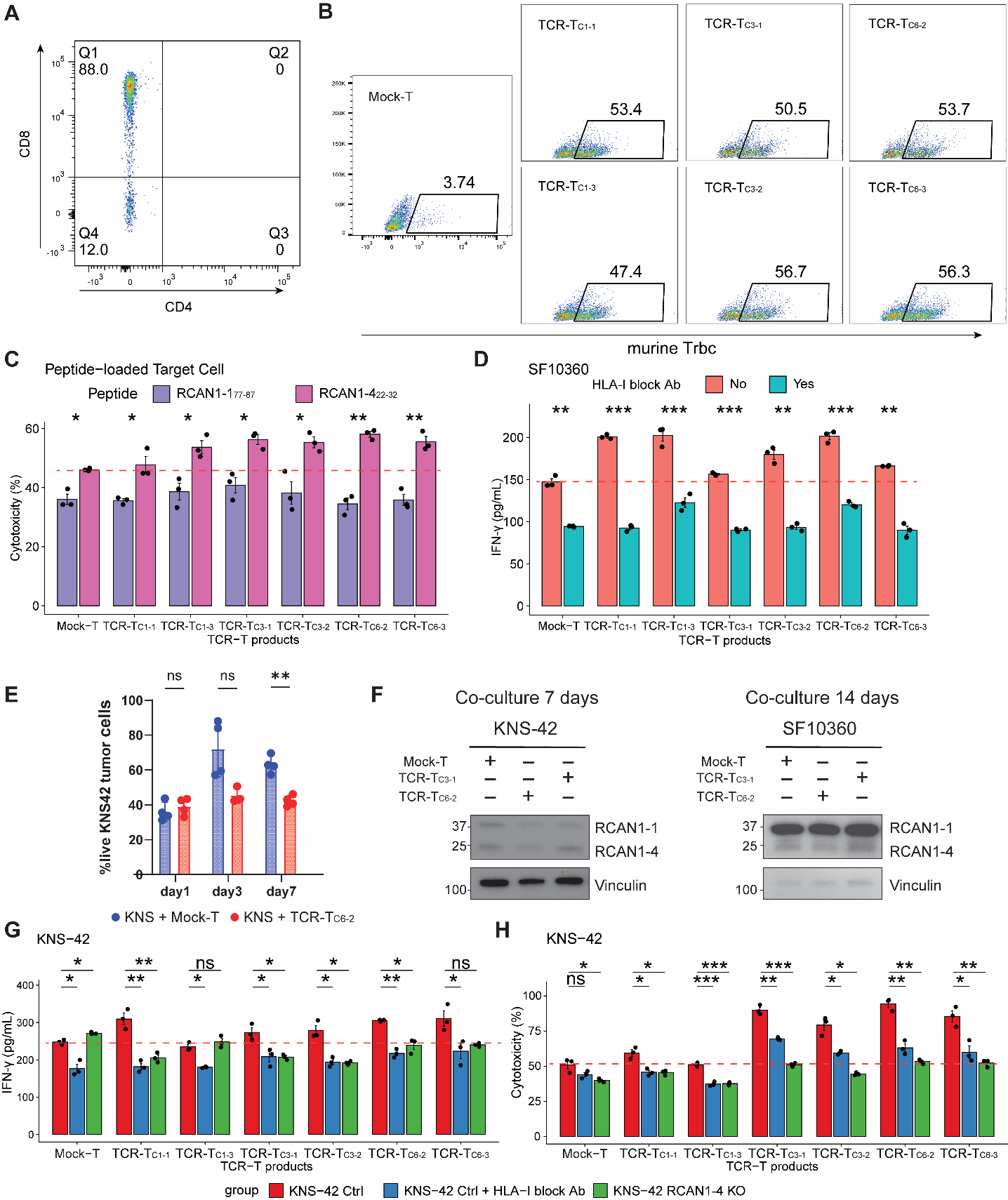
**

**Supplementary Figure 10. RCAN1-4-targeted TCR-Ts perform increased tumor-killing on RCAN1-4^pos^ GBM cells.**

A, Validation of CD8^+^ T-cell isolation using magnetic beads. B, Transduction efficiency of TCR-T cells. C, TCR-T cytotoxicity when co-cultured with RCAN1 peptide-loaded autologous target cells. D, TCR-T IFN-γ secretion levels when co-cultured with SF10360 cells. E, In vitro killing of KNS-42 GBM cells by RCAN1-4_22-32_-reactive TCR-T cells, assessed by flow cytometry with live/dead stain over time. F, Left, RCAN1-4 protein expression in KNS-42 GBM cells following 7-day co-culture with mock-T or TCR-T cells. Right, RCAN1-4 protein expression in SF10360 GBM cells following 14-day co-culture with mock-T or TCR-T cells. G-H, TCR-T activation and cytotoxicity level when co-culture with KNS-42 and RCAN1-4^KO^ KNS-42 cell line. All TCR-T used in this study were constructed based on CD8^+^ T cells from HD5 PBMCs. ns, no significance; *, p <0.05; **, p <0.01; ***, p <0.001.

**Supplementary Figure 11**

**
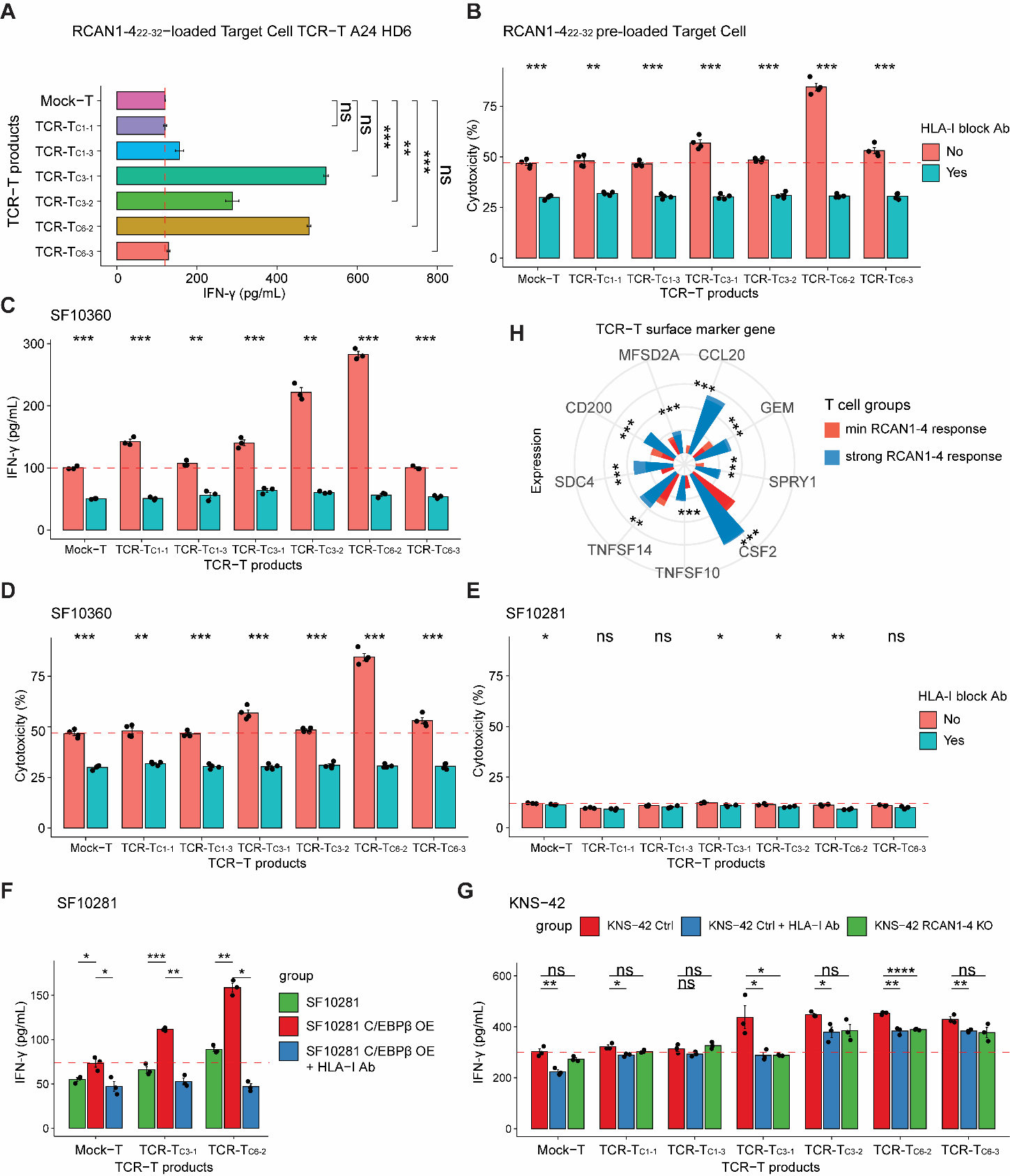
**

**Supplementary Figure 11. TCR-Ts' tumor-killing effects validation by CD8^+^ T cells from another HLA-A24^+^ healthy donor.**

This Figure validates TCR-T cytotoxicity using CD8^+^ T cells from PBMCs from another HD, HD6. A, TCR-T IFN-γ secretion levels when co-cultured with RCAN1-4_22-32_-loaded autologous target cells. B, TCR-T cytotoxicity when co-cultured with RCAN1-4_22-32_-loaded autologous target cells. C, TCR-T IFN-γ secretion levels when co-cultured with SF10360. D-E, TCR-T cytotoxicity level when co-cultured with patient-derived primary GBM cell lines. F, TCR-T IFN-γ secretion levels when co-cultured with SF10281 and SF10281 C/EBPβ OE cell lines. G, TCR-T IFN-γ secretion levels when co-cultured with KNS-42 and KNS-42 RCAN1-4 KO cell line. H, Transcriptional profiling of surface molecules in RCAN1-4_22-32_-specific CD8^+^ T cells. Strong responsive T cells (TCR-T_C3-1_/TCR-T_C6-2_ clones) vs. min responsive T cells (TCR-T_C1-1_/TCR-T_C1-3_ clones) based on in vitro reactivity to antigen-pulsed targets/GBM cells. ns, no significance; *, p <0.05; **, p <0.01; ***, p <0.001.

**Supplementary Figure 12**


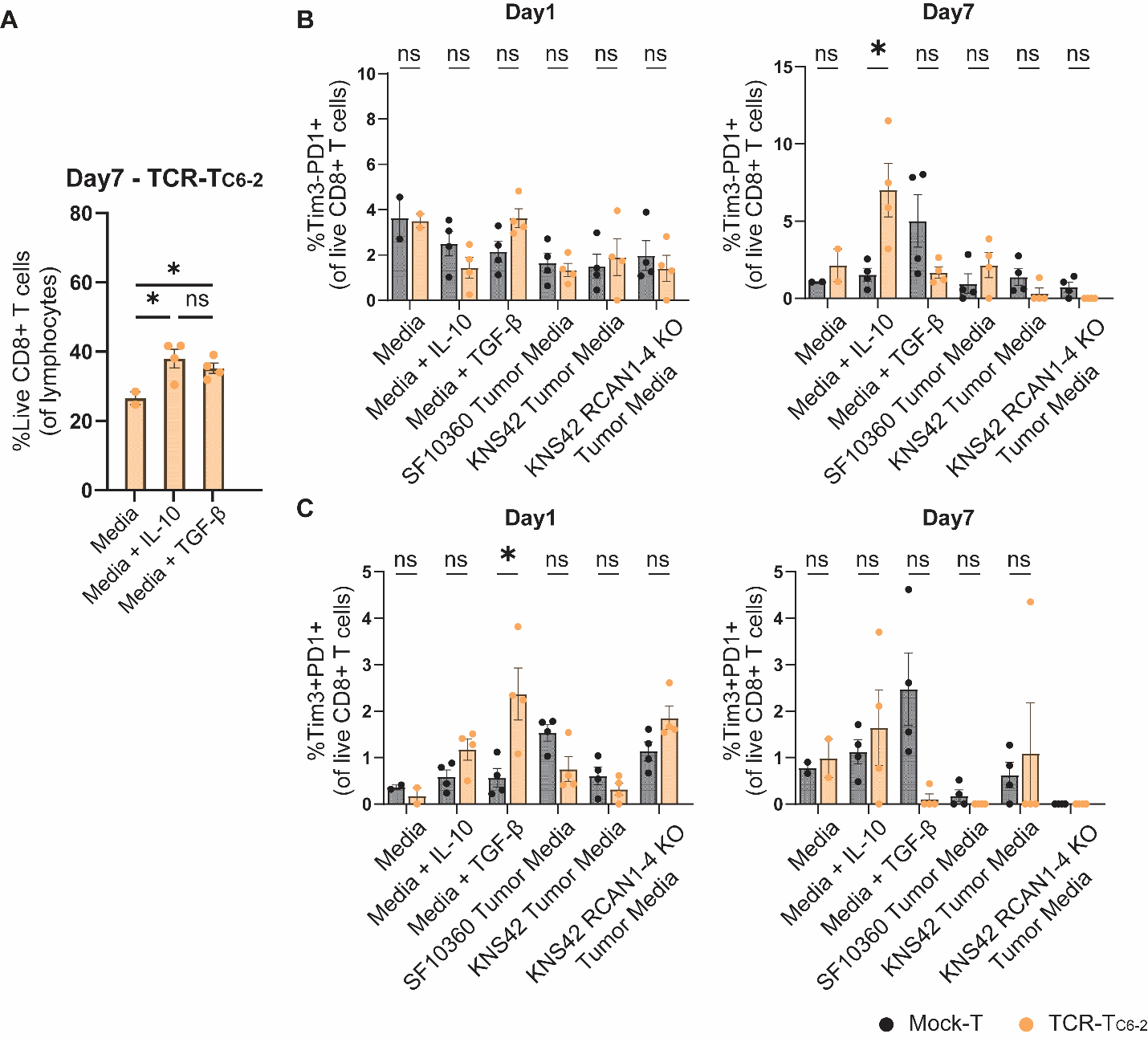


**Supplementary Figure 12. Functional characterization and persistence of RCAN1-4_22-32_-reactive TCR-T cells in an immunosuppressive microenvironment in vitro.**

A, Viability of TCR-T_C6-2_ cells following a 7-day culture in media supplemented with immunosuppressive cytokines. B-C, Expression of exhaustion markers on TCR-T cells. Flow cytometry analysis of the proportion of PD-1⁺ cells (B) and PD-1⁺TIM-3⁺ double-positive cells (C) after 1 day (left) or 7 days (right) of culture in either immunosuppressive cytokine media or conditioned tumor media.

**Supplementary Figure 13**


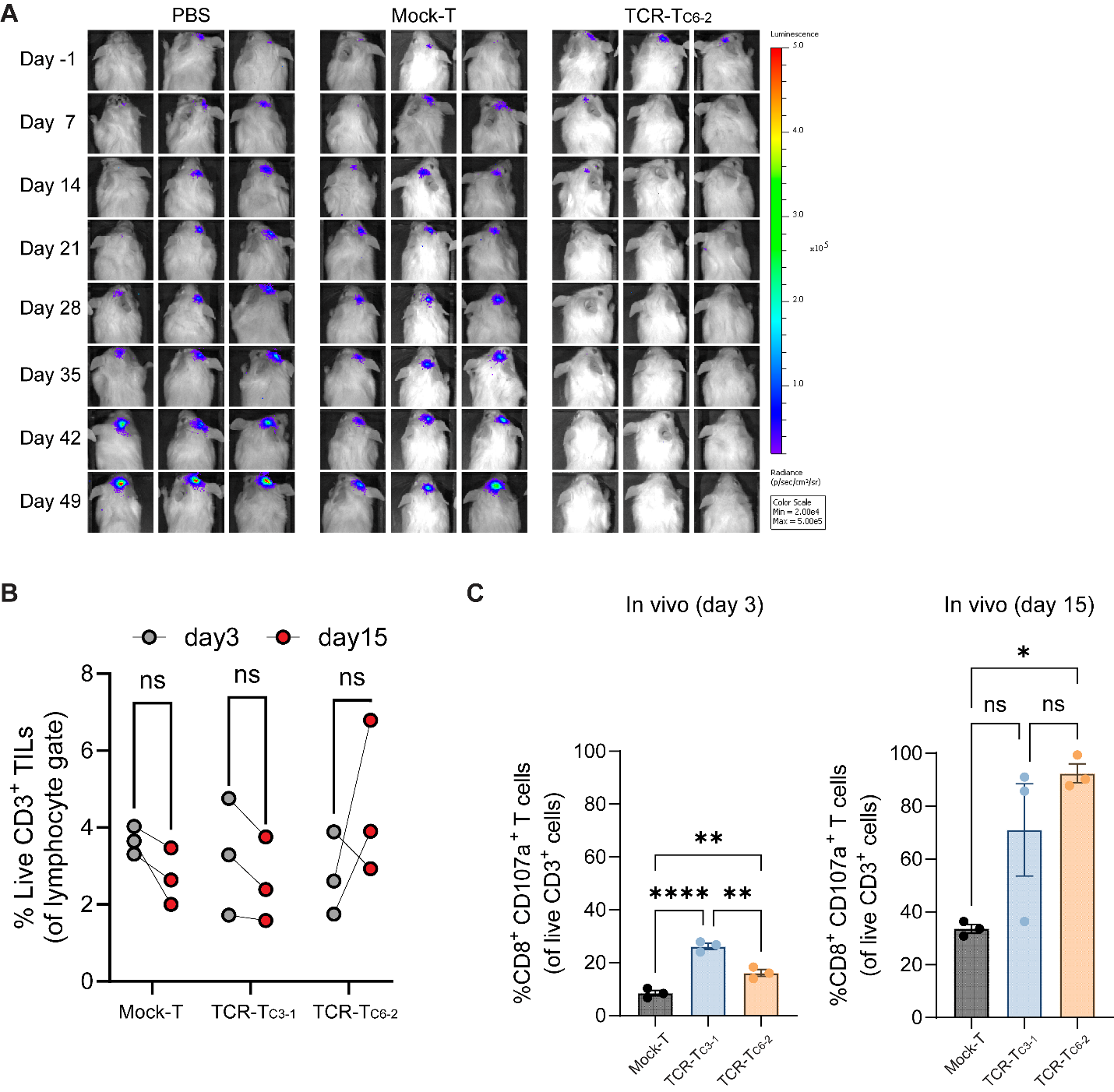


**Supplementary Figure 13. TCR-T suppresses adult GBM patient-derived RCAN1-4^pos^ GBM in vivo.**

A, Weekly alteration of bioluminescence imaging signal in SF10360 (adult GBM) xenograft murine model after TCR-T transfer. B, In vivo persistence and viability of intracranially transferred TCR-T cells isolated from the brains of GBM-bearing NSG mice at 3 and 15 days post-transfer. C, Analysis of tumor-infiltrating human CD8^+^ T cells from intracranial KNS-42 tumors. CD107a expression on transferred T cells is shown at day 3 (left) and day 15 (right) post-transfer. Statistics: two-way ANOVA with Tukey’s test; *p<0.05, **p<0.01, ****p<0.0001 vs. mock.
