# Supplementary material for: C/EBPβ-induced alternative splicing of RCAN1 generates a potent TCR-T target in mesenchymal glioblastoma
Source: Cell Mol Immunol. 2025 Dec 23;23(1):94–113. doi: 10.1038/s41423-025-01360-0 (PMC12753782; doi:10.1038/s41423-025-01360-0)
Supplement: Supplementary file 2 — unprocessed original images of Western blots [file 41423_2025_1360_MOESM2_ESM.pdf]

unprocessed original images of western blots

For **Figure 2B**

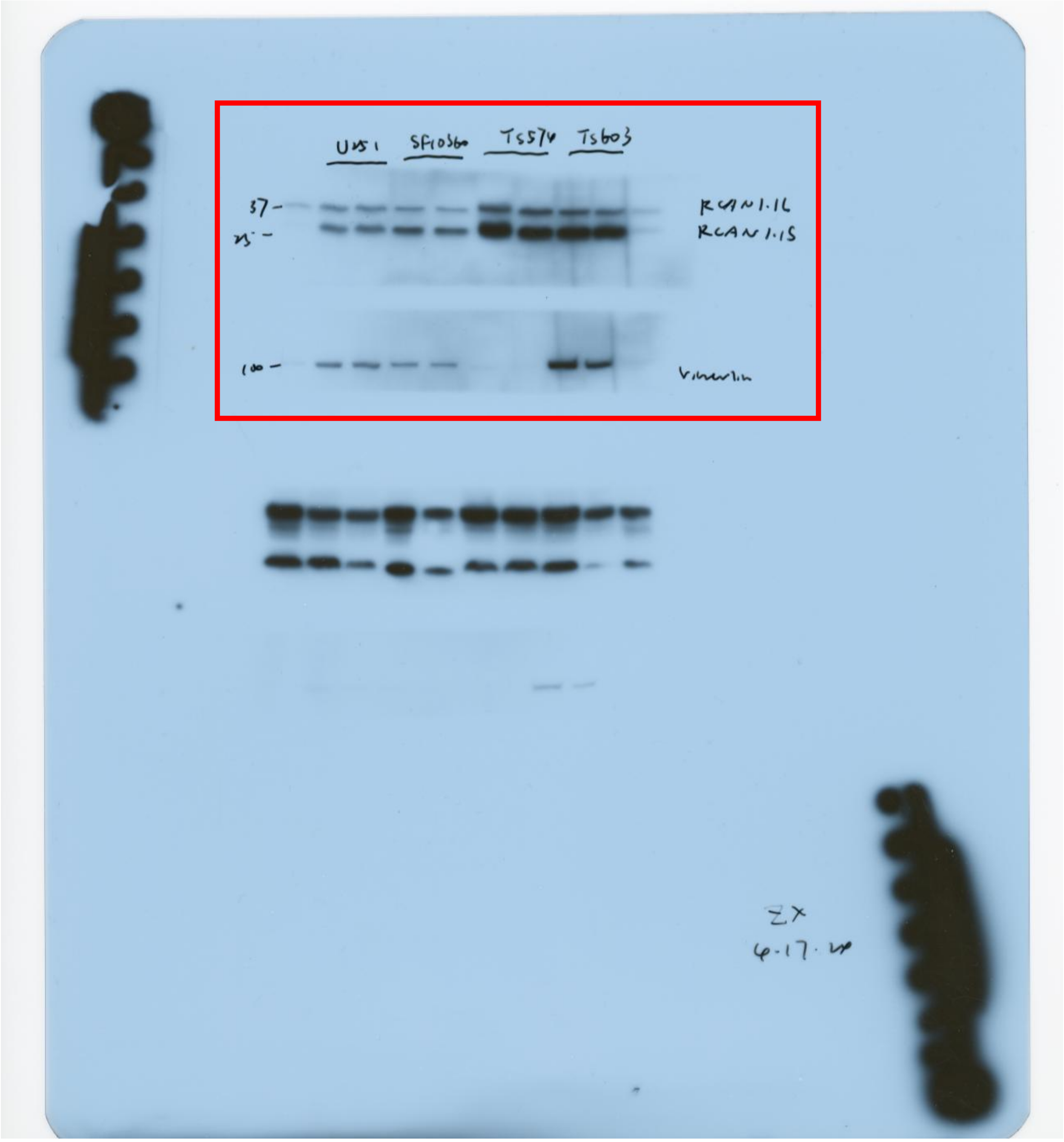

For **Figure 2B**

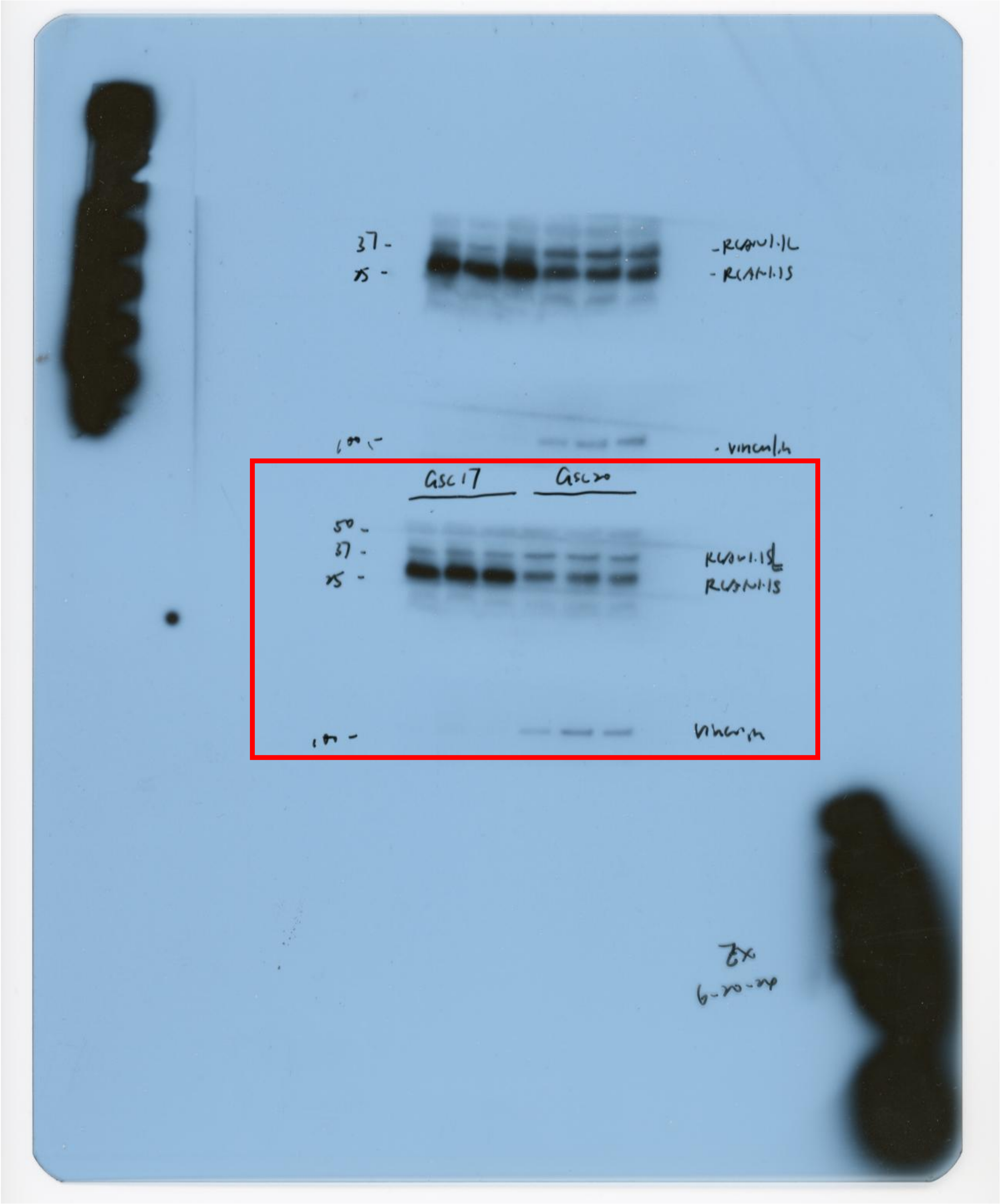

For **Figure 2C**

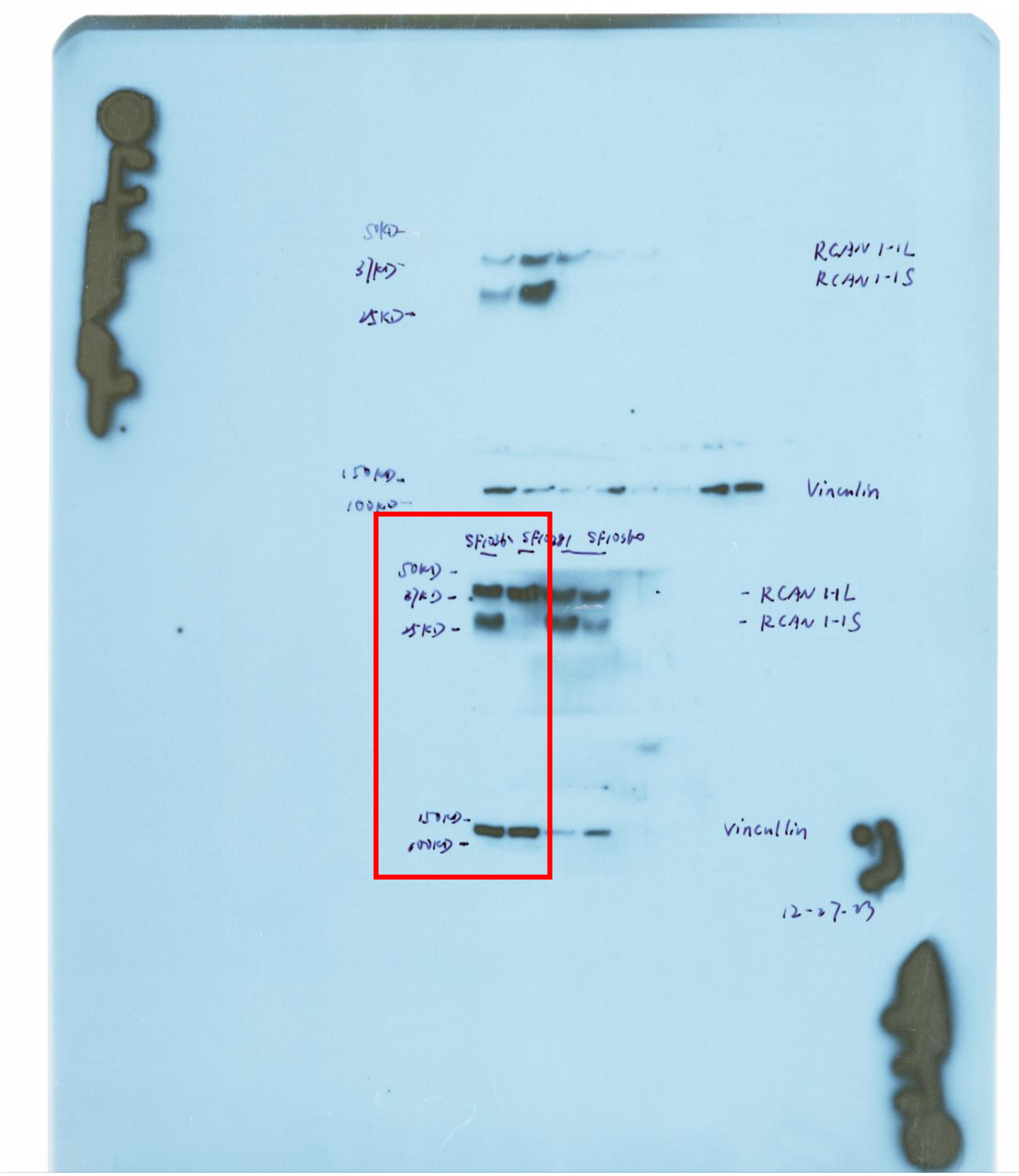

For **Figure 2E**

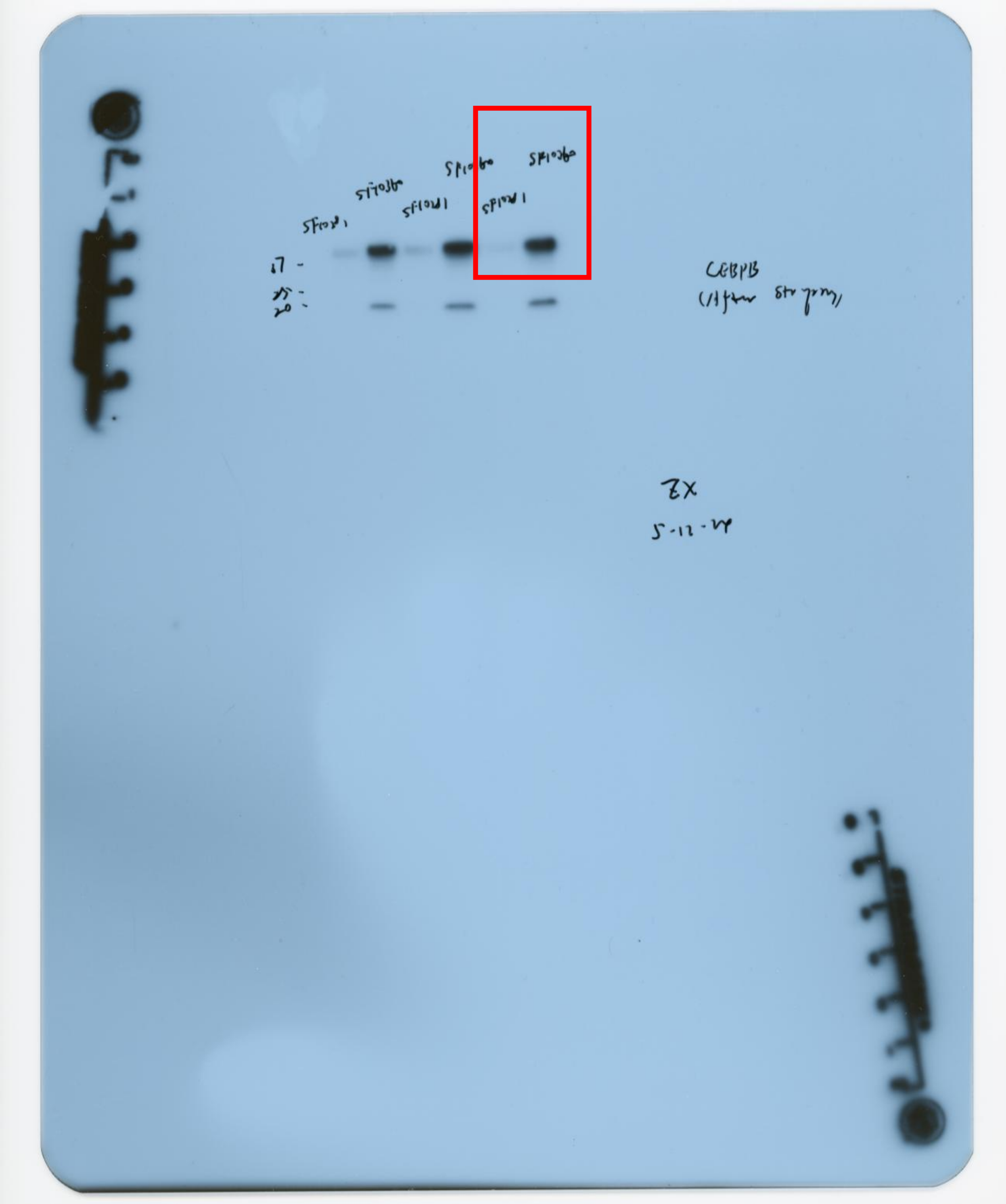

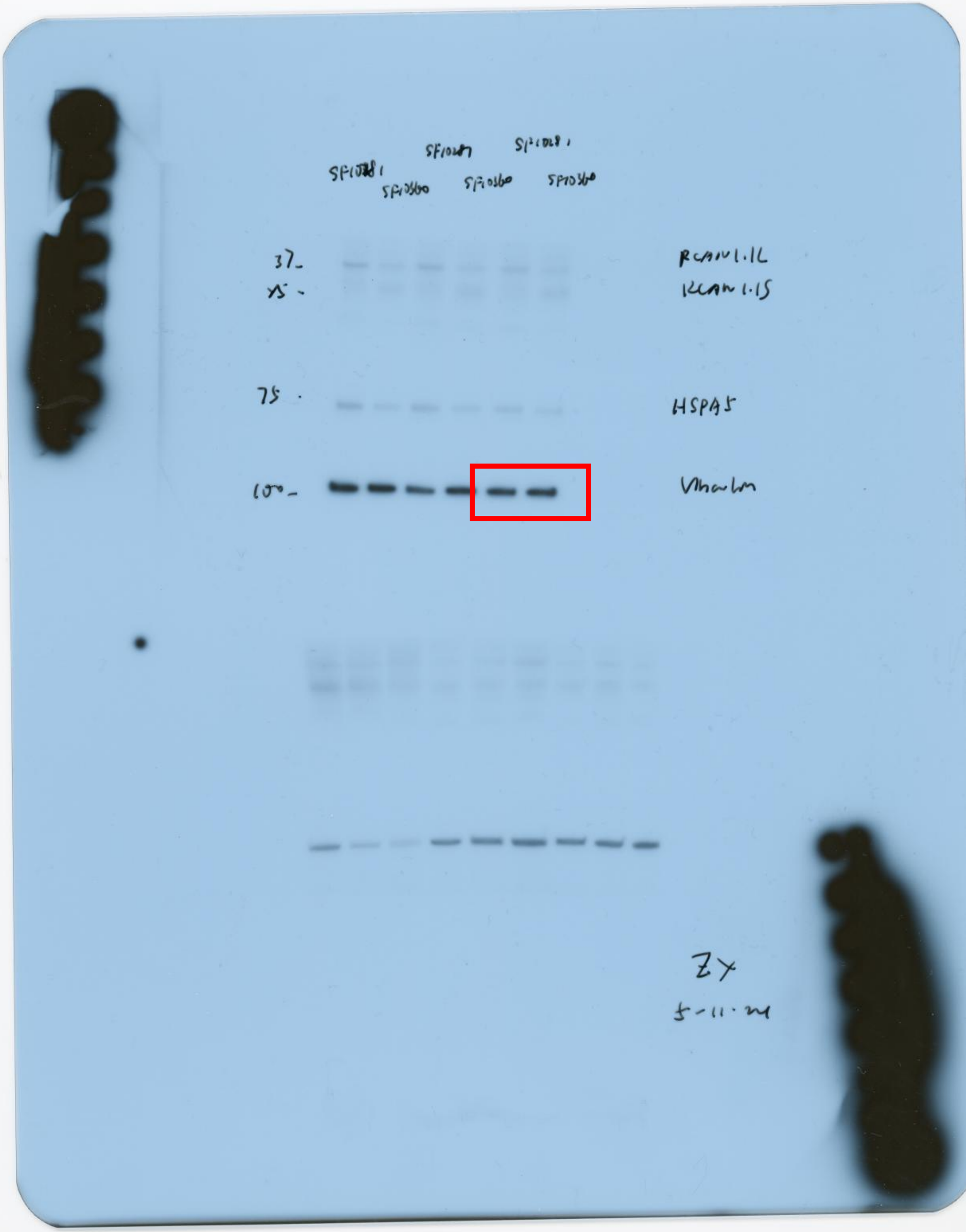

SF10281
SF10360
SF10281
SF10360
SF10281
SF10360
37
25
75
100
RCAN1.1L
RCAN1.1S
HSPA5
Vinculin
ZY
5-11-24

For **Figure 2G**

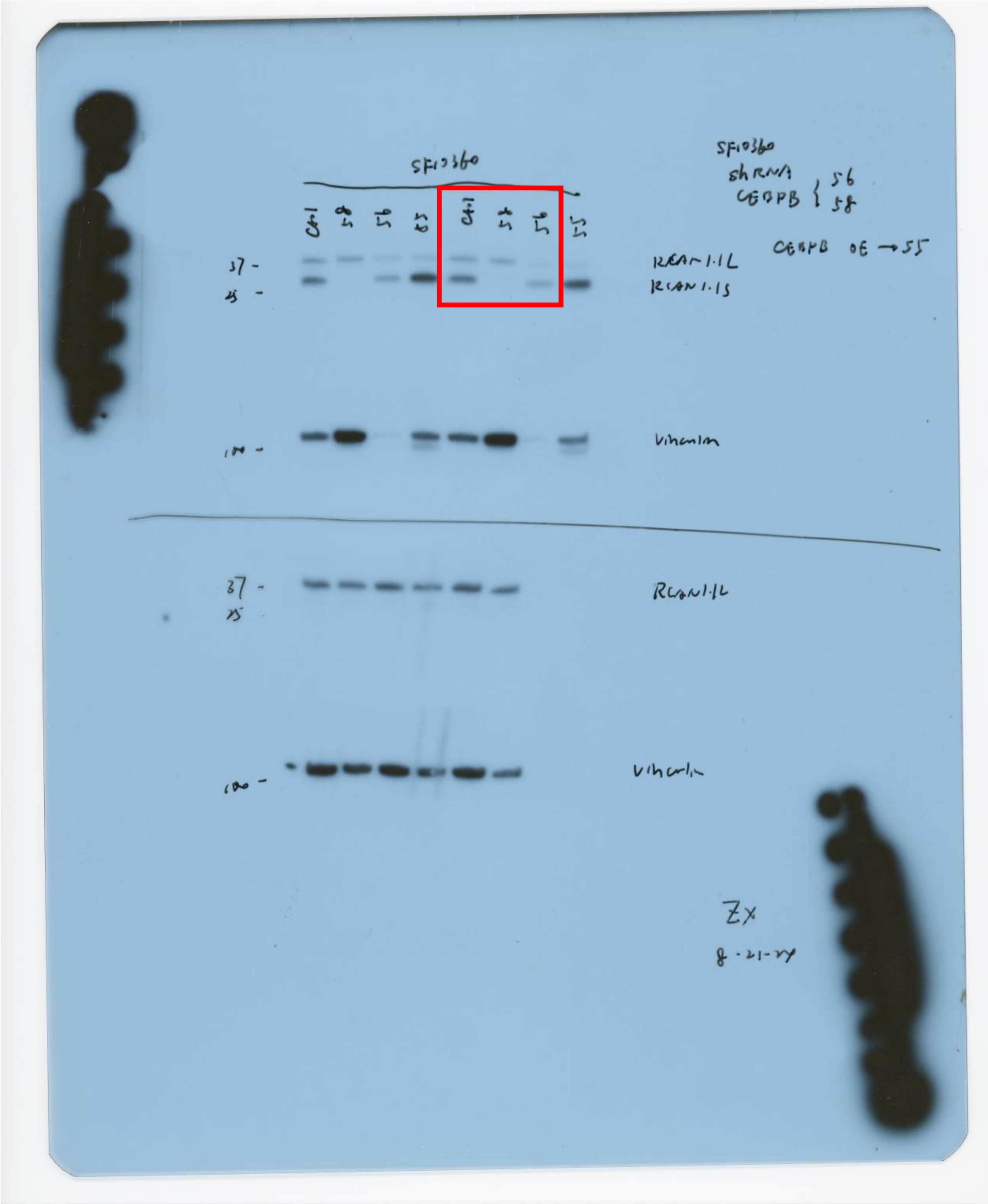

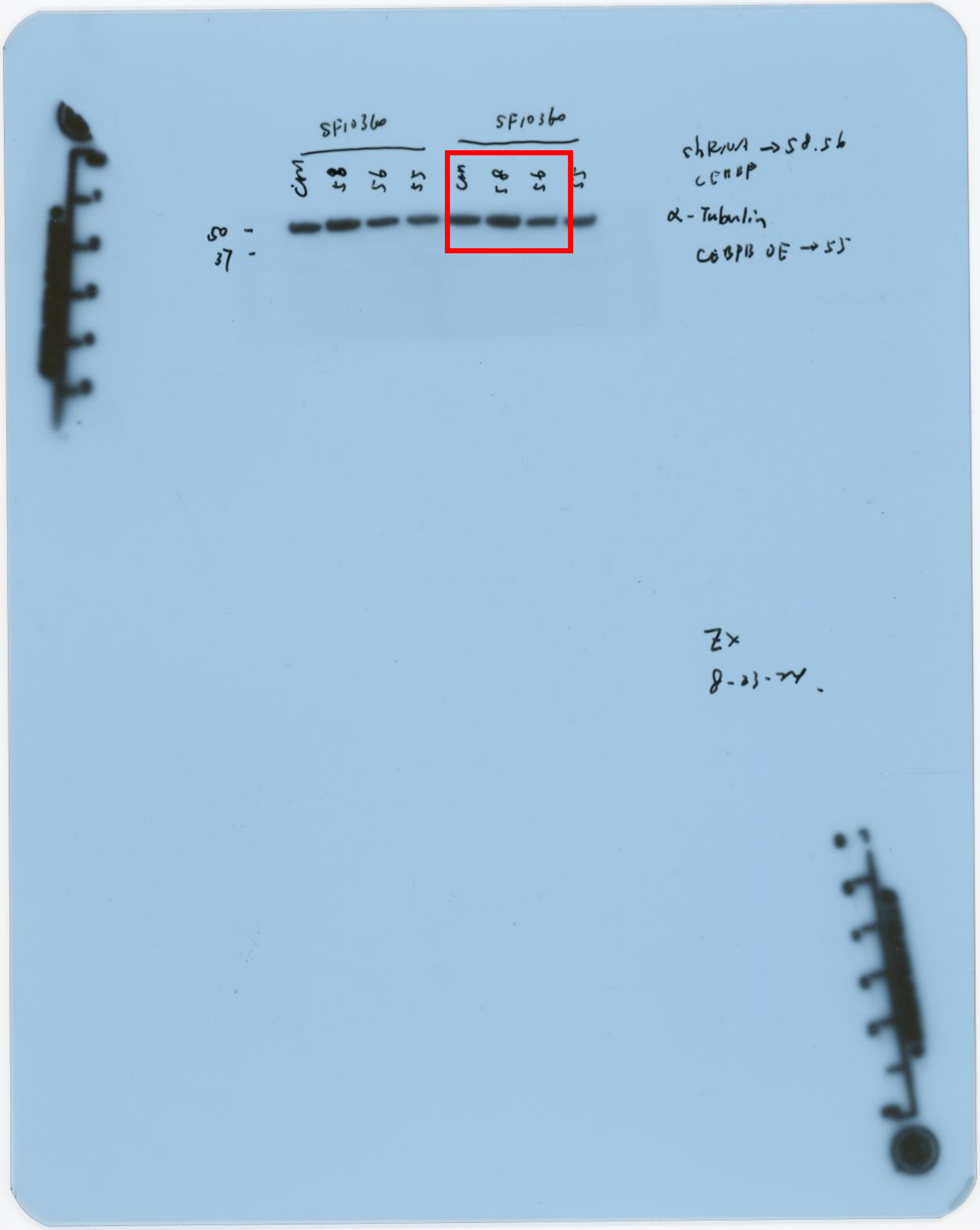
SF10360
SF10360
con
58
56
55
con
58
56
55
50 -
37 -
shRNA → 58.56
CEBPB
α-Tubulin
CEBPB OE → 55
ZX

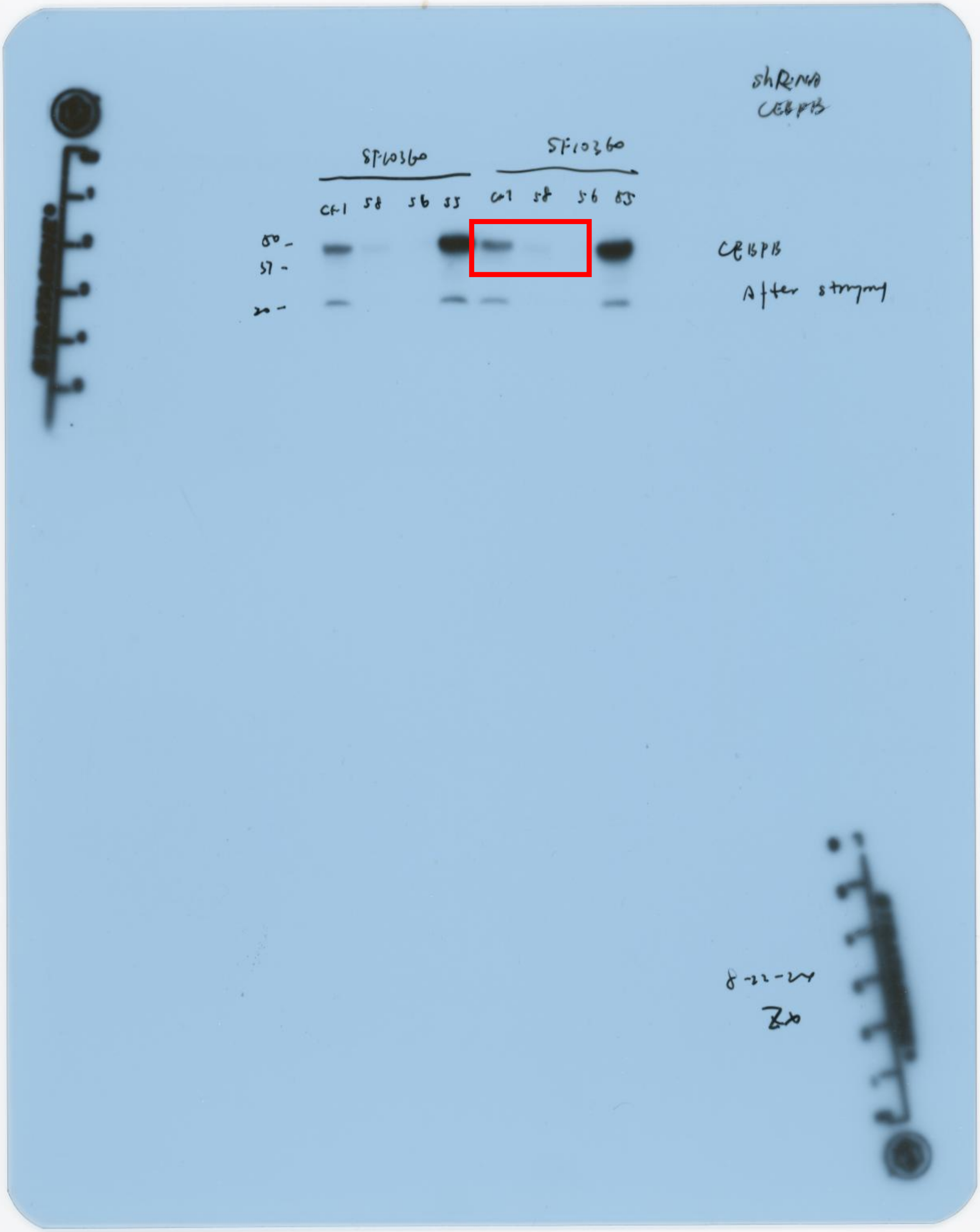
shRNA
CEBPB
SF10360
SF10360
Ctl 58 56 55
Ctl 58 56 55
50
37
20
CEBPB
8-22-24

For **Figure 2H**

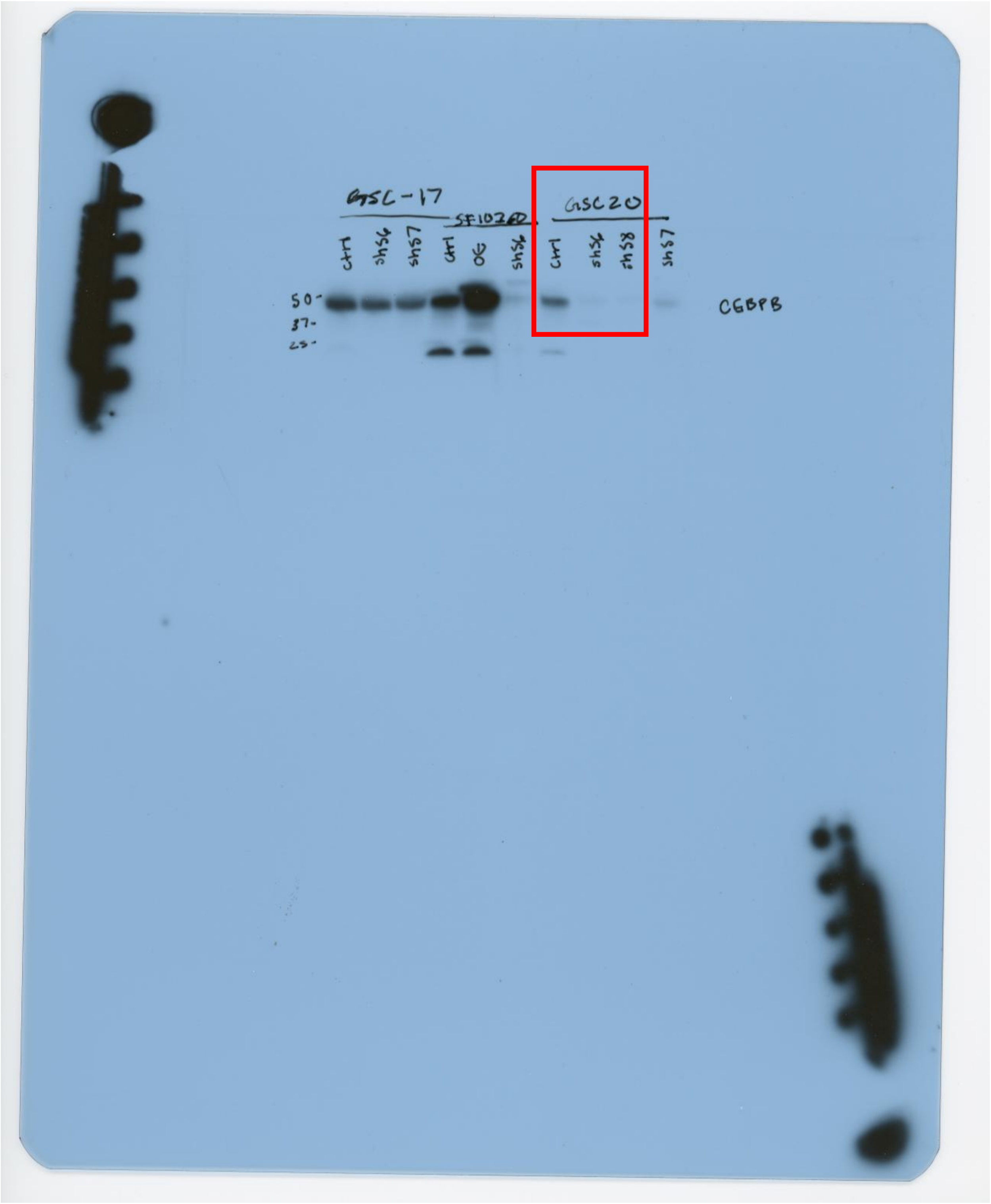

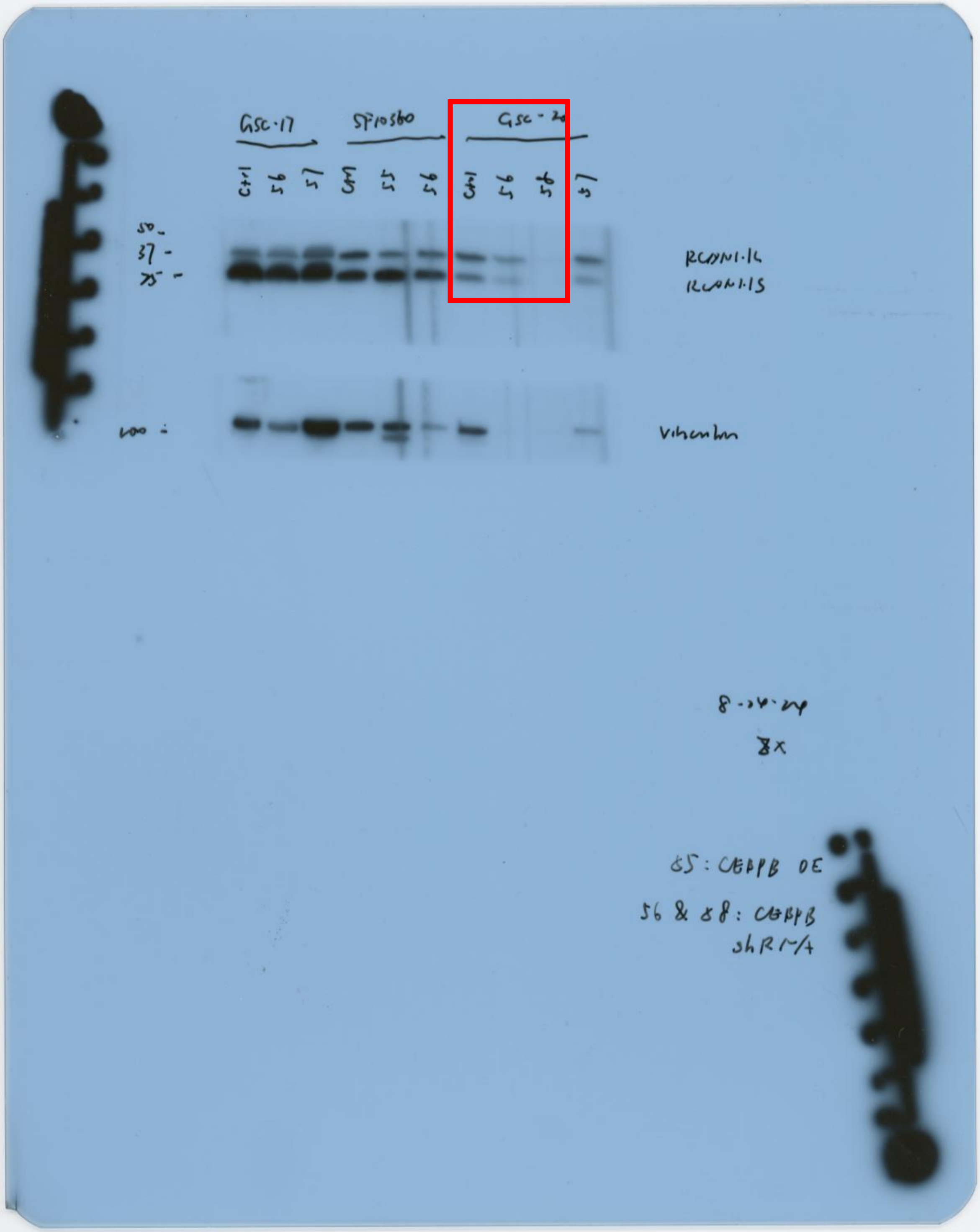
GSC-17
50
37
25
100
RCAN1.1L
RCAN1.1S
Vinculin
8-24-24
8X
55: CEBPB OE
56 & 58: CEBPB shRNA

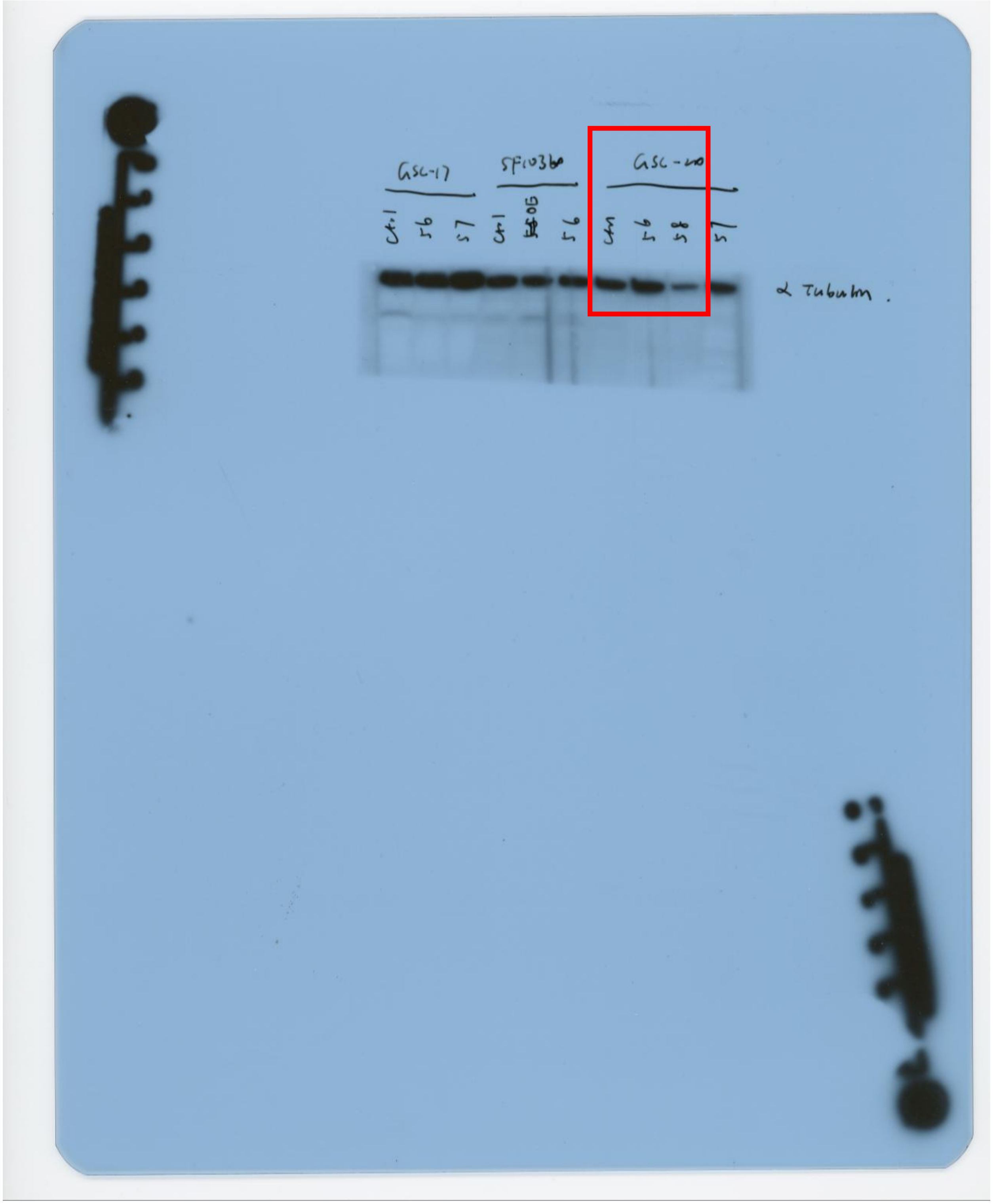
GSC-17
Ctrl
S6
S7
Ctrl
S6
Ctrl
S6
S8
S7
α Tubulin.

For **Figure 2I**

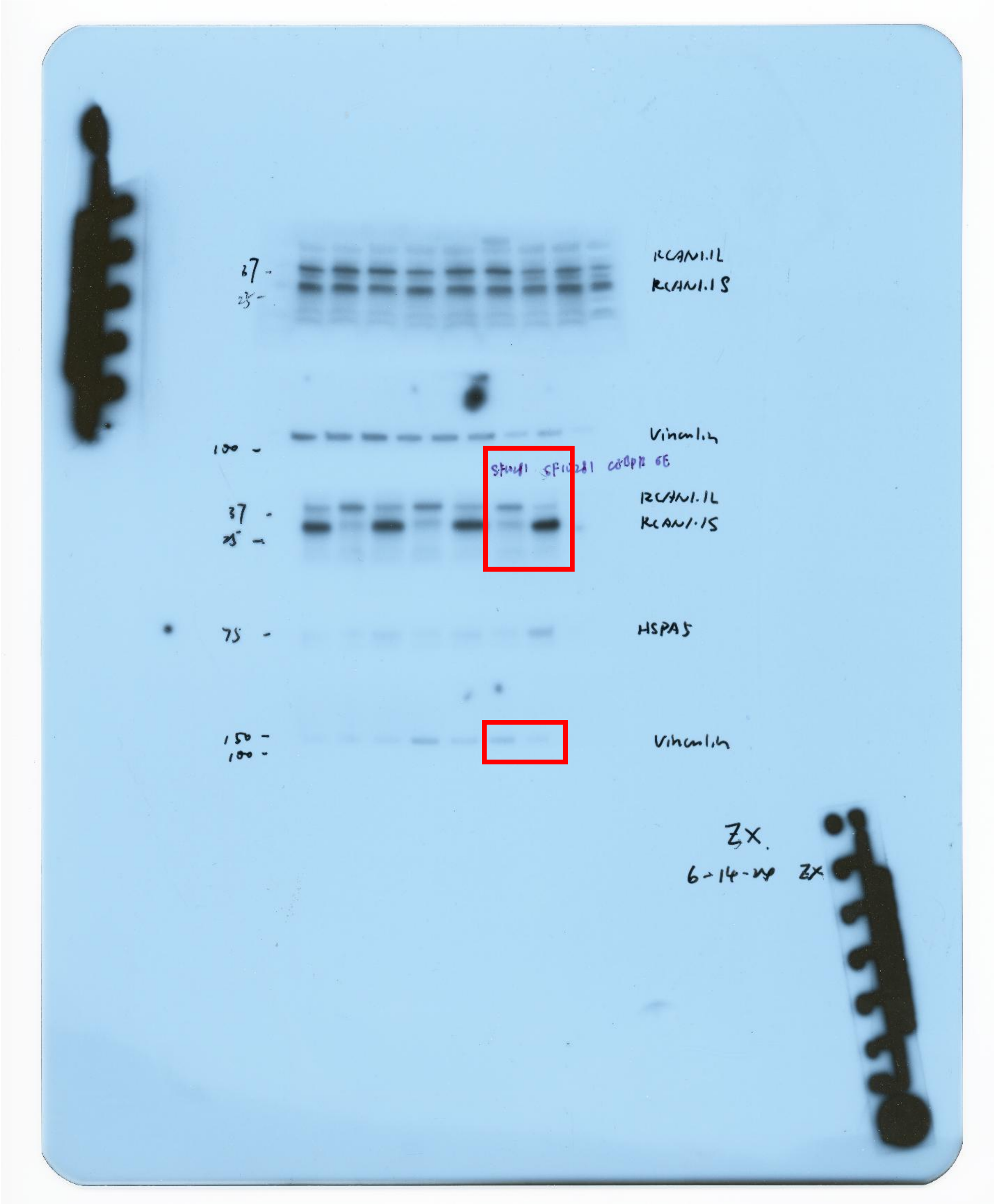

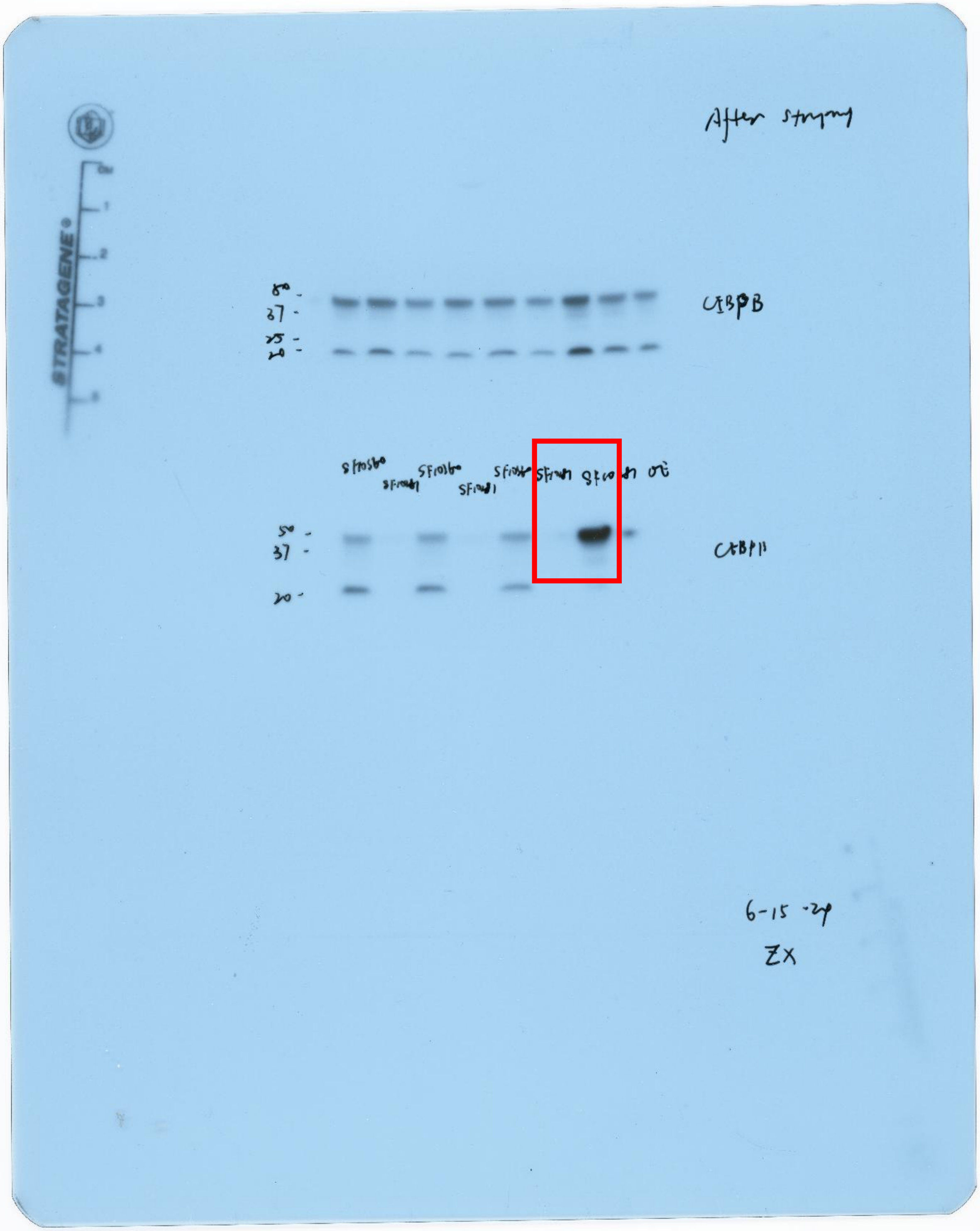

STRATAGENE
After stripping
50
37
25
20
CEBPB
50
37
20
6-15-24
ZX

For **Supplementary Figure 3I**

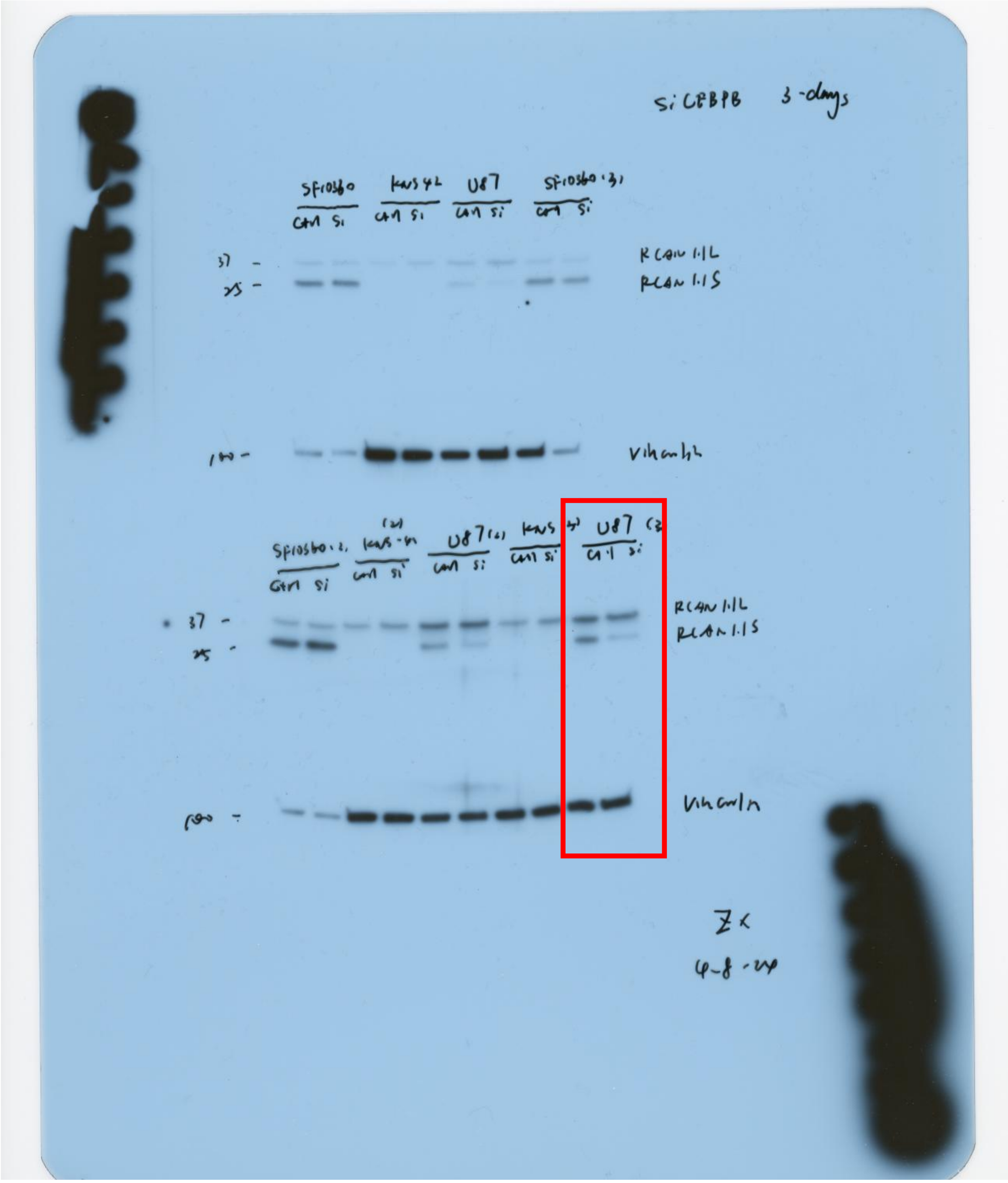

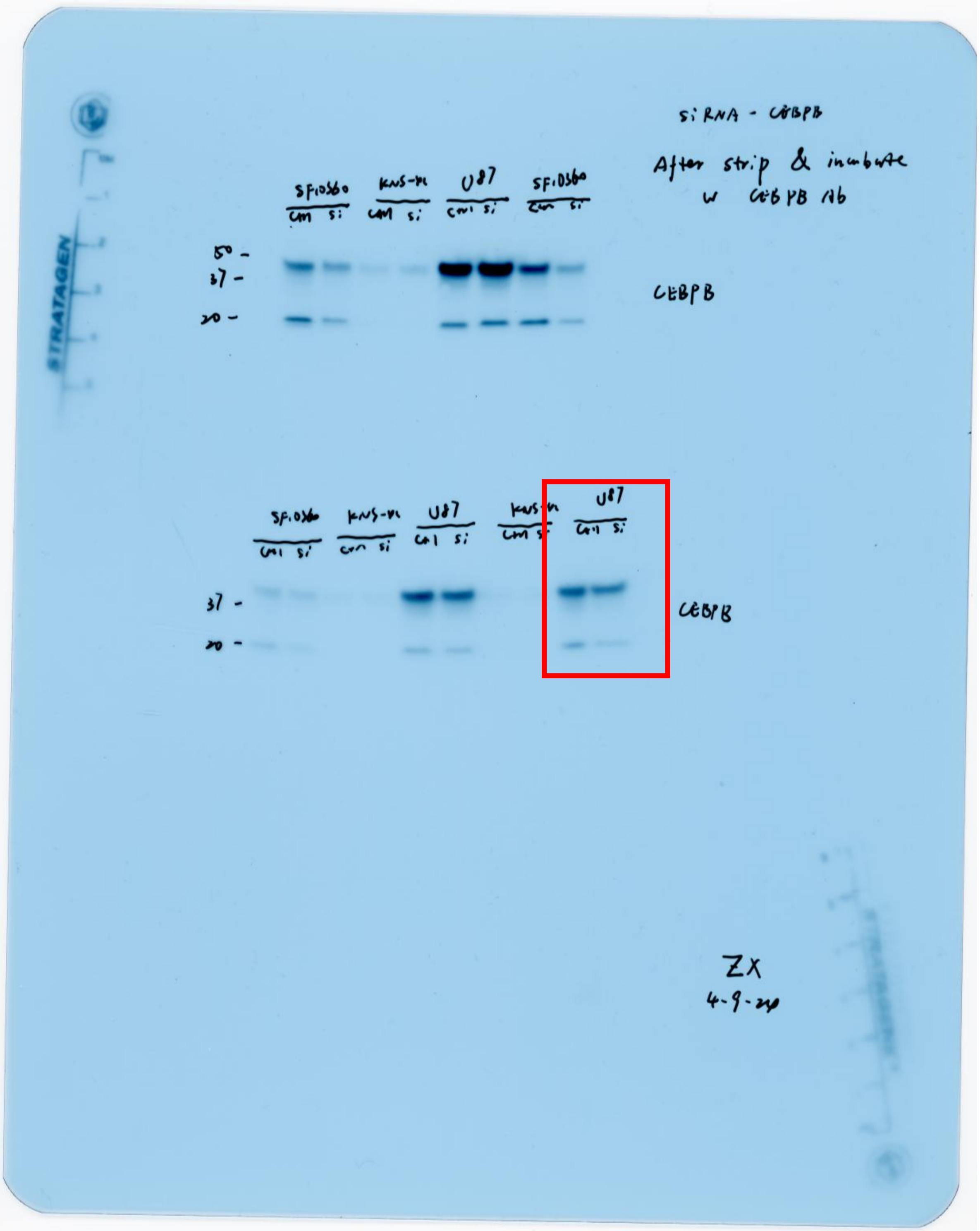

siRNA - CEBPB
After strip & incubate w CEBPB Ab
U87
Ctrl si
50 -
37 -
20 -
CEBPB
U87
U87
Ctrl si
37 -
20 -
CEBPB
ZX
STRATAGEN

For **Supplementary Figure 3K**

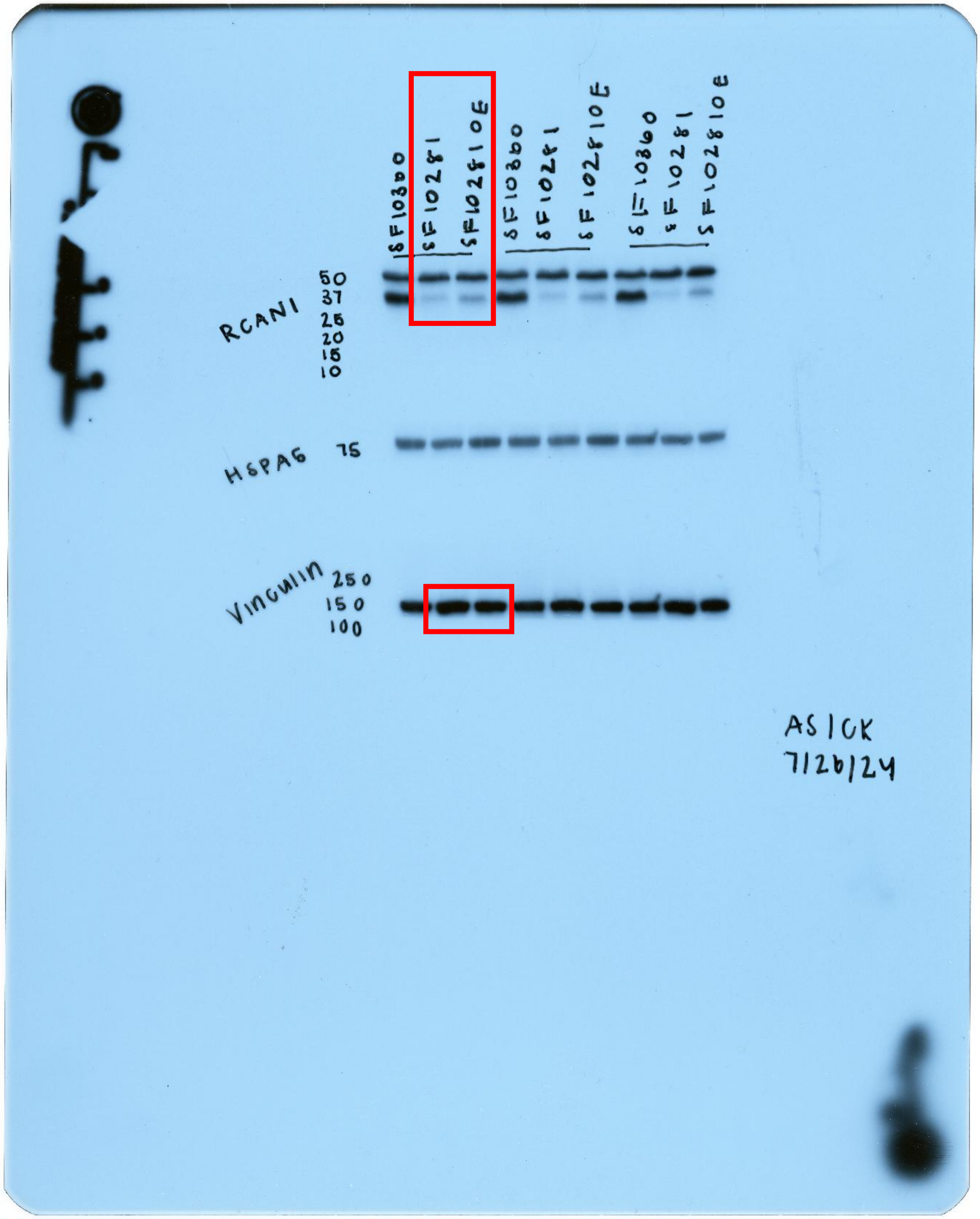

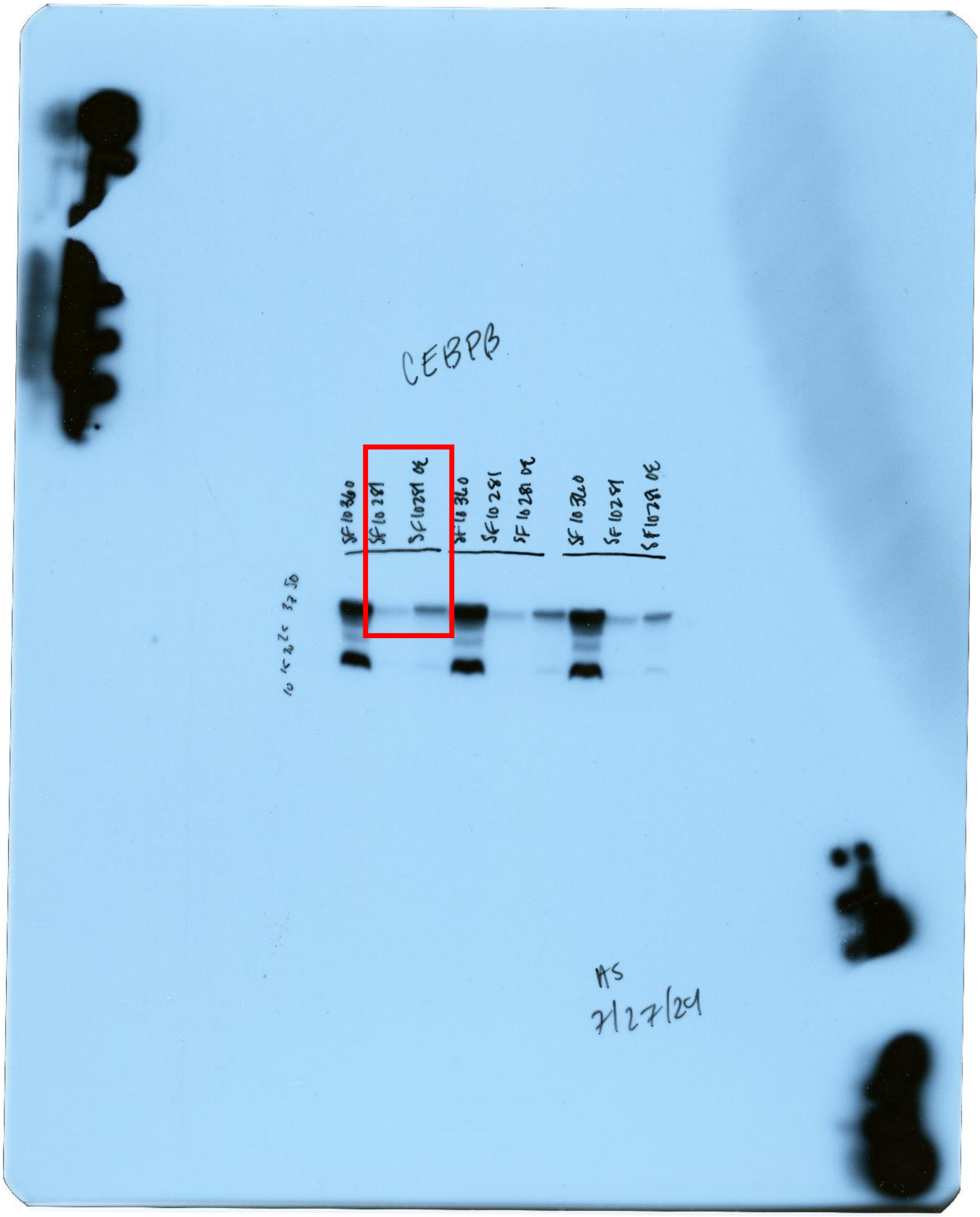
CEBPB
SF10360
SF10281
SF10281 OE
SF10360
SF10281
SF10281 OE
SF10360
SF10281
SF10281 OE
AS
7/27/24

For **Supplementary Figure 3L**

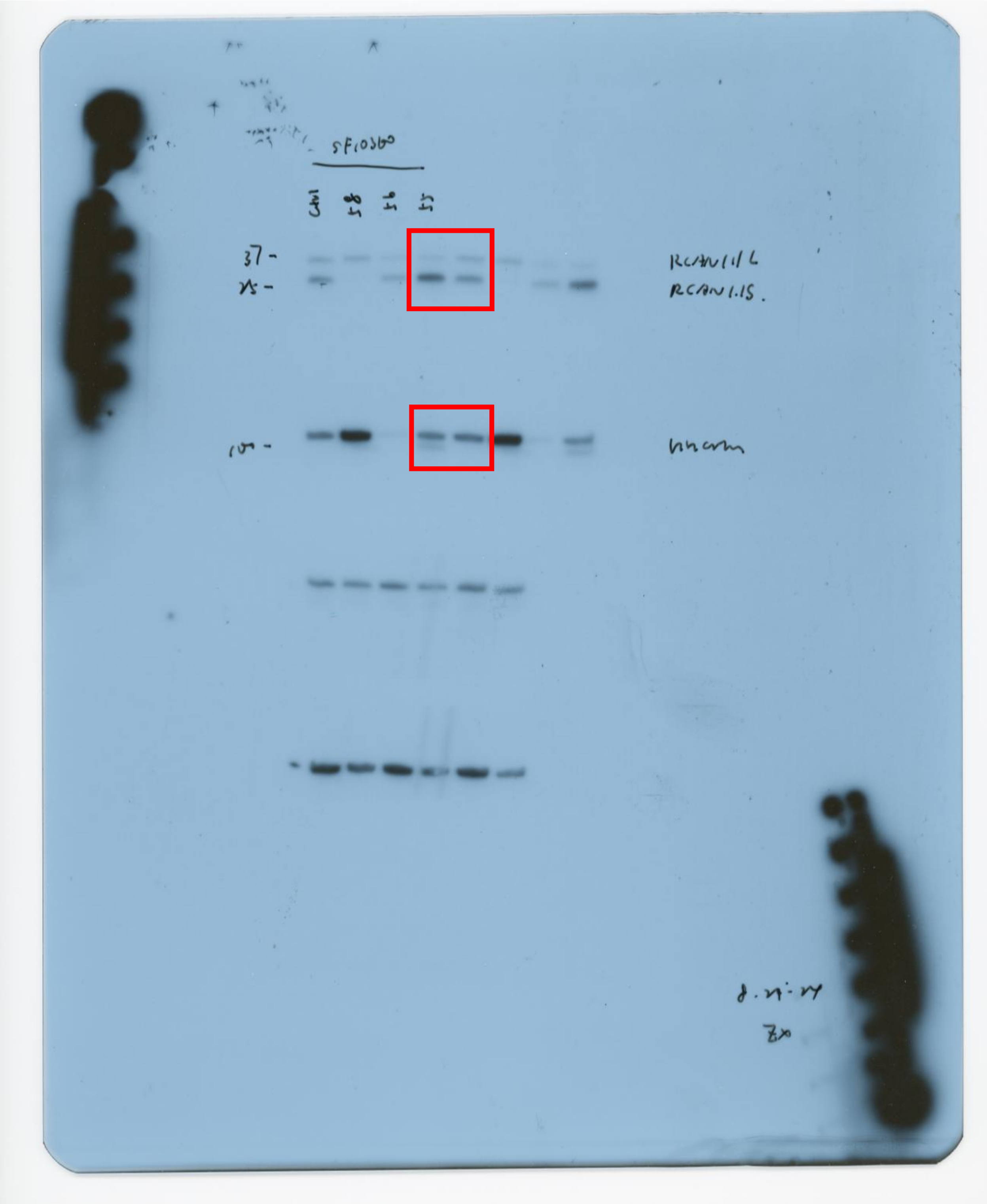

The plot was flipped in the submission to make the ctrl at the first position.

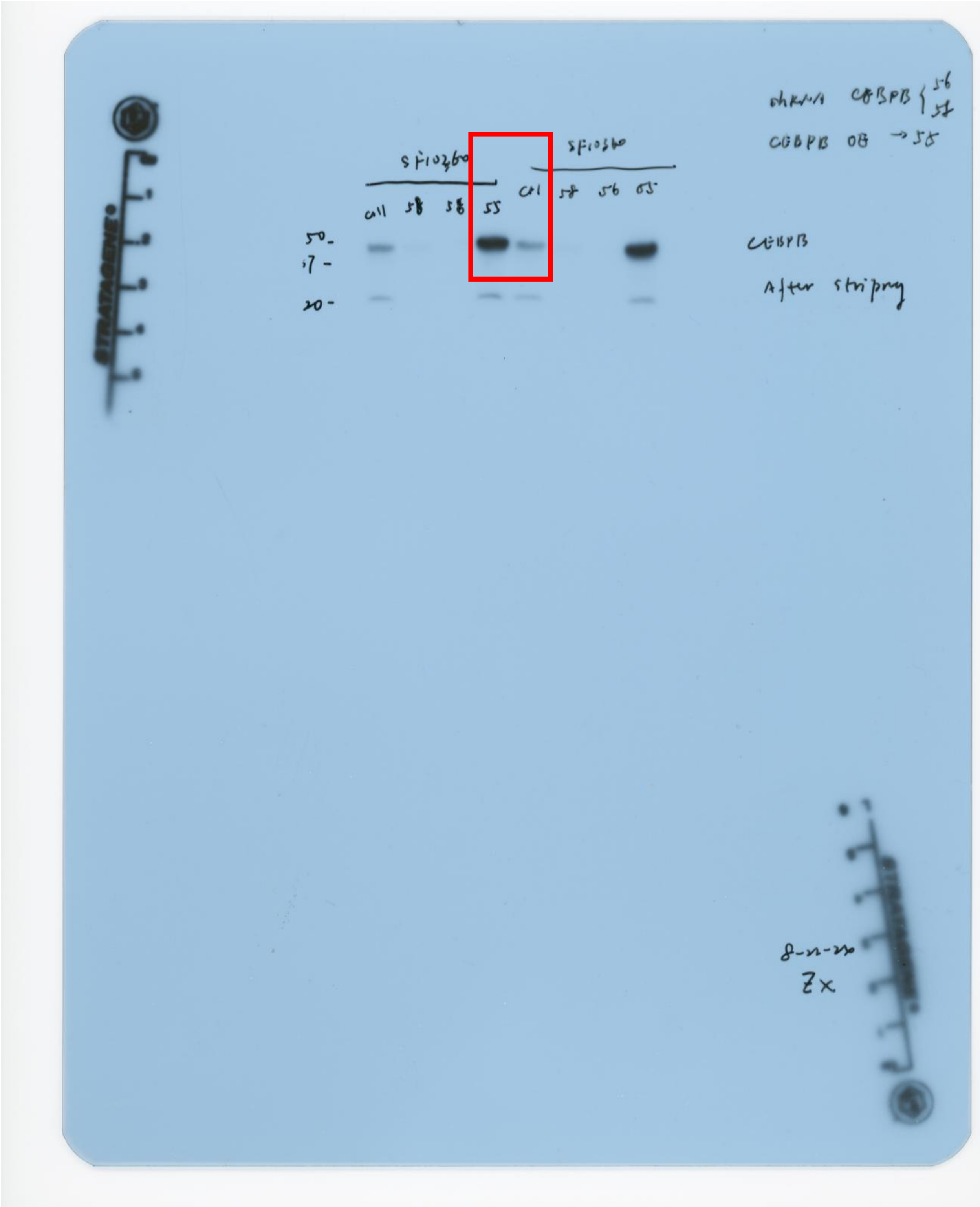

The plot was flipped in the submission to make the ctrl at the first position.

For **Supplementary Figure 7F**

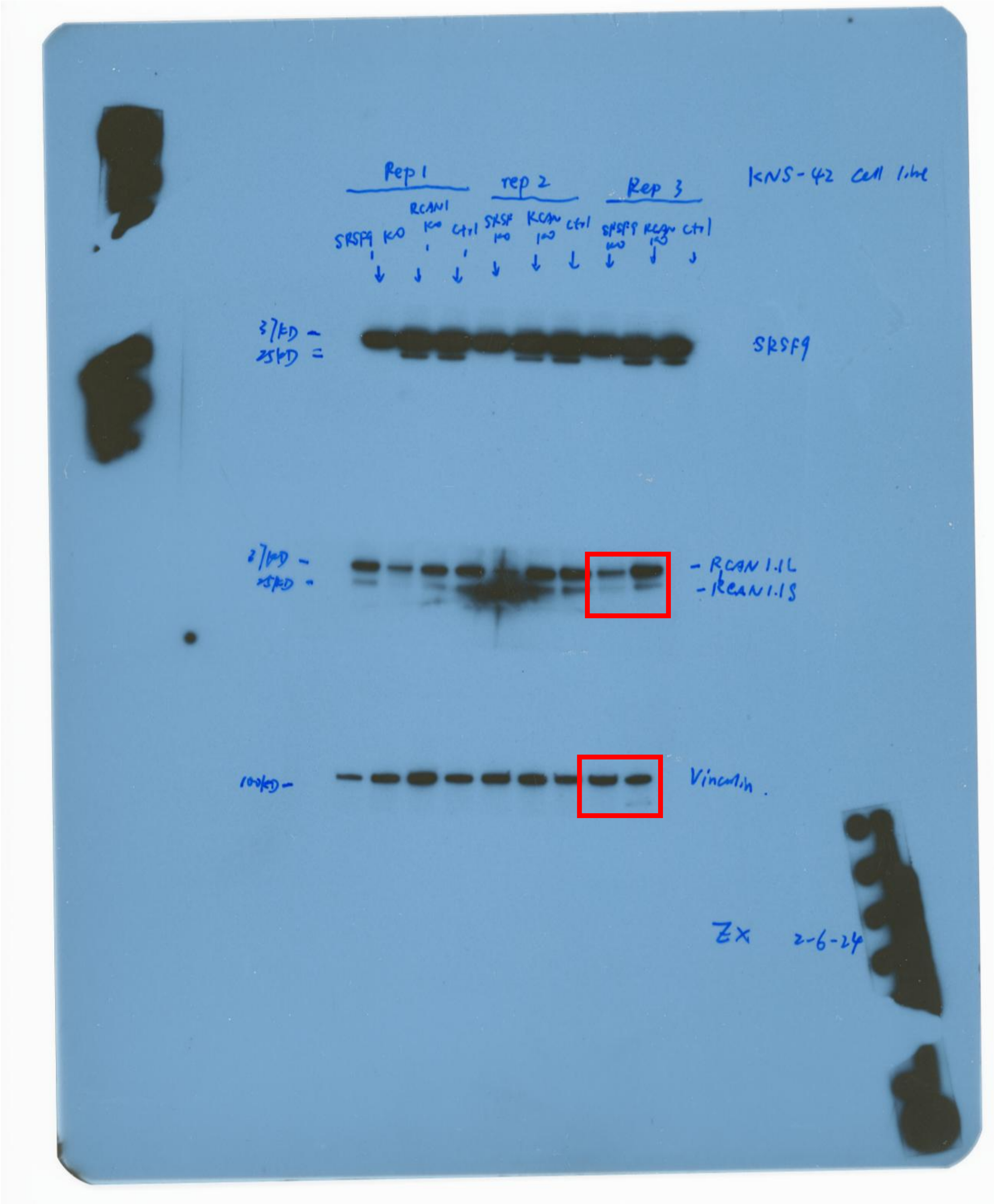

The plot was flipped in the submission to make the ctrl at the first position.

For Revision

For **Supplementary Figure 2H**

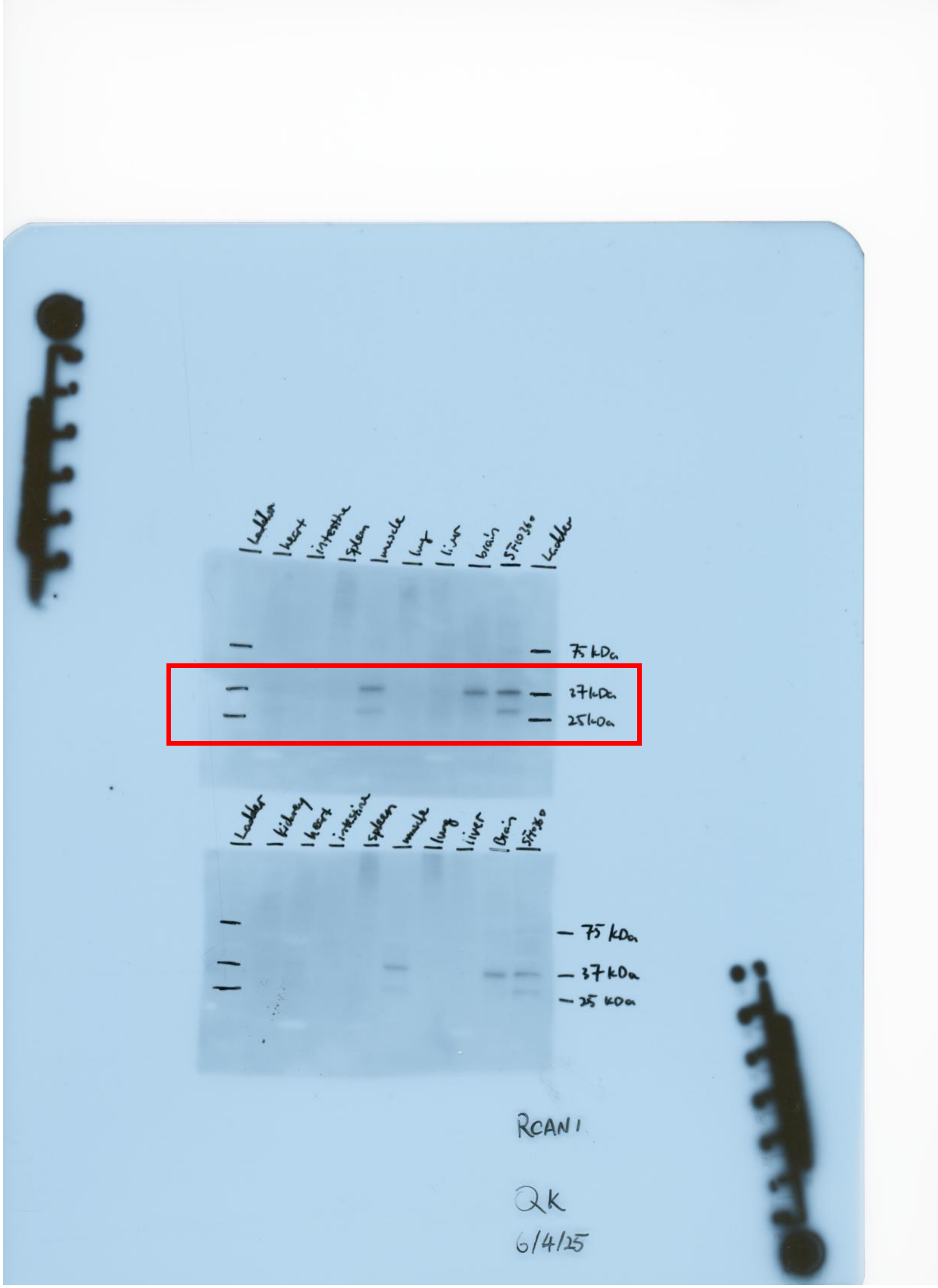

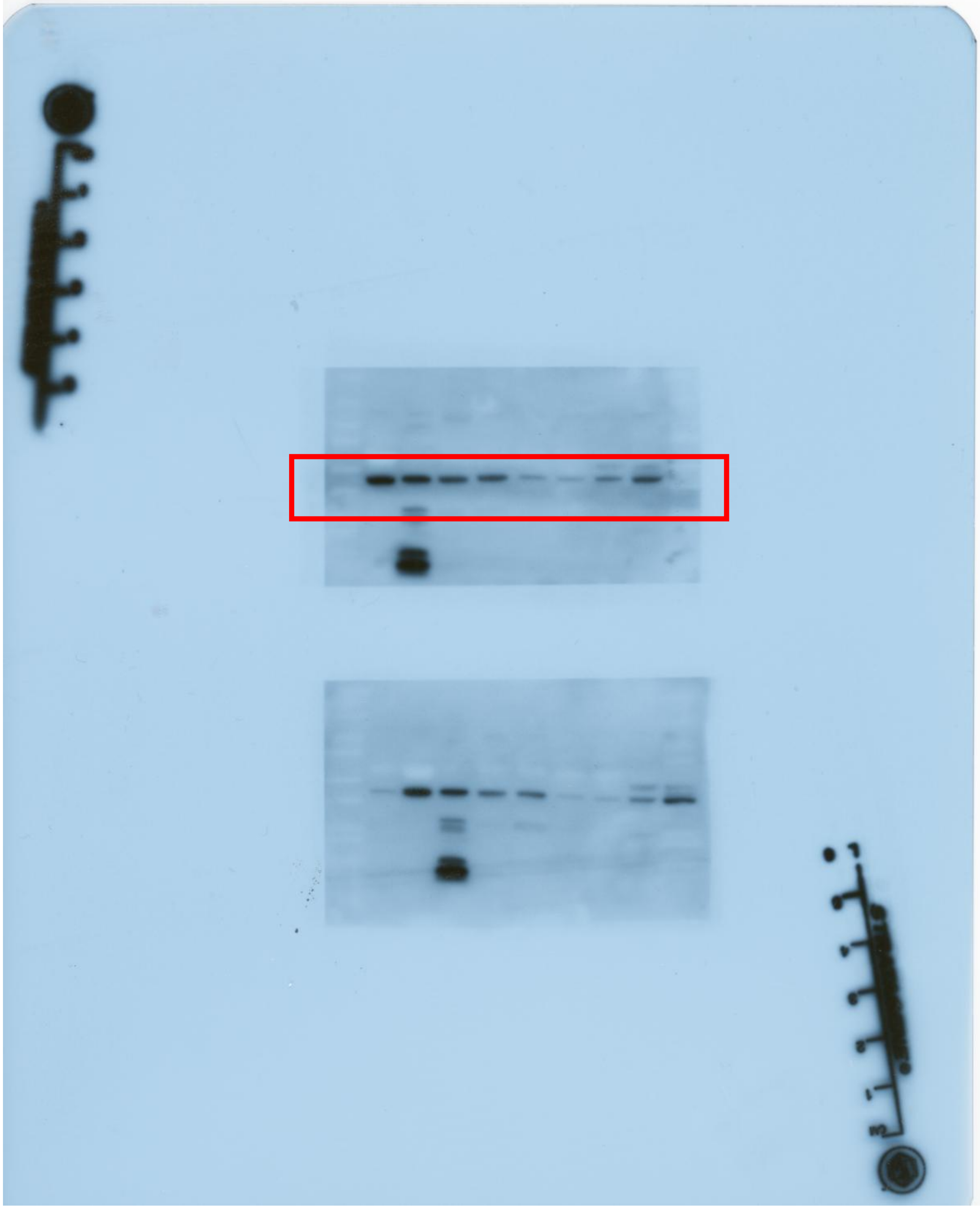

For **Supplementary Figure 3G**

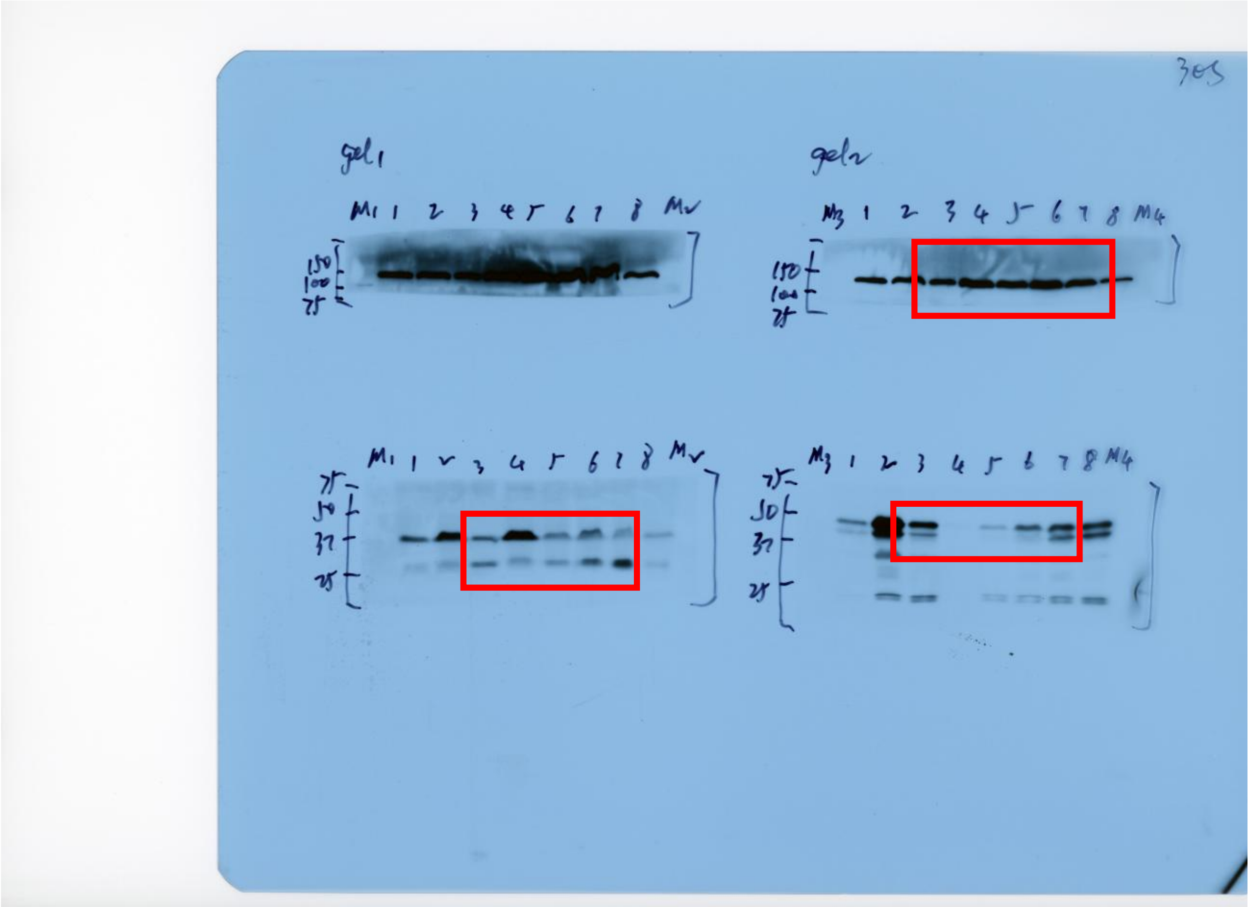

For **Supplementary Figure 10F**

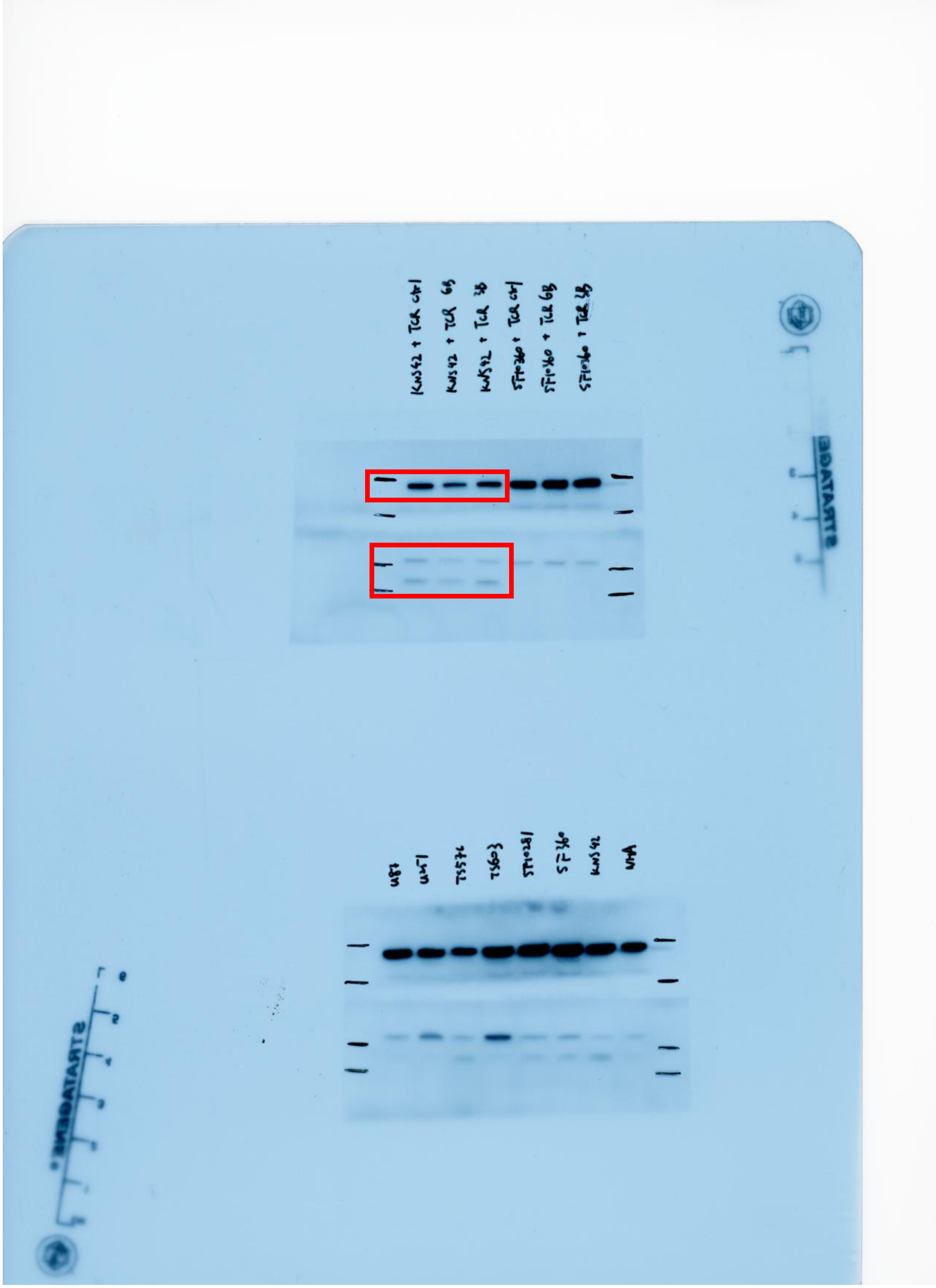

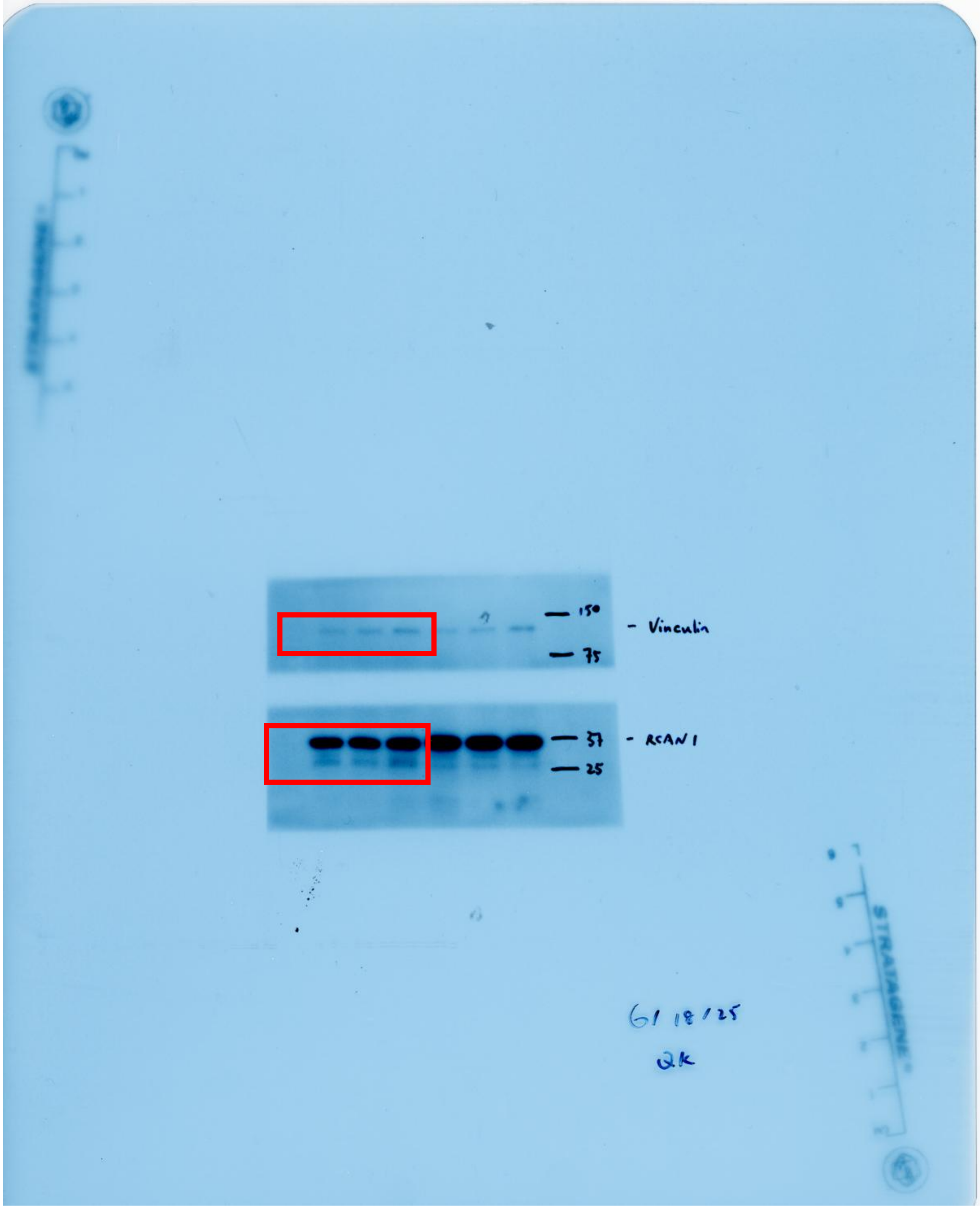

150
Vinculin
75
37
RCAN1
25
6/18/25
QK
